# Shear stress control of vascular leaks and atheromas through Tie2 activation by VE‐PTP sequestration

**DOI:** 10.15252/emmm.202216128

**Published:** 2023-02-06

**Authors:** Keisuke Shirakura, Peter Baluk, Astrid F Nottebaum, Ute Ipe, Kevin G Peters, Donald M McDonald, Dietmar Vestweber

**Affiliations:** ^1^ Max Planck Institute for Molecular Biomedicine Münster Germany; ^2^ Cardiovascular Research Institute, UCSF Helen Diller Family Comprehensive Cancer Center, and Department of Anatomy University of California, San Francisco San Francisco CA USA; ^3^ Aerpio Therapeutics, Inc. Blue Ash OH USA

**Keywords:** aorta, atherosclerosis, endothelial junctions, laminar flow, vascular leakage, Cardiovascular System, Vascular Biology & Angiogenesis

## Abstract

Vascular endothelial protein tyrosine phosphatase (VE‐PTP) influences endothelial barrier function by regulating the activation of tyrosine kinase receptor Tie2. We determined whether this action is linked to the development of atherosclerosis by examining the influence of arterial shear stress on VE‐PTP, Tie2 activation, plasma leakage, and atherogenesis. We found that exposure to high average shear stress led to downstream polarization and endocytosis of VE‐PTP accompanied by Tie2 activation at cell junctions. In aortic regions with disturbed flow, VE‐PTP was not redistributed away from Tie2. Endothelial cells exposed to high shear stress had greater Tie2 activation and less macromolecular permeability than regions with disturbed flow. Deleting endothelial VE‐PTP in VE‐PTP^iECKO^ mice increased Tie2 activation and reduced plasma leakage in atheroprone regions. ApoE^−/−^ mice bred with VE‐PTP^iECKO^ mice had less plasma leakage and fewer atheromas on a high‐fat diet. Pharmacologic inhibition of VE‐PTP by AKB‐9785 had similar anti‐atherogenic effects. Together, the findings identify VE‐PTP as a novel target for suppression of atherosclerosis.

## Graphical abstract

Laminar shear stress prevents endothelial leakage and atheroma formation at atheroprotected areas through Tie2 activation, caused by VE‐PTP polarization and endocytosis. VE‐PTP sequestration is absent at atheroprone sites, which can be compensated by inhibiting VE‐PTP, suppressing atherosclerosis.

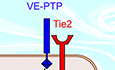

## Introduction

Vascular endothelial protein tyrosine phosphatase (VE‐PTP, receptor‐type protein tyrosine phosphatase beta, *PTPRB*) is a receptor type phosphatase expressed in vascular endothelial cells that controls permeability and angiogenesis (Vestweber, 2021). The tyrosine kinase receptor Tie2 involved in the regulation of endothelial barrier function is among the VE‐PTP substrates (Fachinger *et al*, 1999; Winderlich *et al*, 2009; Shen *et al*, 2014; Frye *et al*, 2015; Drexler *et al*, 2019). The adherens junction protein vascular endothelial cadherin (VE‐cadherin) (Nawroth *et al*, 2002; Nottebaum *et al*, 2008; Broermann *et al*, 2011; Frye *et al*, 2015), VEGFR2, and the GTPase exchange factor FGD5 (Mellberg *et al*, 2009; Braun *et al*, 2019) are among other relevant VE‐PTP substrates.

VE‐PTP reduces Tie2 activity by promoting dephosphorylation (Fachinger *et al*, 1999; Winderlich *et al*, 2009). Essential functions of the phosphatase in vascular development are indicated by embryonic lethality accompanying developmental vascular defects after VE‐PTP inactivation or gene ablation (Baumer *et al*, 2006; Dominguez *et al*, 2007; Winderlich *et al*, 2009). Importantly, VE‐PTP interaction with Tie2 influences vascular stability. Tie2 activation following genetic or pharmacological inhibition of VE‐PTP suppresses vascular leakage (Shen *et al*, 2014; Frye *et al*, 2015) and protects against endothelial cell activation (Shen *et al*, 2014; Carota *et al*, 2019). Of clinical relevance, VE‐PTP inhibition is beneficial in disease models of retinopathy, diabetic nephropathy, glaucoma, hypertension, and metastasis (Goel *et al*, 2013; Shen *et al*, 2014; Carota *et al*, 2019; Li *et al*, 2020; Siragusa *et al*, 2021) and in human diabetic macular edema (Campochiaro *et al*, 2016).

The cellular distribution of VE‐PTP changes in response to shear stress. When endothelial cells in culture are exposed to shear stress, VE‐PTP is redistributed to the downstream tip and internalized from the plasma membrane into endosomes (Mantilidewi *et al*, 2014). This change is dependent on the magnitude and duration of shear stress and has been interpreted as contributing to cell realignment and elongation to reduce mechanical stress (Mantilidewi *et al*, 2014). Shear stress in cultured cells can also promote VE‐PTP interaction with VE‐cadherin, which reduces VE‐cadherin phosphorylation in vascular fusion during development (Caolo *et al*, 2018). However, little is known about the influence of shear stress on VE‐PTP phosphatase activity and Tie2 signaling in the maintenance of endothelial barrier function and atherosclerosis pathogenesis.

Blood flow patterns and vascular architecture determine the nature and magnitude of shear forces that govern endothelial cell function and atheroma formation (Chiu & Chien, 2011; Mohamied *et al*, 2017). Endothelial cells of straight, unbranched segments of the aorta and other arteries are exposed to high average unidirectional, laminar shear stress that stabilizes endothelial junctions, supports endothelial barrier function, suppresses leakage, and protects against atherogenesis (*atheroprotection*; Davies, 1995; Gimbrone & Garcia‐Cardena, 2013). In contrast, endothelial cells of the inner curvature of the aortic arch and arterial branches and bifurcations are subjected to disturbed flow patterns with low average shear stress, where blood flow reverses, oscillates, circulates in vortices, or exhibits turbulence (Davies, 1995; Gimbrone & Garcia‐Cardena, 2013). Changing flow patterns can expose endothelial cells to shear stress in multiple directions during a cardiac cycle, where the dynamics are reflected by abrupt differences in endothelial morphology between adjacent endothelial cells (Davies, 1995). Regions of the aorta and other arteries with complex flow patterns have impaired endothelial barrier function, greater leakage of low‐density lipoproteins (LDL) and other plasma constituents, and are predisposed to the development of atherosclerosis (*atheroprone*; Davies, 1995; Gimbrone & Garcia‐Cardena, 2013).

Endothelial cells sense shear forces through mechanically sensitive proteins in the plasma membrane (Mack *et al*, 2017; Mehta *et al*, 2020). Mechanotransducers in endothelial cells include platelet endothelial cell adhesion molecule 1 (PECAM1), VE‐cadherin (Tzima *et al*, 2005), integrins (Xanthis *et al*, 2019), Piezo1 mechanosensitive ion channel (Li *et al*, 2014; Albarran‐Juarez *et al*, 2018), and others (Mack *et al*, 2017; Albarran‐Juarez *et al*, 2018; Mehta *et al*, 2020). Mechanotransducers convert shear forces into intracellular signals that affect endothelial barrier function (Tzima *et al*, 2005; Li *et al*, 2014; Conway *et al*, 2017; Albarran‐Juarez *et al*, 2018; Mehta *et al*, 2020). Some of the pathways that mediate shear stress effects have been elucidated (Conway *et al*, 2017; Albarran‐Juarez *et al*, 2018), but much is still to be learned about how endothelial cells distinguish atheroprotective from atherogenic shear forces. VE‐PTP had not previously been implicated.

The present study sought to determine the contribution of VE‐PTP and Tie2 activation to effects of shear stress on endothelial barrier function and atherogenesis. The approach was to compare VE‐PTP redistribution, Tie2 phosphorylation, and macromolecular leakage in regions of mouse aorta known to have contrasting shear stress profiles and in cultured endothelial cells exposed to defined flow conditions. Similar approaches were used to determine the contribution of VE‐PTP to leakage and atheroma formation in newly created mice having genetic ablation of VE‐PTP in endothelial cells, alone or in combination with apolipoprotein E gene deletion (ApoE^−/−^) and a high‐fat diet.

The experiments revealed that VE‐PTP in endothelial cells in regions of the aorta exposed to atheroprotective flow conditions was redistributed to the downstream pole of the cells and was internalized. These regions also had strong Tie2 phosphorylation at intercellular junctions, limited leakage, and less atheroma formation. By comparison, aortic endothelial cells exposed to disturbed flow had diffuse surface VE‐PTP with less internalization, lower Tie2 activation, impaired endothelial barrier function, and more atherogenesis in ApoE^−/−^ mice on a high‐fat diet. VE‐PTP gene deletion or pharmacological inhibition reduced leakage and atheroma formation in these mice. Together, the findings provide evidence that VE‐PTP inhibition can reduce atheroma formation. Increased Tie2 signaling resulting from reduced VE‐PTP phosphatase activity in endothelial cells has the beneficial consequences of tightening endothelial barrier function, reducing leakage, and protecting against atherogenesis.

## Results

### Shear stress‐driven VE‐PTP redistribution, Tie2 activation, and leakage protection

**Figure 1:**
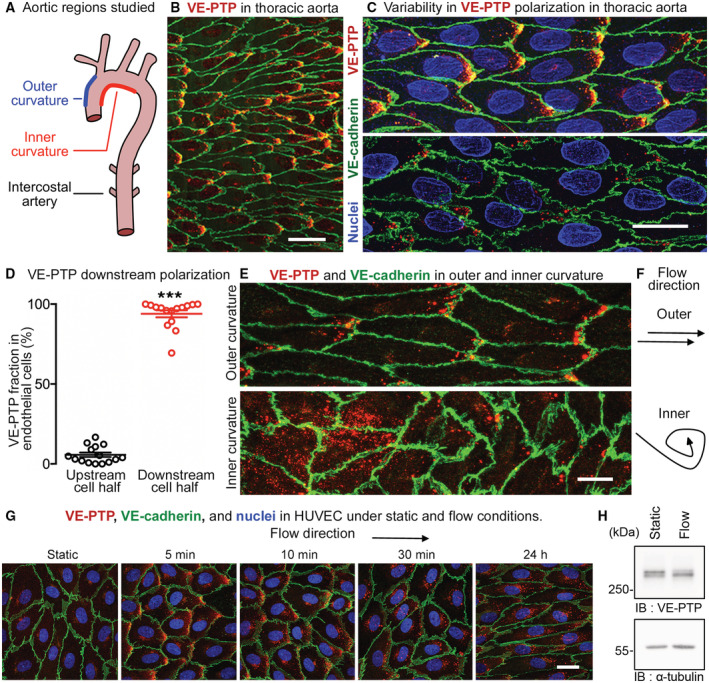
Downstream polarization of VE‐PTP in endothelial cells Regions of mouse aortic arch and thoracic aorta studied.Downstream polarization of VE‐PTP (red) in endothelial cells in the thoracic aorta. Blood flow: left to right. VE‐cadherin, green.Higher magnification views of a region similar to (B), showing variability of VE‐PTP polarization ranging from conspicuous (upper) to subtle (lower). Nuclei, blue (lamin A/C).Dot plot of proportions of VE‐PTP staining in upstream and downstream halves of endothelial cells in thoracic aorta of wild‐type mouse. Each dot is one endothelial cell. Mean ± SEM, *n* = 15 cells.VE‐PTP staining (red) in aortic arch endothelial cells contrasts distinct polarization in outer curvature and diffuse staining in inner curvature. Endothelial cells are elongated in outer curvature, but polygonal in inner curvature with jagged cell borders. VE‐PTP, red. VE‐cadherin, green.Contrasting directions of blood flow in the two regions.VE‐PTP staining (red) in HUVEC under static conditions and after laminar flow (15 dyn/cm^2^; 5 min – 24 h). Nuclei, blue (Hoechst 33342). Faint diffuse VE‐PTP staining in the static control contrasts with downstream polarization after flow and change from diffuse downstream staining at 5 min to dot‐like staining at 30 min. Intracellular VE‐PTP is more dispersed at 24 h. See related images in Appendix Fig S2.HUVEC were grown under static conditions or for 24 h under 15 dyn/cm^2^ flow, and then cell lysates were immunoblotted (IB) for VE‐PTP and tubulin. Data information: ***P < 1 × 10^−15^, by Student's *t*‐test in (D). Scale bars: 40 μm in (B), 20 μm in (C and G), 10 μm in (E). Source data are available online for this figure.

The experiments took advantage of the well‐documented regional differences in shear stress‐related permeability of the mouse aorta to elucidate links between VE‐PTP, Tie2 phosphotyrosine 992 (Tie2‐pY992), and endothelial barrier function (Fig 1A). Regions of the outer (greater) curvature of the mouse aortic arch, away from branches, are exposed to more uniform flow and higher average longitudinal wall shear stress than the inner (lesser) curvature, where flow is disturbed and oscillatory and average shear stress is lower (Suo *et al*, 2007). As a result of these differences, the endothelium of the inner curvature has more leakage and atheroma formation than the outer curvature (Xiao *et al*, 1998; McGillicuddy *et al*, 2001; Bond *et al*, 2010). The endothelium around ostia of intercostal arteries is exposed to variable shear stress, depending on the location relative to the flow divider and opening. These regional differences have proven useful for side‐by‐side comparison of endothelial cell barrier function and atheroma formation (Xiao *et al*, 1998; McGillicuddy *et al*, 2001; Suo *et al*, 2007; Bond *et al*, 2010).

#### VE‐PTP polarization

Endothelial cells in the thoracic aorta had heterogeneous staining for VE‐PTP. Some endothelial cells had conspicuous regions of dot‐like VE‐PTP staining near the downstream cell pole (Fig 1B and C, upper). In others, VE‐PTP staining in endothelial cells varied from polarized but sparse to dispersed or nearly absent (Fig 1C, lower). Measurements revealed an average of 94% of VE‐PTP staining in the downstream half of the cells (Fig 1D, Appendix Fig S1A and B).

Specificity of the polarized staining for VE‐PTP was confirmed by examining VE‐PTP^iECKO^ mice (Frye *et al*, 2015), which had little or no VE‐PTP in endothelial cells as verified by immunoblot (Appendix Fig S1C) and immunohistochemical staining (Appendix Fig S1D–F). The specificity of VE‐PTP staining was also confirmed by comparing human umbilical vein endothelial cells (HUVEC), which had clear VE‐PTP staining after control siRNA but little or none after exposure to VE‐PTP siRNA (Appendix Fig S1G and H).

The relationship of downstream polarization of VE‐PTP staining in endothelial cells to the blood flow pattern was determined by comparing staining in the aortic arch outer curvature, which has higher average shear, to staining in the inner curvature, where flow is disturbed and average shear is lower. The distribution of VE‐PTP staining was clearly different in the two regions: staining had dot‐like polarization toward the downstream tip of endothelial cells in the outer curvature, but was dispersed in the inner curvature (Fig 1E and F).

The causal link between shear stress and VE‐PTP distribution was directly examined *in vitro* by comparing HUVEC exposed to static conditions or unidirectional laminar shear stress of 15 dyn/cm^2^ for 5 min to 24 h. Under static conditions, faint VE‐PTP staining in HUVEC was located at the cell border and near the nucleus without evidence of polarization, but after 5 min of laminar flow, VE‐PTP was distinctly polarized downstream (Fig 1G, Appendix Fig S2). At 10 and 30 min, VE‐PTP staining had a polarized dot‐like pattern (Fig 1G). At 24 h, the dot‐like pattern of VE‐PTP staining was broadly distributed, unlike the polarized pattern in the aorta, but the elongated shape of HUVEC was similar to that of aortic endothelial cells (Fig 1G, Appendix Fig S2). Unlike pulsatile flow and other flow dynamics in the aorta, sustained laminar flow *in vitro* is likely to contribute to this feature of VE‐PTP in HUVEC at 24 h. No change in the overall expression level of VE‐PTP was found after 24 h of flow, as determined by immunoblotting (Fig 1H).

To investigate potential mechanisms which could be responsible for the flow‐induced redistribution of VE‐PTP, we analyzed the relevance of VEGFR2, known to be involved in flow sensing (Tzima *et al*, 2005) and RhoA, known to be involved in cell alignment (Tzima *et al*, 2001; Wojciak‐Stothard & Ridley, 2003). Neither blocking VEGFR2 with the kinase inhibitor SU‐1498 nor silencing of RhoA by siRNA blocked flow induced redistribution of VE‐PTP in HUVEC (Appendix Fig S3A–C).

Together, the data provide evidence that VE‐PTP has a dot‐like appearance and downstream polarization in regions of aorta and HUVEC exposed to high average shear stress but not in regions of low shear stress accompanying disturbed, oscillatory, or no flow.

#### VE‐PTP internalization

**Figure 2:**
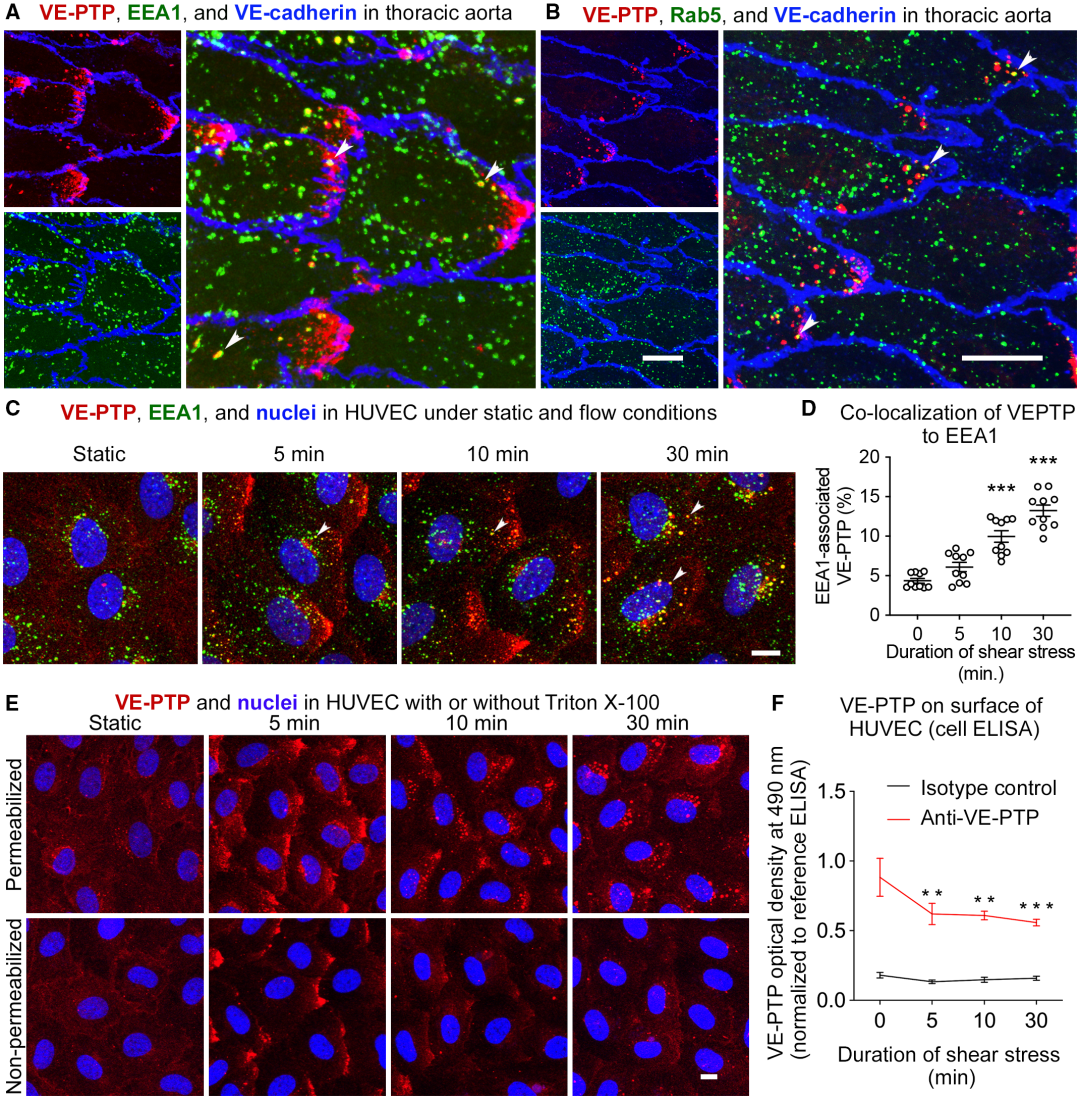
VE‐PTP internalization into endosomes under flow conditions Confocal microscopic images of VE‐PTP (red) and early endosome marker EEA1 (green) showing colocalization in some endosomes near downstream tip of endothelial cells (arrowheads) in thoracic aorta of wild‐type mouse. Blood flow left to right. Patches of VE‐PTP and many scattered endosomes without colocalization are also present.Similar field showing VE‐PTP and early endosome marker Rab5 (green) with colocalization in some dot‐like endosomes (arrowheads).VE‐PTP internalization in HUVEC into EEA1 endosomes (arrowheads) under static conditions or laminar flow of 15 dyn/cm^2^ of shear force for 5, 10, and 30 min.Quantification of VE‐PTP staining that colocalizes with EEA1 in endothelial cells in (C). Mean ± SEM, *n* = 10 cultures on independent flow chamber lanes.HUVEC exposed to same conditions as in (C), followed by fixation with (upper row) or without (lower row) permeabilization with 0.3% Triton X‐100, and staining for VE‐PTP (red) and nuclei (blue, Hoechst 33342).VE‐PTP on surface of HUVEC measured by cell‐based ELISA assay of HUVEC after exposure as in (C) and (D). Red and black lines show measurements with anti‐VE‐PTP antibody and isotype control antibody. Mean ± SEM, *n* = 6 experiments with independently cultured cells. Data information: **P < 0.01, ***P < 0.001 by one‐way ANOVA followed by Dunnett's test in (D) and (F). *P* < 1 × 10^−15^ (0 min vs. 10 min and 30 min) in (D). *P* = 0.0080 (0 min vs. 5 min) and 0.0056 (0 min vs. 10 min) and 0.0010 (0 min vs. 30 min) in (F). Scale bars: 10 μm in (A–C, E).

The distinctive dot‐like pattern of VE‐PTP staining at the downstream tip of aortic endothelial cells led us to determine whether VE‐PTP was internalized into endosomes in these locations. Some dot‐like VE‐PTP‐staining colocalized with early endosome markers EEA1 or Rab5 (Fig 2A and B) at these sites. EEA1‐ and Rab5‐positive endosomes elsewhere in the cells had no VE‐PTP staining. In addition to VE‐PTP in endosomes, some uniformly intense VE‐PTP staining at the downstream tip of endothelial cells was likely to be concentrated in the plasma membrane (Fig 2A and B).

To test the interpretation of VE‐PTP internalization into endosomes in aortic endothelial cells *in vivo*, we asked whether VE‐PTP was internalized into HUVEC exposed to high shear. Using the same approach that revealed VE‐PTP polarization in endothelial cells *in vitro*, HUVEC were exposed to shear stress of 15 dyn/cm^2^ for 0 (static), 5, 10, or 30 min and then fixed and stained for VE‐PTP and EEA1. Although little or no VE‐PTP was colocalized with EEA1 at 0 or 5 min, colocalization was significantly increased in endosomes at 10 and 30 min (Fig 2C and D). Large patches of VE‐PTP at the downstream tip of HUVEC at 5 or 10 min that lacked EEA1 staining are likely to be located in the plasma membrane, as judged from staining of permeabilized and non‐permeabilized cells (see below).

To examine further the cellular location of staining by antibodies to VE‐PTP extracellular domain (Baumer *et al*, 2006; Li *et al*, 2020), similar experiments were performed with HUVEC that were then fixed with or without permeabilization by Triton X‐100. Under static conditions, VE‐PTP staining of HUVEC was faint at cell borders and was diffusely distributed over the entire cell surface with or without permeabilization (Fig 2E). Faint VE‐PTP staining was also found in the perinuclear Golgi region of permeabilized cells (Fig 2E). In contrast, VE‐PTP staining in HUVEC exposed to laminar flow for 5 min was concentrated at the downstream cell tip of both permeabilized and unpermeabilized cells, whereas at 10 and 30 min, VE‐PTP was in dot‐like vesicles in the downstream half of permeabilized cells but not non‐permeabilized cells (Fig 2E).

Consistent with shear stress‐induced internalization, VE‐PTP surface expression, detected in HUVEC by cell‐based ELISA assay, was reduced by exposure to 15 dyn/cm^2^ shear force for 30 min (Fig 2F). Taken together, these findings are evidence that laminar shear stress promotes VE‐PTP redistribution and then removal from the cell surface by internalization into endosomes.

**Figure EV1:**
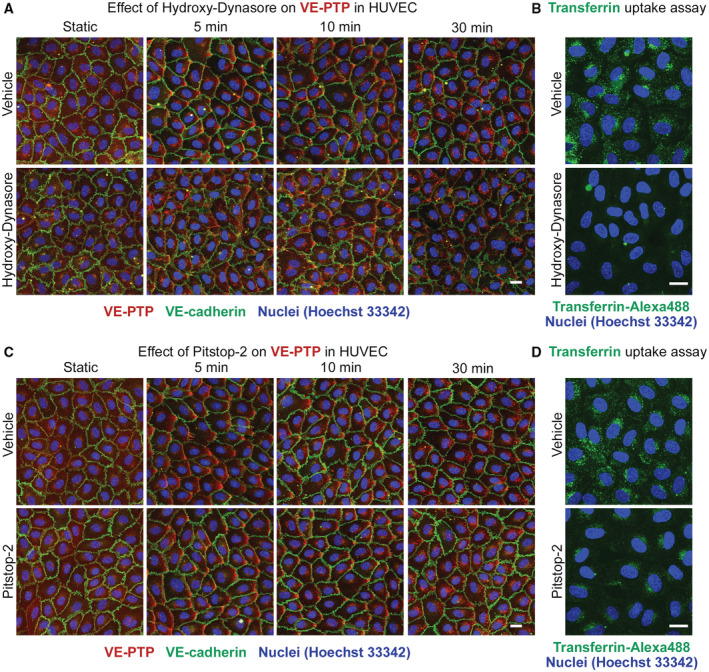
Dynamin and Clathrin inhibitors do not affect internalization of VE‐PTP under shear stress AHUVECs were treated either with DMSO (vehicle) or with Hydroxy‐Dynasore at 100 μM for 1 h followed by exposure to shear stress of 15 dyn/cm^2^ for 5 to 30 min. Subsequently, cells were fixed, permeabilized, and stained for VE‐PTP (red), VE‐cadherin (green) and for nuclei (blue).BHUVECs were treated with vehicle or Hydroxy‐Dynasore as in (A), followed by incubation under static conditions with transferrin‐Alexa 488 (green) at 20 μg/ml for 15 min. Non‐internalized transferrin was removed with acetic buffer pH 2.0.C, DSimilar as (A and B), except that Hydroxy‐Dynasore was replaced by Pitstop‐2 at 30 μM. Data information: Scale bars: 20 μm.

Investigating the endocytosis mechanism, we tested whether inhibitors of dynamin (Dynasore) or clathrin (Pitstop‐2) would block flow induced endocytosis of VE‐PTP. Unexpectedly, we did not see any inhibitory effect (Fig EV1), suggesting that VE‐PTP is endocytosed by non‐classical mechanisms (Maldonado‐Báez *et al*, 2013; Mayor *et al*, 2014).

#### Tie2 activation

**Figure 3:**
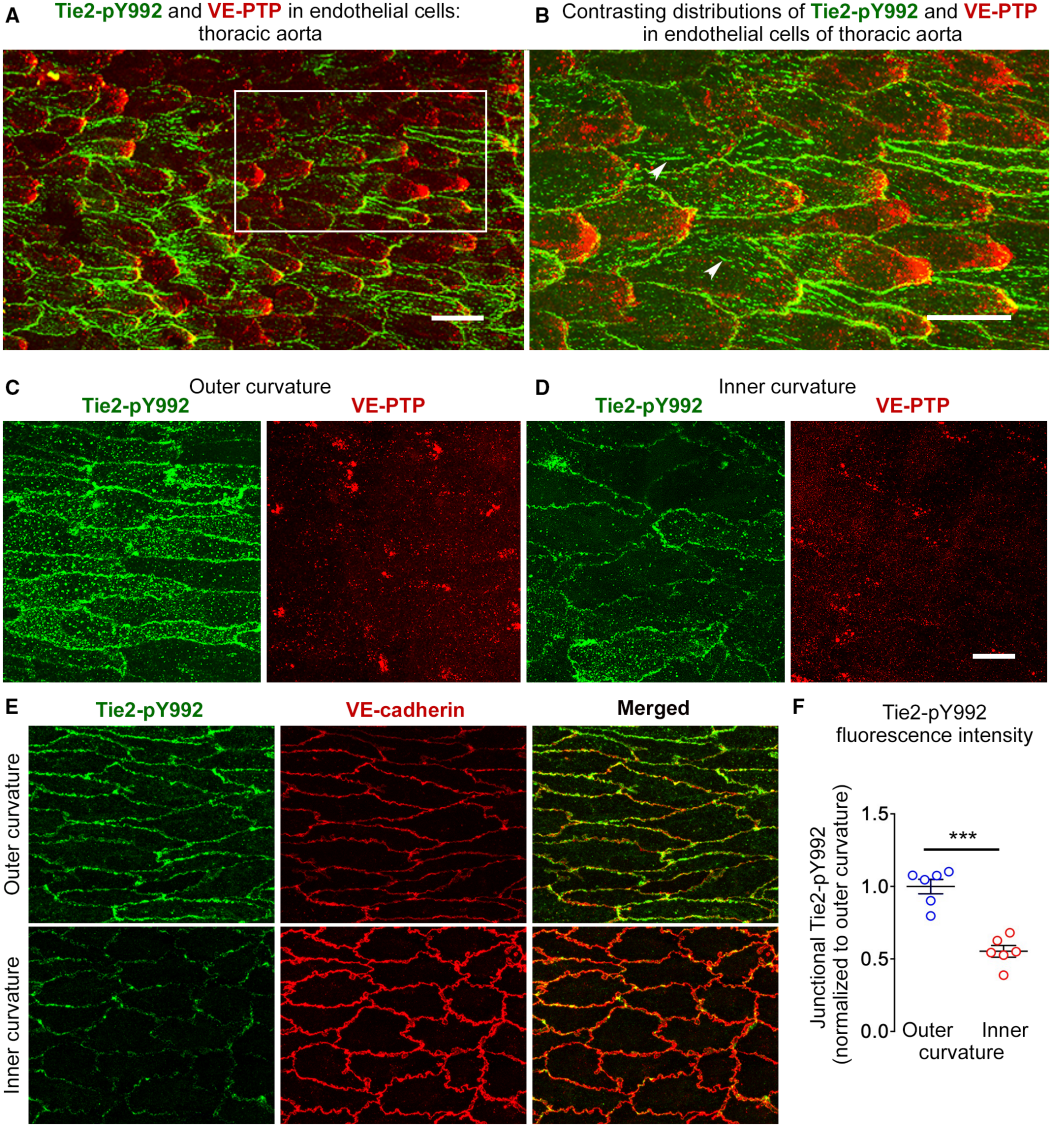
Tie2 phosphorylation, VE‐PTP, and VE‐cadherin distribution in endothelial cells of aorta AStrong staining for Tie2‐pY992 (green) and polarized VE‐PTP (red) in endothelial cells of thoracic aorta of wild‐type mouse.BEnlargement of region marked by white box in (A), comparing the distributions of Tie2‐pY992 at endothelial cell borders and focal adhesions (arrowheads) with VE‐PTP polarized to the downstream cell tip of endothelial cells.C, DStrong Tie2‐pY992 (green) staining at cell borders and VE‐PTP (red) downstream polarization in outer curvature in (C) in contrast to weak, dispersed staining for Tie2‐pY992 and VE‐PTP in inner curvature in (D) of aortic arch of wild‐type mouse.E, FTie2‐pY992 (green) at endothelial junctions marked by VE‐cadherin (red). (E) Contrasting patterns and intensity of Tie2‐pY992 in outer and inner curvatures. (F) Tie2‐pY992 mean fluorescence intensity at junctions (normalized to outer curvature = 1.0). Mean ± SEM, *n* = 6 mice/group. Data information: ***P = 0.000049, by Welch's *t*‐test in (F). Scale bars: 25 μm in (A), 20 μm in (B–E).

Because of the importance of Tie2 as a substrate of VE‐PTP phosphatase activity, we asked whether polarization of VE‐PTP in endothelial cells was accompanied by changes in Tie2 activation. By using previously validated antibodies to compare Tie2‐pY992 (Kim *et al*, 2016) and VE‐PTP (Baumer *et al*, 2006) immunoreactivities in aortic regions exposed to different flow conditions, we found strong Tie2‐pY992 staining at cell borders together with downstream polarization of VE‐PTP in some endothelial cells in the thoracic aorta (Fig 3A and B).

**Figure EV2:**
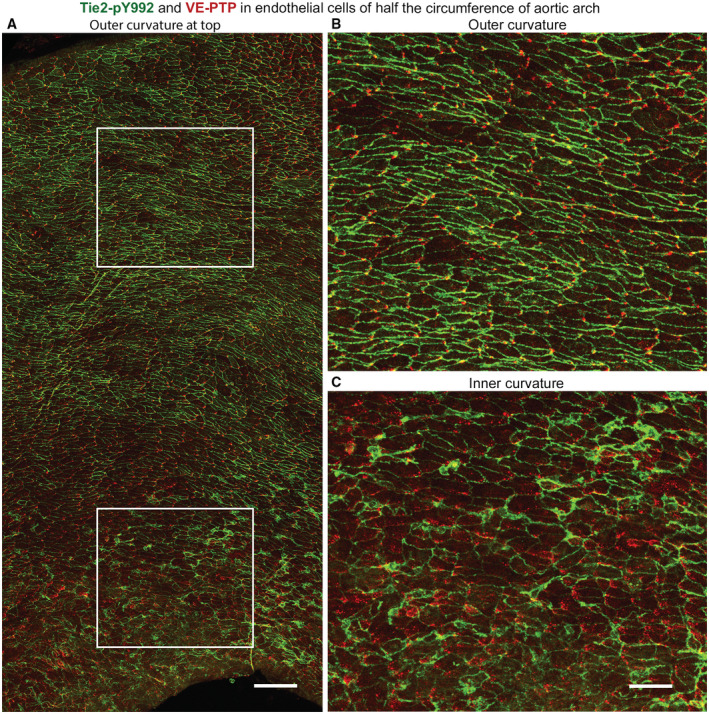
Regional differences in VE‐PTP and Tie2‐pY992 staining in aortic arch ALow magnification confocal microscopic tile‐scan image of half the circumference of bisected aortic arch of normal wild‐type mouse. Outer curvature is at the top and inner curvature is at the bottom. VE‐PTP (red) and Tie2‐pY992 (green).B, CRegions in white boxes in (A) are enlarged to show striking differences in VE‐PTP and Tie2‐pY992 staining in the outer curvature in (B) and inner curvature in (C). Regional heterogeneity in VE‐PTP and Tie2‐pY992 staining is also present within the outer curvature and even more so within the inner curvature. Net direction of blood flow is left to right. Data information: Scale bars: 100 μm in (A), 50 μm in (B and C).

Like regions of thoracic aorta, Tie2‐pY992 staining in the outer curvature of the aortic arch was strongest at intercellular junctions and VE‐PTP was polarized downstream (Fig 3C). In the inner curvature, however, staining for Tie2‐pY992 was weaker and VE‐PTP was more dispersed and had little or no polarization (Fig 3D). Measurements revealed stronger Tie2‐pY992 staining at junctions in the outer curvature than inner curvature (Fig 3E and F). Tile‐scan confocal imaging of large regions documented the gradient of decreasing Tie2‐pY992 staining and VE‐PTP polarization from outer to inner curvature (Fig EV2A–C).

These findings are consistent with stronger Tie2 phosphorylation at endothelial junctions in aortic regions where high average shear stress promotes VE‐PTP redistribution to the downstream cell pole and internalization from the plasma membrane.

**Figure 4:**
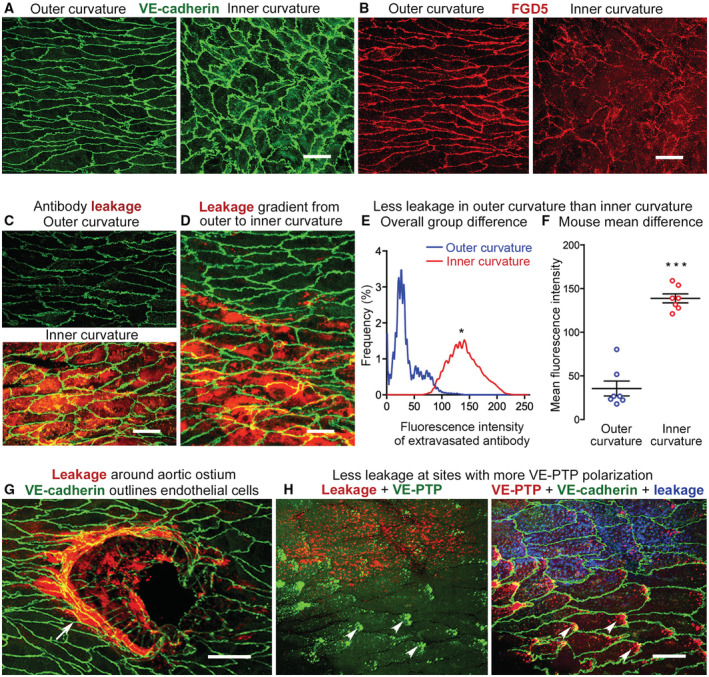
Endothelial cell junctions and plasma leakage in the aorta VE‐cadherin (green) of adherens junctions is continuous at endothelial cell borders in both the outer and inner curvature, but junctions in inner curvature are jagged and have tiny finger‐like processes, and endothelial cell shape is markedly different.FGD5, a GTPase exchange factor required for the anti‐leakage effect of Tie2 activation, is continuous in outer curvature, but is discontinuous in the inner curvature.Little leakage of anti‐fibrinogen antibody (red) in outer curvature compared with extensive leakage in inner curvature. VE‐cadherin (green).Gradient of anti‐fibrinogen antibody leakage (red) at interface of outer (upper) and inner curvatures (lower). VE‐cadherin (green).Comparison of distribution of fluorescence intensities of extravasated antibody showing significantly stronger fluorescence due to greater leakage in inner curvature.Mean fluorescence intensity value for each mouse in the two groups shown in (E). Mean ± SEM, *n* = 7 mice/group.Localized distribution of intense leakage (red) around ostium of an intercostal artery (arrow). VE‐cadherin (green).Opposite distributions of extravasation and VE‐PTP polarization in endothelial cells in thoracic aorta. Left and right panels are the same image with colors switched to highlight leakage (red) and polarized VE‐PTP (green, arrowheads) on the left, and polarized VE‐PTP (red, arrowheads), VE‐cadherin (green), and leakage (blue) on the right. Data information: *P = 0.0219, by Kolmogorov–Smirnov two‐sample test in (E) and ***P = 0.00000025 by Student's *t*‐test in (F). Scale bars: 20 μm in (A–D, G and H).

#### Endothelial junctions and barrier function

The central role of Tie2 in the regulation of endothelial barrier function led us to examine the relationship of shear stress effects on VE‐PTP and Tie2 to differences in endothelial cell junctions and plasma leakage. Staining of VE‐cadherin at adherens junctions revealed distinct differences in endothelial cell shape in the outer and inner curvatures of the aortic arch and around intercostal ostia, as reported previously (Davies, 1995; Gimbrone & Garcia‐Cardena, 2013). Endothelial cells in the outer curvature were elongated in the direction of flow (Fig 4A, left), as in much of the thoracic aorta, whereas endothelial cells in the inner curvature were rounded, polygonal, and had jagged borders (Fig 4A, right), which are typical of sites of leakage (Claesson‐Welsh *et al*, 2021). Staining for the GTPase exchange factor FGD5, which is required for endothelial barrier tightening by Tie2 activation through effects on the cortical cytoskeleton (Braun *et al*, 2019), was largely continuous at endothelial cell junctions of the outer curvature (Fig 4B, left), but was discontinuous and weaker in the inner curvature (Fig 4B, right).

**Figure EV3:**
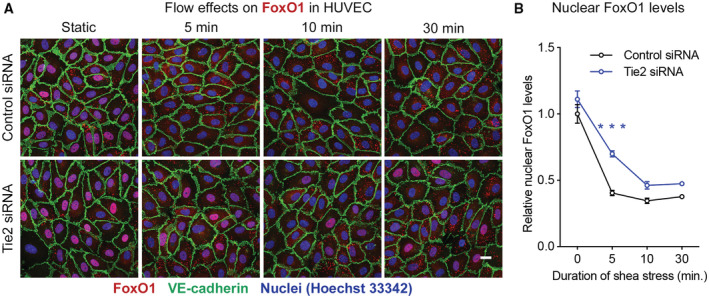
Tie2 is required for shear stress‐induced export of nuclear FoxO1 HUVECs treated either with control siRNA or Tie2 siRNA were exposed to 15 dyn/cm^2^ shear stress for 5 to 30 min, followed by fixation, permeabilization, and staining for FoxO1 (red), VE‐cadherin (green) and nuclei (blue). Scale bar: 20 μm.Amount of nuclear FoxO1 staining. Mean ± SEM, *n* = 6 cultures on independent flow chamber lanes. ***P < 0.001, by Bonferroni's test. *P* = 0.17 (0 min), 0.00000090 (5 min), 0.14 (10 min) and 0.31 (30 min).

In addition to an immediate supportive effect of junction tightening by Tie2‐induced activation of FGD5, indirect transcriptional effects could also contribute to the regulation of junctions. Inhibition of FoxO1 has been reported to modulate junction integrity (Taddei *et al*, 2008) and Tie2 signaling was shown to inhibit FoxO1 (Daly *et al*, 2004; Kim *et al*, 2016). We therefore tested whether shear stress would influence FoxO1 activity in HUVEC via Tie2. Indeed, we found that FoxO1 in nuclei was reduced within 5 min of the onset of flow, and this was attenuated when Tie2 was silenced by siRNA (Fig EV3).

Regional differences in endothelial barrier function in the aorta were assessed by using anti‐fibrinogen antibody injected intravenously (i.v.) as a tracer (Nakahara *et al*, 2006). Fibrinogen is known to accumulate in the aortic intima in regions of high leakage (Xiao *et al*, 1998; Reinhart, 2003). Little or no extravasated antibody was detected in the outer curvature at 30 min after i.v. injection, but the tracer was widespread in the inner curvature (Fig 4C). A distinct gradient in leakage was evident where the outer curvature met the inner curvature (Fig 4D). Measurements confirmed the clear difference in leakage in the two regions (Fig 4E and F). Use of anti‐fibrinogen antibody as a leakage tracer was validated by comparison to extravasation of normal rabbit IgG, which was evident in the inner curvature, but the signal in wild‐type mice was much less than for anti‐fibrinogen antibody (Appendix Fig S4A–C). Because both reagents were similar in size, the difference was more likely due to retention than amount of extravasation (Nakahara *et al*, 2006). Extravasated fluorescent microspheres (diameter 20‐nm) were also detected in the inner curvature at 30 min but not in the outer curvature (Appendix Fig S4D and E).

In the thoracic aorta, anti‐fibrinogen antibody extravasated around the perimeter of intercostal ostia (Fig 4G), where extravasation was greater near endothelial cells with little VE‐PTP polarization than where polarization was prominent (Fig 4H). These findings provide evidence that polygonal shape, jagged adherens junctions, discontinuous FGD5, and little or no VE‐PTP polarization are features of endothelial cells in leaky regions of the aortic arch and thoracic aorta.

### Shear stress induced activation of Tie2 depends on VE‐PTP

**Figure 5:**
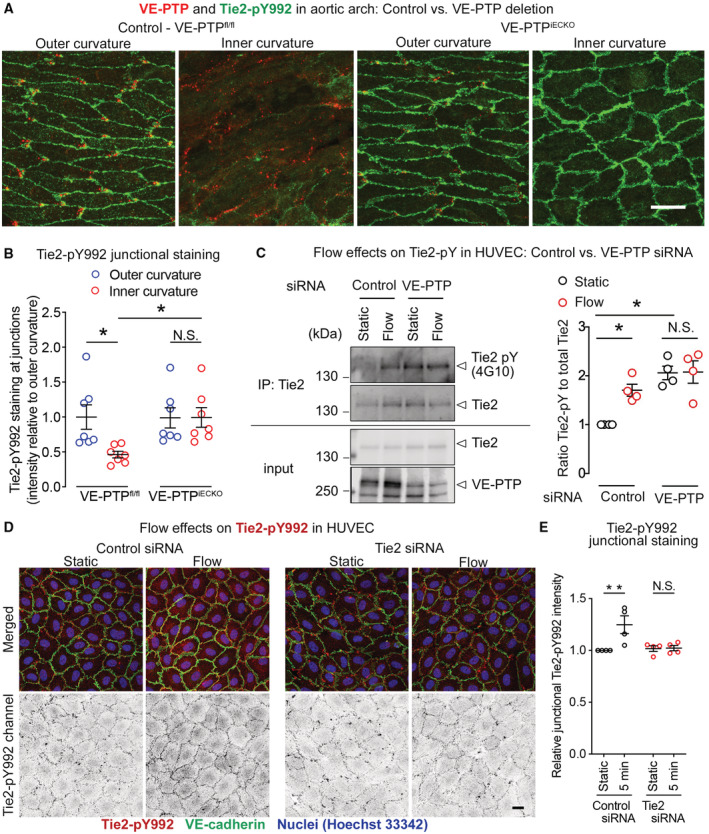
Shear stress promotion of VE‐PTP‐dependent Tie2 activation Polarized staining for VE‐PTP (red) and strong Tie2‐pY992 (green) in the aortic outer curvature but not inner curvature of VE‐PTP^fl/fl^ mice compared with the absence of VE‐PTP and equally strong Tie2‐pY992 staining in both curvatures of VE‐PTP^iECKO^ mice. Blood flow left to right.Measurements of Tie2‐pY992 staining intensity at intercellular junctions in aortic arch endothelial cells showing lower values in the inner curvature than in the upper curvature of VE‐PTP^fl/fl^ mice but equally high values in both regions of VE‐PTP^iECKO^ mice. Normalized to mean value for outer curvature of VE‐PTP^fl/fl^ mice scaled to 1.0. Mean ± SEM, *n* = 6 mice/group.HUVEC transfected with control siRNA or VE‐PTP siRNA and exposed to static or laminar shear force at 15 dyn/cm^2^ for 30 min, followed by immunoprecipitation of Tie2. Left. Blot showing immunoprecipitates (top) or cell lysates (bottom) immunoblotted for pan‐phosphotyrosine (antibody 4G10), Tie2, or VE‐PTP. Ratio of staining for phosphorylated Tie2 and total Tie2 (Tie2‐pY to Tie2, *n* = 4 cultures on independent fow chamber lanes). Right. Relative Tie2‐pY levels in HUVEC after control siRNA or VE‐PTP siRNA. For each blot, the measurement of the static control lane was normalized to 1.0 and corresponding values for the other three lanes were scaled accordingly. Mean ± SEM, *n* = 4 replicates.Effect of shear stress on the phosphorylation levels of Tie2 Y992 at cellular junctions. HUVECs treated with either control siRNA (left) or Tie2 siRNA (right), were exposed to 15 dyn/cm^2^ shear stress for 5 min. The resulting cells were fixed and stained for Tie2‐pY992 (red) and VE‐cadherin (green).In each experiment, the amount of junctional Tie2‐pY992 immunoreactivity in the static control was normalized to 1.0 and corresponding values for the other conditions were scaled accordingly. Mean ± SEM, *n* = 4 replicates. Data information: *P < 0.05, **P < 0.01, by two‐way ANOVA followed by Tukey test in (B, C and E). N.S. not significant. *P* = 0.0455 (outer curvature, VE‐PTP^fl/fl^ vs, inner curvature, VE‐PTP^fl/fl^), 0.048 (inner curvature, VE‐PTP^fl/fl^ vs. inner curvature, VE‐PTP^iECKO^) and 0.99 (outer curvature, VE‐PTP^iECKO^ vs. inner curvature, VE‐PTP^iECKO^) in (B). *P* = 0.023 (Static, Control siRNA vs. Flow, Control siRNA), 0,013 (Static, Control siRNA vs. Static, VE‐PTP siRNA) and 0.99 (Static, VE‐PTP siRNA vs. Flow, VE‐PTP siRNA) in (C). *P* = 0.0058 (Control siRNA) and 0.99 (Tie2 siRNA) in (E). Scale bar: 20 μm in (A and D). Source data are available online for this figure.

We next determined whether shear stress‐driven VE‐PTP internalization could explain the differences in Tie2 activation in aortic arch endothelial cells. Specifically, we asked whether lower Tie2 activation in the inner curvature of the aortic arch, where flow is disturbed and average shear force is low, is dependent on higher VE‐PTP phosphatase activity due to less redistribution. The approach was to compare Tie2‐pY992 staining in the endothelium of the inner and outer curvatures of mice with inducible, endothelial cell‐specific VE‐PTP deletion (VE‐PTP^iECKO^ mice) and in corresponding VE‐PTP^fl/fl^ controls. Tie2‐pY992 staining at endothelial junctions was significantly greater in the outer curvature than inner curvature of VE‐PTP^fl/fl^ controls but not in VE‐PTP^iECKO^ mice, where Tie2‐pY992 was equally strong at endothelial junctions in both locations (Fig 5A and B).

Corresponding *in vitro* studies using HUVEC extended previous evidence of increased Tie2 phosphorylation after antibody‐induced endocytosis of VE‐PTP (Winderlich *et al*, 2009). Control HUVEC exposed to laminar shear stress of 15 dyn/cm^2^ for 30 min had significantly greater Tie2 tyrosine phosphorylation than in HUVEC under static conditions as was shown by immunoblotting for Tie2‐pY after immunoprecipitation of Tie2 (Fig 5C). The flow‐induced increase in Tie2‐pY did not occur after VE‐PTP silencing by siRNA (Fig 5C). Based on immunofluorescence staining, we observed an increase for the Tie2‐pY992 signal at junctions of HUVEC upon 5 min exposure to flow (Fig 5D, left) which was completely blocked upon silencing of Tie2 by siRNA (Fig 5D right). Quantification is shown in Fig 5E. In contrast to our *in vivo* analysis, the antibodies showed unspecific background staining in HUVEC under static conditions, which was not reduced by Tie2 siRNA and did not increase by flow. *In vivo*, Tie2‐pY992 signals in the aortic outer curvature were eliminated upon induced gene inactivation in Tie2^iECKO^ mice (Appendix Fig S5). The difference between the *in vitro* and *in vivo* staining specificity is partly due to the fact that the Tie2‐pY signals were much stronger *in vivo* than *in vitro*. Nevertheless, collectively our findings provide further evidence that laminar shear stress increases Tie2 phosphorylation at endothelial cell contacts by promoting VE‐PTP polarization and internalization.

### Reduction in leakage after VE‐PTP gene deletion in endothelial cells

**Figure 6:**
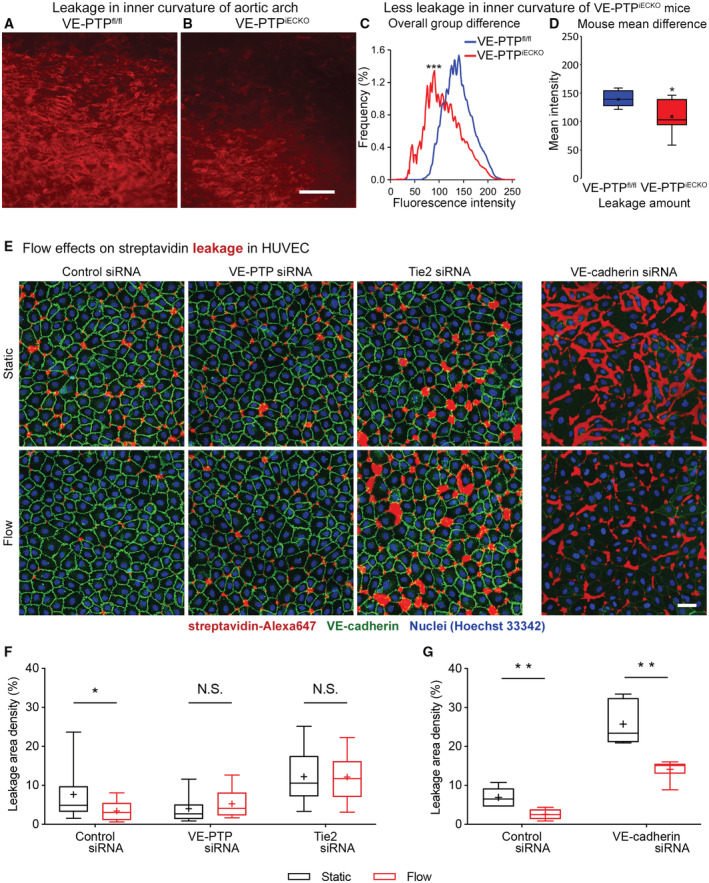
Relevance of VE‐PTP and Tie2 for flow‐induced endothelial junction tightening A, BRed fluorescence of extravasated anti‐fibrinogen antibody in aortic arch inner curvature of VE‐PTP^fl/fl^ mouse (control) and VE‐PTP^iECKO^ mouse at 30 min after i.v. injection of the antibody.C, DAmounts of extravasated antibody in the inner curvature reflected by the distribution of fluorescence intensities in (C) and box and whisker plots in (D), where boxes show 1^st^, 2^nd^ (median), and 3^rd^ quartiles and whiskers show maximum and minimum fluorescence intensity (x marks the mean). Both plots document significantly less leakage in the inner curvature of VE‐PTP^iECKO^ mice (*n* = 9) than of VE‐PTP^fl/fl^ mice (*n* = 7).EHUVECs cultured on biotinylated gelatin were treated either with control siRNA, VE‐PTP siRNA, Tie2 siRNA or VE‐cadherin siRNA, then exposed to 15 dyn/cm^2^ shear stress for 30 min followed by 3 min incubation with streptavidin‐Alexa647 (red), washing, fixation and staining for VE‐cadherin (green).F, GFractional area of streptavidin leakage per image. Mean ± SEM, *n* = 19 cultures on independent flow chamber lanes. (F), and *n* = 6 cultures on independent flow chamber lanes (G). Data information: *P < 0.05, **P < 0.01, ***P < 0.001, by Kolmogorov–Smirnov test in (C) (maximum difference in cumulative frequency curves = 39%), by Student's *t*‐test in (D), and by Mann–Whitney *U*‐test with Bonferroni correction in (F and G). *P* = 0.00051 in (C). *P* = 0.0214 in (D). *P* = 0.030 (Control siRNA), 0.17 (VE‐PTP siRNA) and 0.99 (Tie2 siRNA) in (F). *P* = 0.0043 (Control siRNA) and 0.0043 (VE‐cadherin siRNA) in (G). Scale bars: 100 μm in (A and B), 50 μm in (E).

The functional consequence of greater Tie2 activation in aortic endothelial cells exposed to high average shear force was assessed by comparing endothelial barrier function in the inner and outer curvatures of VE‐PTP^iECKO^ mice to VE‐PTP^fl/fl^ controls, using the approach described for wild‐type mice. The inner curvature of VE‐PTP^iECKO^ mice had significantly less extravasated anti‐fibrinogen antibody than of control VE‐PTP^fl/fl^ mice (Fig 6A–D).

**Figure EV4:**
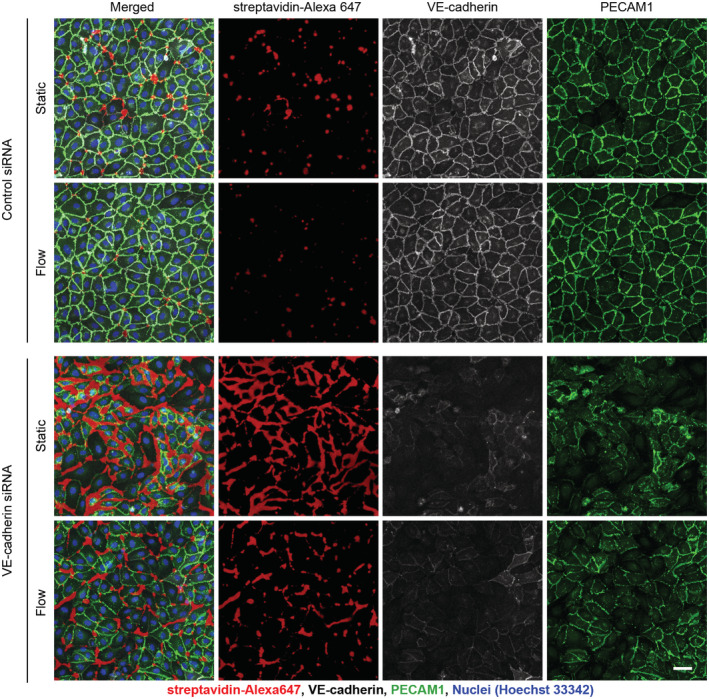
Flow‐inducedleakage reduction in HUVEC unchanged by VE‐cadherin siRNA HUVECs cultured on biotinylated gelatin were treated either with control siRNA, or VE‐cadherin siRNA, then exposed to 15 dyn/cm^2^ shear stress for 30 min followed by 3 min incubation with streptavidin‐Alexa647 (red), washing, fixation and staining for VE‐cadherin (gray) and PECAM1 (green). The VE‐cadherin siRNA data in Fig 6E are included in this figure. Scale bar: 50 μm.

To test whether flow‐induced protection against endothelial leakage is dependent on VE‐PTP modulated activity of Tie2, we established an *in vitro* assay that allowed us to visualize permeability across HUVEC monolayers with or without flow. To this end, cells were grown on biotinylated gelatin under flow conditions (15 dyn/cm^2^ for 30 min) or static conditions followed by incubation with directly labeled streptavidin for 3 min. We found that flow clearly reduced paracellular permeability to streptavidin (Figs 6E–G and EV4). This effect was lost when either VE‐PTP or Tie2 were silenced by siRNA (Fig 6E and F). In contrast, VE‐cadherin silencing by siRNA did not block the effect (Figs 6E and G, and EV4). Thus, VE‐PTP and Tie2, but not VE‐cadherin are essential for flow‐induced endothelial junction tightening.

**Figure EV5:**
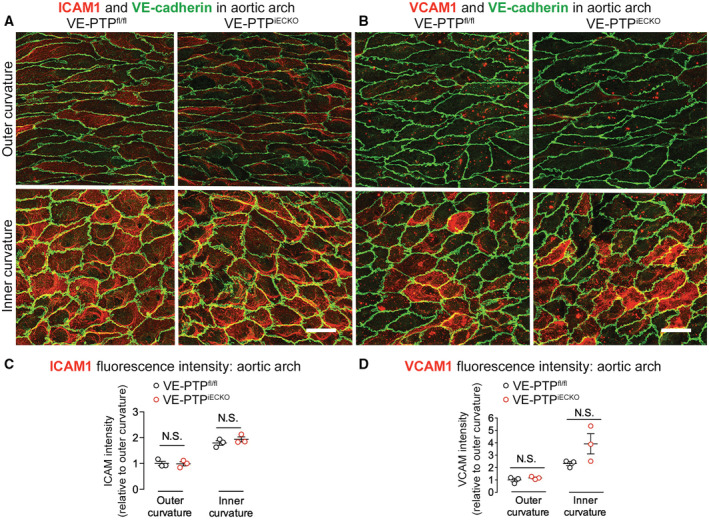
No change in ICAM1 or VCAM1 expression after VE‐PTP gene deletion A, BWeaker staining for ICAM1 in (A) and VCAM1 in (B) in outer curvature (top row) than in inner curvature (bottom row) of VE‐PTP^fl/fl^ mice and VE‐PTP^iECKO^ mice. Deletion of VE‐PTP had no apparent effect on ICAM1 or VCAM1 staining in either location. Scale bars: 20 μm.C, DMeasurements of mean fluorescence intensity confirmed stronger staining for ICAM1 and VCAM1 in the inner curvature but did not detect any significant difference between VE‐PTP^fl/fl^ and VE‐PTP^iECKO^ mice. Mean ± SEM, *n* = 3 mice/group. *P* values were determined with Bonferroni test in (C and D). N.S. not significant. *P* = 0.99 (outer curvature) and 0.60 (inner curvature) in (C). *P* = 0.99 (outer curvature) and 0.057 (inner curvature) in (D).

Because shear stress can also affect the expression of adhesion molecules involved in leukocyte migration (Iiyama *et al*, 1999; Suo *et al*, 2007), we asked whether VE‐PTP contributes to greater expression of intracellular adhesion molecule 1 (ICAM1) and vascular cell adhesion molecule 1 (VCAM1) in regions of low shear stress. The approach was to compare staining for ICAM1 and VCAM1 in the inner and outer curvatures of VE‐PTP^fl/fl^ mice and VE‐PTP^iECKO^ mice (Fig EV5A and B). Although ICAM1 and VCAM1 staining was greater in endothelial cells in the inner curvature, as shown previously (Iiyama *et al*, 1999; Suo *et al*, 2007), the amount of staining in VE‐PTP^iECKO^ mice was about the same as in VE‐PTP^fl/fl^ controls (Fig EV5C and D).

Together, these findings fit with the essential contribution of VE‐PTP to shear stress‐related regional differences in plasma leakage in the aortic arch. Deletion of VE‐PTP reduces leakage but has no apparent effect on VCAM1 and ICAM1 in low shear regions of the aortic arch.

### Reduction in atherogenesis after VE‐PTP gene deletion in endothelial cells

Extravasation of LDL‐cholesterol and other plasma components at sites of impaired endothelial barrier function in the aortic arch and branch points contributes to the formation of atherosclerotic plaques (Gimbrone & Garcia‐Cardena, 2013; Mundi *et al*, 2018). To determine the contribution of VE‐PTP to this process, we generated ApoE‐deficient mice in which VE‐PTP was deleted from endothelial cells (ApoE^−/−^/VE‐PTP^iECKO^ mice) and used ApoE‐deficient mice with intact VE‐PTP (ApoE^−/−^/VE‐PTP^fl/fl^ mice) as controls. Both groups were fed a high‐fat (Western‐type) diet to exaggerate hyperlipidemia. VE‐PTP gene inactivation in ApoE^−/−^/VE‐PTP^iECKO^ mice was confirmed by near absence of VE‐PTP immunofluorescence in aortic endothelial cells (Appendix Fig S6A) and of VE‐PTP bands in immunoblots of lung lysates (Appendix Fig S6B). Body weight and plasma cholesterol and triglyceride were essentially the same in the two strains of mice (Appendix Fig S6C and D).

**Figure 7:**
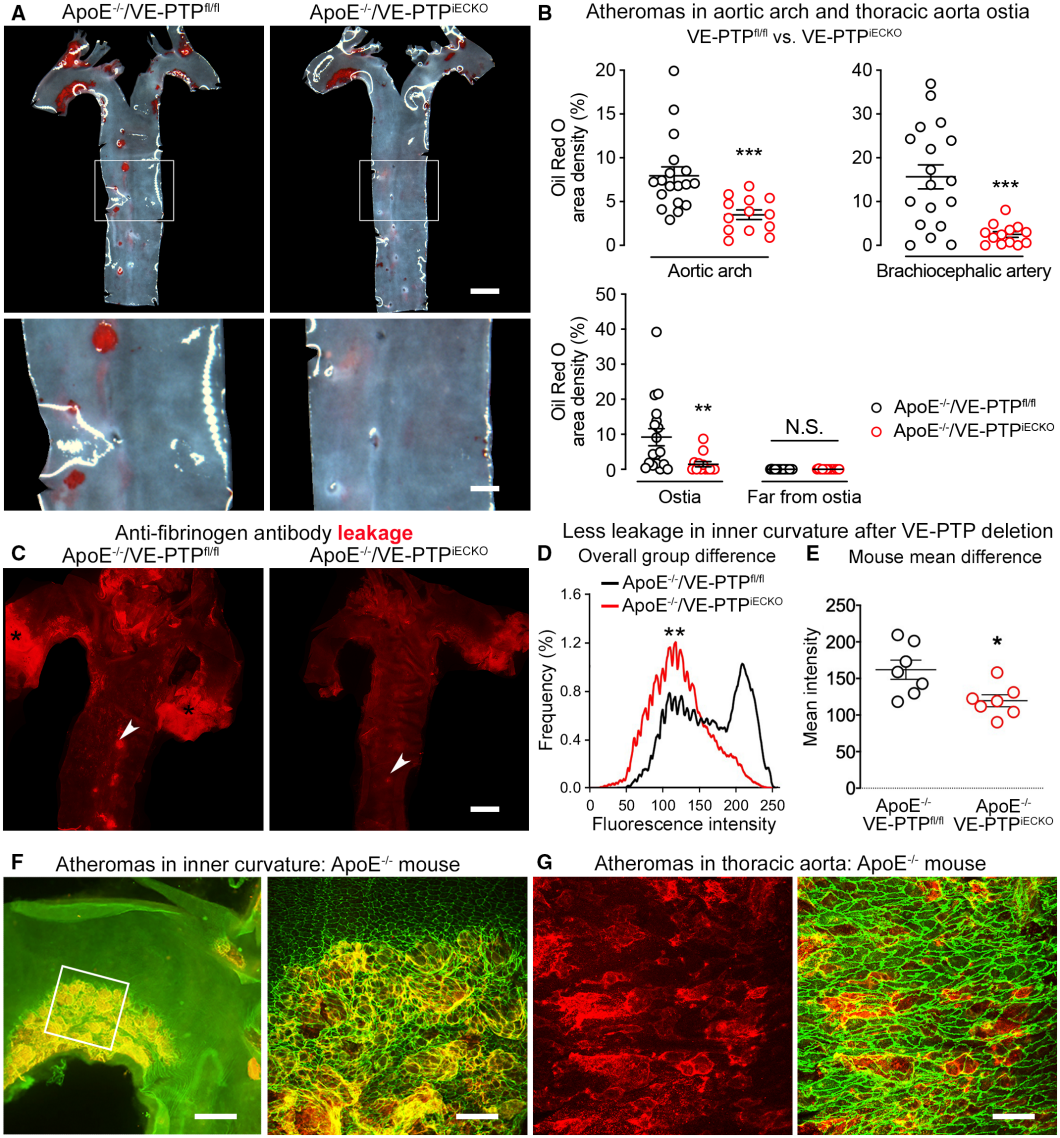
Reduction of atheromas and vascular leakage in ApoE^−/−^ mice with VE‐PTP gene deletion on high‐fat diet AOil Red O staining of atheromas in ApoE^−/−^/VE‐PTP^fl/fl^ mice and ApoE^−/−^/VE‐PTP^iECKO^ mice on high‐fat diet for 10 weeks.BFractional area occupied by Oil Red O‐stained atheromas in aortic arch, brachiocephalic artery, and around or away from intercostal artery ostia. Each dot is value for one mouse. Mean ± SEM, *n* = 13–18 mice/group.CSites of leakage revealed by extravasated anti‐fibrinogen antibody in aortas of ApoE^−/−^/VE‐PTP^fl/fl^ mouse (left) and ApoE^−/−^/VE‐PTP^iECKO^ mouse (right) on high‐fat diet for 7 weeks. Arrowheads mark intercostal artery ostia; black asterisks mark strong leakage at aortic root.D, EFluorescence intensity distributions in (D) and mean intensities in (E) of extravasated anti‐fibrinogen antibody in inner curvature of ApoE^−/−^/VE‐PTP^fl/fl^ mice and ApoE^−/−^/VE‐PTP^iECKO^ mice on high‐fat diet for 7 weeks. Mean ± SEM, *n* = 7 mice/group.F, GAtheromas in ApoE^−/−^ mice marked by extravasated anti‐fibrinogen antibody (red to yellow) in inner curvature at low magnification and enlarged (white box) in (F) and in thoracic aorta in (G). VE‐cadherin (green). Data information: *P < 0.05, **P < 0.01, ***P < 0.001, by two‐way ANOVA followed by Bonferroni test in (B), Kolmogorov–Smirnov test in (D), and Student's *t*‐test in (E). N.S. not significant. *P* = 0.00069 (aortic arch), 0.00017 (brachiocephalic artery), 0.0010 (around ostia in descending thoracic aorta), and 0.99 (far from ostia in descending thoracic aorta) in (B). *P* = 0.0010 in (D). *P* = 0.018 in (E). Scale bars: 1 mm in (A) upper row and (C), 300 μm in (A) lower row, 400 μm in (F) left, 100 μm in (F) right, 50 μm in (G).

Oil Red O staining of the aorta of ApoE^−/−^/VE‐PTP^fl/fl^ controls revealed widespread atherogenesis after 10 weeks on a high‐fat diet (Fig 7A). Atheromas were most abundant in the aortic arch and near ostia of intercostal arteries (Fig 7A), as reported previously in ApoE^−/−^ mice (Nakashima *et al*, 1994). In evidence of the contribution of VE‐PTP to atherogenesis, Oil Red O staining was 56% less in the aortic arch, 84% less in the brachiocephalic artery and 84% less at ostia of intercostal arteries in ApoE^−/−^/VE‐PTP^iECKO^ mice on a high‐fat diet for 10 weeks (Fig 7B). The reduction in plaque area in ApoE^−/−^/VE‐PTP^iECKO^ mice on high‐fat diet was confirmed by examining cryostat sections of the aortic root in the plane of the aortic valve stained with Oil Red O (Appendix Fig S7).

Consistent with the involvement of reduced Tie2 signaling in atherogenesis, Tie2‐pY992 staining was clearly less in endothelial cells overlying atheromas (Appendix Fig S8A and B). A causal link between suppression of Tie2 signaling and impaired endothelial barrier function was further indicated by significantly less extravasation of anti‐fibrinogen antibody in atheromas in the inner curvature of ApoE^−/−^/VE‐PTP^iECKO^ mice than in corresponding ApoE^−/−^/VE‐PTP^fl/fl^ controls (Fig 7C). Measurements confirmed that leakage in the inner curvature of ApoE^−/−^/VE‐PTP^iECKO^ mice was significantly less than in ApoE^−/−^/VE‐PTP^fl/fl^ controls on a high‐fat diet for 7 weeks (Fig 7D and E). Little leakage was found in the outer curvature in either mouse strain on a high‐fat diet. The distribution of extravasated tracer antibody reflected the extent and heterogeneous structure of atheromas in ApoE^−/−^/VE‐PTP^fl/fl^ controls on a high‐fat diet (Fig 7F and G).

This evidence indicates that VE‐PTP deletion reduced leakage and atheroma formation in aortic regions exposed to disturbed flow and low average shear stress. The atheroprotective effect was evident in mice genetically predisposed to atherogenesis amplified by a high‐fat diet.

### Reduction in atheromas after VE‐PTP inhibition by AKB‐9785

**Figure 8:**
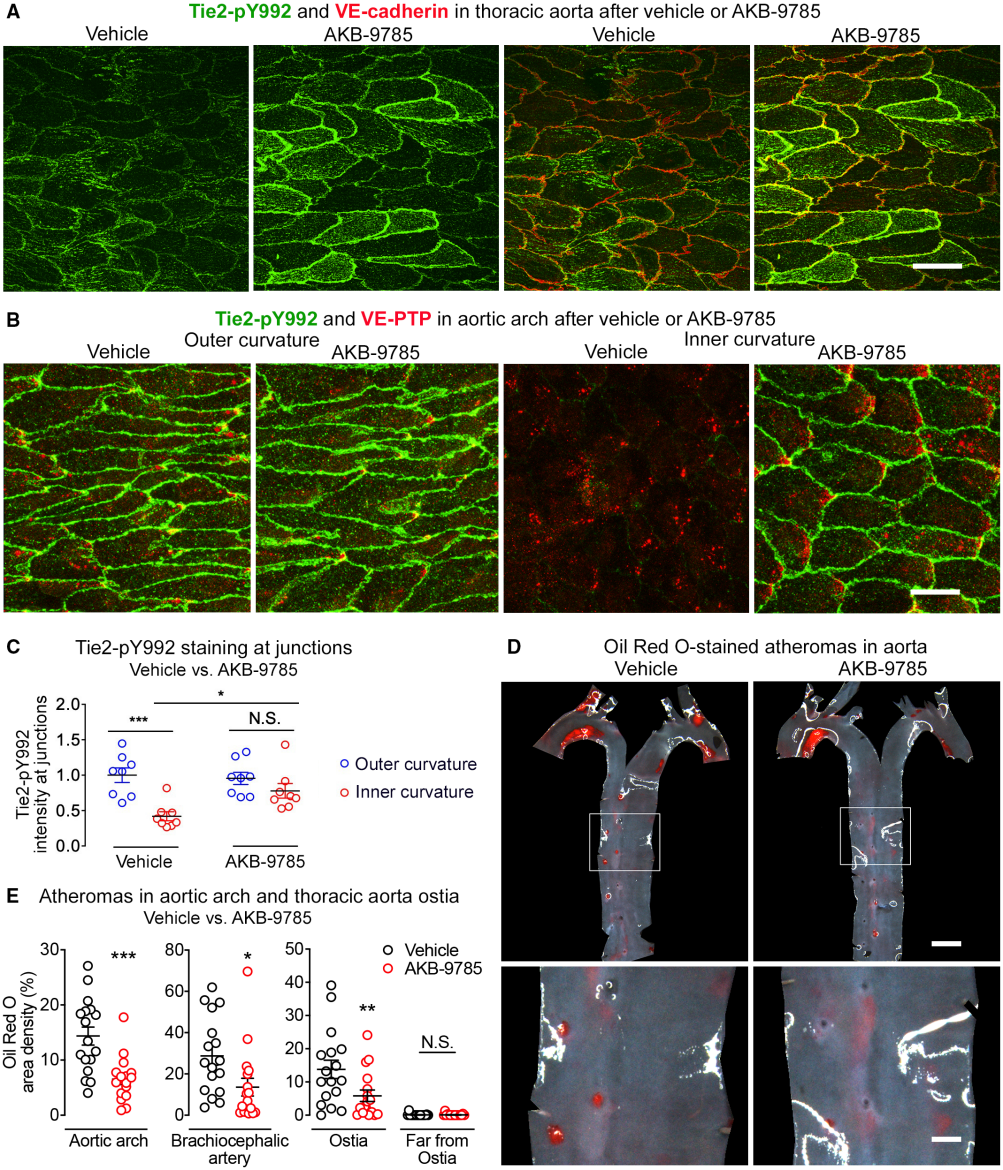
VE‐PTP inhibitor AKB‐9785 amplification of Tie2 phosphorylation and reduction in atherogenesis A, BTie2‐pY992 staining in aortic endothelial cells is shown with VE‐cadherin in (A) and with VE‐PTP in (B). Images in (A) show the thoracic aorta 1 h after the second of two doses of AKB‐9785 (6 h after first dose). Images in (B) show the aortic arch 1 h after a single dose of AKB‐9785. Tie2‐pY992 staining at cell borders is stronger after AKB‐9785 in the thoracic aorta and in the inner curvature but not outer curvature.CMeasurements of Tie2‐pY992 intensity at intercellular junctions in aortic arch of mice shown in (B). Mean ± SEM, *n* = 8 mice/group.DAtheromas stained with Oil Red O in aortas of ApoE^−/−^ mice treated with AKB‐9785 daily for 11 weeks while on high‐fat diet for the final 10 weeks. Regions of thoracic aorta outlined by white boxes are shown at higher magnification in bottom row.EFractional area occupied by Oil Red O‐stained atheromas in aortic arch, brachiocephalic artery, and around or away from intercostal artery ostia. Each dot is value for one mouse. Mean ± SEM, *n* = 17 mice/group. Data information: *P < 0.05, **P < 0.01, ***P < 0.001, by two‐way ANOVA followed by Tukey test in (C) and by Bonferroni test in (E). N.S. not significant. *P* = 0.0005 (outer curvature, vehicle vs. inner curvature, vehicle), 0.042 (inner curvature, vehicle vs. inner curvature, AKB‐9785) and 0.52 (outer curvature, AKB‐9785 vs. inner curvature, AKB‐9785) in (C). *P* = 0.00033 (aortic arch), 0.0216 (brachiocephalic artery), 0.0020 (around ostia in descending thoracic aorta) and 0.99 (far from ostia in descending thoracic aorta) in (E). Scale bars: 20 μm in (A and B), 1 mm in (D) upper row, 300 μm in (D) lower row.

To explore further the atheroprotective effect of VE‐PTP deletion, we asked whether pharmacologic inhibition of VE‐PTP with AKB‐9785 (Siragusa *et al*, 2021) increased Tie2 phosphorylation and reduced atherosclerosis. To test the action on Tie2 signaling in aortic endothelial cells, AKB‐9785 was administered and Tie2‐pY992 immunoreactivity was assessed in wild‐type mice. Tie2‐pY992 staining was stronger at endothelial cell borders in the thoracic aorta after AKB‐9785 than after vehicle (Fig 8A and Appendix Fig S9A–C). In addition to endothelial cell contacts, focal adhesion structures identified by paxillin staining showed a slight increase of Tie2‐pY992 staining after AKB‐9785 treatment (Appendix Fig S9C). Interestingly, most of these sites were close to junctions (Appendix Fig S9B, colocalization, white). Comparison of Tie2‐pY992 in the inner and outer curvature of the aortic arch revealed stronger Tie2‐pY992 staining in the inner curvature after AKB‐9785 than after vehicle, but equally strong staining in the outer curvature with or without AKB‐9785 (Fig 8B and C), consistent with effects of VE‐PTP gene deletion in VE‐PTP^iECKO^ mice. The increase in Tie2 phosphorylation at 1 and 24 h after AKB‐9785 was confirmed in immunoblots of lung lysates (Appendix Fig S10A).

To determine whether AKB‐9785 could mimic the reduction in atheroma formation found after genetic deletion of VE‐PTP in endothelial cells, ApoE^−/−^ mice were treated with AKB‐9785 daily for 11 weeks while on a high‐fat diet for the final 10 weeks. Atheromas were as extensive in vehicle‐treated ApoE^−/−^ mice as in ApoE^−/−^/VE‐PTP^fl/fl^ controls at 10 weeks but after treatment with AKB‐9785 were 55% less in the aortic arch, 53% less in the brachiocephalic artery and 58% less around ostia of intercostal arteries (Fig 8D and E). Body weight and plasma cholesterol and triglyceride were similar regardless of treatment (Appendix Fig S10B and C). Together, the findings indicate that VE‐PTP inhibition by AKB‐9785 can increase Tie2 activation and reduce atherogenesis without changing plasma lipids while on a high‐fat diet.

## Discussion

This study sought to determine whether shear stress‐mediated sequestration of VE‐PTP in aortic endothelial cells promotes Tie2 activation, tightening the endothelial barrier, and reduced atherogenesis. We found that laminar flow with high average shear stress promoted the polarized redistribution of VE‐PTP in the plasma membrane to the downstream tip of endothelial cells *in vitro* and in regions of the aorta known to be resistant to atherosclerosis. VE‐PTP polarization and endocytosis reduced VE‐PTP on the cell surface, amplified Tie2 activation, and decreased plasma leakage. In contrast, regions of aortic endothelium exposed to disturbed flow, which are prone to atherogenesis, lacked VE‐PTP polarization, had less Tie2 activation, and greater leakage. Importantly, these proatherogenic properties were reduced by VE‐PTP gene deletion or pharmacological inhibition by AKB‐9785. Both approaches suppressed VE‐PTP phosphatase activity, promoted Tie2 activation, reduced plasma leakage, and decreased atheroma formation in atheroprone regions of the aorta. The findings revealed a novel mechanism whereby shear stress‐mediated sequestration of VE‐PTP at the downstream tip of endothelial cells has atheroprotective effects. VE‐PTP sequestration leads to Tie2 activation and tightening of the endothelial barrier. AKB‐9785 inhibition of VE‐PTP can reduce atheroma formation by mimicking this sequestration.

### Shear stress, VE‐PTP, Tie2 activity, and endothelial barrier function

We initially planned to determine the relative distributions of VE‐PTP, Tie2 activation, and leakage in the microvasculature, where Tie2 signaling and endothelial permeability have been extensively studied (Thurston *et al*, 2000; Kim *et al*, 2016), but individual endothelial cells proved difficult to analyze. These limitations led us to take advantage of the well‐documented regional differences in shear stress‐related permeability of the mouse aorta to elucidate links between VE‐PTP, Tie2‐pY992, and endothelial barrier function.

Tie2 activation is long known to increase endothelial junctional stability and reduce plasma leakage (Thurston *et al*, 2000; Frye *et al*, 2015; Claesson‐Welsh *et al*, 2021). The finding of greater Tie2‐pY992 and less plasma leakage in atheroprotective regions and weaker Tie2‐pY992 and more leakage in atheroprone regions fits with this evidence. Tie2 activation also leads to junctional recruitment of the GTPase exchange factor FGD5 (Braun *et al*, 2019), known to contribute to junctional stability (Braun *et al*, 2019; Pannekoek *et al*, 2020). The finding of uniform FGD5 at endothelial cell junctions in atheroprotected regions and FGD5 disruption in atheroprone regions of the aorta is consistent with the other evidence. Regulation of nuclear access of the transcription factor FoxO1 is another possible mechanism. Indeed, FoxO1 has been reported to modulate junction integrity (Taddei *et al*, 2008), and we found that flow‐induced effects on nuclear access of FoxO1 were dependent on Tie2.

VE‐PTP suppression of Tie2 phosphorylation can amplify plasma leakage (Winderlich *et al*, 2009; Shen *et al*, 2014; Frye *et al*, 2015). It follows, therefore, that Tie2 is activated and leakage is suppressed when VE‐PTP is redistributed by downstream sequestration by high average shear force.

Indeed, a causal link for this could be established, since silencing of VE‐PTP or Tie2 eliminated flow‐induced tightening of junctions in cultured endothelial cells. This mechanism is further supported by evidence that effects of shear force can be mimicked by genetic inactivation or pharmacologic inhibition of VE‐PTP. Additional support comes from reports of increased Tie2 tyrosine phosphorylation in cultured endothelial cells exposed to laminar flow (Lee & Koh, 2003; Tai *et al*, 2005). Laminar flow also promotes transcriptional upregulation of Tie2 (Savant *et al*, 2015), unlike disturbed flow that induces expression of the Tie2 agonist/antagonist, angiopoietin‐2 (Angpt2) (Kumar *et al*, 2014; Maurya *et al*, 2021). Still unknown is the time course of shear stress‐driven Tie2 phosphorylation relative to VE‐PTP polarization and internalization. In addition, a better understanding of aortic flow dynamics is needed to interpret the regional heterogeneity of Tie2 activation and VE‐PTP distribution in the endothelium.

### Shear stress‐driven VE‐PTP downstream polarization in endothelial cells

The finding of shear stress‐induced downstream polarization and endocytosis of VE‐PTP in aortic endothelial cells *in vivo* was confirmed by observing similar changes in HUVEC exposed to laminar flow. VE‐PTP in HUVEC grown under static conditions was redistributed to the downstream pole within 5 min of the onset of laminar flow. Internalization of VE‐PTP into endosomes was evident at 10 min. At 24 h, internalized VE‐PTP was less restricted to the downstream pole of HUVEC than in aortic endothelial cells, raising the possibility of high transmural pressure, pulsatile shear stress, and other flow dynamics in the aorta having effects on VE‐PTP redistribution not fully mimicked by continuous laminar flow *in vitro* over time. Interestingly, endocytosis of VE‐PTP was not blocked by interference with the functions of clathrin or dynamin, arguing for involvement of a non‐classical endocytosis mechanism (Maldonado‐Báez *et al*, 2013; Mayor *et al*, 2014).

The *in vitro* findings are consistent with results from bEnd.3 mouse endothelioma cells, HEK293A human embryonic kidney cells, and HUVEC in culture, where VE‐PTP polarization was identified and interpreted as an essential feature of flow induced cell alignment and elongation (Mantilidewi *et al*, 2014). A caveat is, however, that much of the VE‐PTP in sub‐confluent bEnd.3 cells is in intracellular recycling compartments, is barely detectable at cell contacts, and changes during growth to confluence (Nottebaum *et al*, 2008).

### Contribution of shear stress and VE‐PTP to endothelial barrier function

Tie2 activation by VE‐PTP redistribution and internalization in endothelial cells was a feature of aortic regions exposed to high average shear stress but not of atheroprone regions. However, the effects of disturbed flow in atheroprone regions were reduced and shifted toward the atheroprotective phenotype by genetic ablation or pharmacological inhibition of VE‐PTP. Although VE‐PTP gene inactivation during embryonic development causes severe defects of vascular development (Baumer *et al*, 2006; Dominguez *et al*, 2007; Winderlich *et al*, 2009), interference with VE‐PTP in adult mice is not accompanied by such side effects (Goel *et al*, 2013; Carota *et al*, 2019). Indeed, VE‐PTP^iECKO^ mice used in the present study had no noticeable abnormalities.

Flow effects on Tie2 activity are mediated by endothelial mechanotransducers. The mechanosensing properties of PECAM‐1 and formation of PECAM‐1/SHP2 complexes contribute through complexes of PECAM‐1, VE‐cadherin, and VEGFR2 (Tai *et al*, 2005; Tzima *et al*, 2005). Mechanotransducers that mediate disturbed flow effects on endothelial barrier function and inflammation are considered potential therapeutic targets for reducing atheroma formation (Tzima *et al*, 2005; Li *et al*, 2014; Conway *et al*, 2017; Albarran‐Juarez *et al*, 2018; Mehta *et al*, 2020). However, mechanotransducers not only sense shear force in atheroprone regions, but also mediate protective effects. Interfering with PECAM‐1 or PlxnD1 exacerbates atherogenesis in the aorta by inhibiting protective effects of shear stress (Goel *et al*, 2008; Mehta *et al*, 2020). Interfering with the Piezo1 mechanosensitive ion channel causes hypertension by blocking shear stress driven support of vasodilatation (Wang *et al*, 2021). Therapeutic strategies that interfere with mechanotransducers can thereby have mixed outcomes.

### Disrupted endothelial barrier function in atherogenesis

Impaired endothelial barrier function is a long‐recognized feature of atherogenesis (Doring *et al*, 2017; Sluiter *et al*, 2021). This feature is evident from the strong influence of regional differences in shear stress and plasma leakage on the distribution of atheromas in the aorta (Davies, 1995; Gimbrone & Garcia‐Cardena, 2013). Atheroprone regions that have disturbed blood flow also have greater endothelial leakiness. Plasma leakage is accompanied by extravasation of LDL and other proinflammatory molecules into the vessel wall (Davies, 1995; Gimbrone & Garcia‐Cardena, 2013). Blood monocyte/macrophages and other immune cells also enter the vessel wall when endothelial barrier disruption is accompanied by a chemotactic stimulus (Schulte *et al*, 2011; Sluiter *et al*, 2021). Although plasma leakage and leukocyte emigration usually occur at the same sites of barrier disruption, the two processes are regulated separately (Claesson‐Welsh *et al*, 2021).

Fibrinogen accumulates at sites of increased endothelial permeability predisposed to atherogenesis (Xiao *et al*, 1998; Reinhart, 2003). Based on this well documented feature, we determined the location and amount of plasma leakage in the aortic arch and thoracic aorta by using an approach developed for identifying sites of extravasation of anti‐fibrinogen antibody (Nakahara *et al*, 2006). The validity of the approach was confirmed by comparing the antibody to extravasation of rabbit IgG or 20‐nm fluorescent microspheres in the same regions. All were found to leak in the same locations, but the signal from rabbit IgG and microspheres was weaker in the normal mouse aorta, which fits previous observations in tumors (Nakahara *et al*, 2006). Anti‐fibrinogen antibody proved more informative because of retention by binding to extravasated fibrin at sites of historic fibrinogen leakage. This binding restricted clearance of antibody and increased sensitivity for localizing and measuring leakage.

### VE‐PTP inhibition compared with anti‐leakage effect of angiopoietins in atherosclerosis

Angiopoietin‐1 (Angpt1) reduces plasma leakage by activating Tie2 signaling (Thurston *et al*, 2000; Claesson‐Welsh *et al*, 2021), but compared with VE‐PTP, targeting the Angpt1‐Tie2 system has more complex effects in the context of atherosclerosis (Theelen *et al*, 2015; Fujisawa *et al*, 2017; Ou *et al*, 2020). Tie2 is expressed by cells of the hematopoietic lineage as well as by endothelial cells (Iwama *et al*, 1993; De Palma *et al*, 2005), unlike VE‐PTP that is restricted to endothelial cells. Activation of Tie2 by Angpt1 but not by VE‐PTP inhibition can, therefore, increase the number of myeloid cells in the circulation and promote influx of monocyte/macrophages into atheromas (Fujisawa *et al*, 2017).

Administration of Angpt2‐blocking antibodies can reduce atheromas in LDLR^−/−^/ApoB^100/100^ mice (Theelen *et al*, 2015). However, peripheral edema and other adverse effects can occur in patients treated with anti‐Angpt2 antibodies for cancer (Papadopoulos *et al*, 2016; Martin‐Liberal *et al*, 2020). More work is needed to determine the feasibility of long‐term administration of Angpt2 inhibitors for atherosclerosis.

AKB‐9785 and AKB‐9778 are potent inhibitors of VE‐PTP that have beneficial actions in models of retinopathy, glaucoma, diabetic nephropathy, and metastasis (Goel *et al*, 2013; Shen *et al*, 2014; Carota *et al*, 2019; Li *et al*, 2020) and have a favorable safety profile in animals and adult humans (Campochiaro *et al*, 2015, 2016; Li *et al*, 2020; Siragusa *et al*, 2021). Peripheral edema was not found in patients treated by subcutaneous injection of the VE‐PTP inhibitor AKB‐9778 for diabetic macular edema (Campochiaro *et al*, 2015, 2016). This is likely to be a consequence of the selectivity of AKB‐9778 for VE‐PTP and the restricted distribution of VE‐PTP in endothelial cells (Vestweber, 2021). Further evaluation of Tie2 activation in endothelial cells by VE‐PTP inhibitors in atherosclerosis is warranted to learn more about the potential therapeutic benefit.

### VE‐PTP contribution to atherogenesis in ApoE^−/−^ mice

The diverse consequences of hyperlipidemia, including altered endothelial barrier function, inflammatory changes, and atherogenesis (Wu *et al*, 1995; Baumer *et al*, 2017), prompted studies of ApoE^−/−^/VE‐PTP^iECKO^ mice that were found to have less plasma leakage and atherogenesis. Although hyperlipidemia and disturbed blood flow can also promote NFκB activation and expression of inflammatory cytokines and leukocyte adhesion molecules, endothelial cell VE‐PTP deletion in ApoE^−/−^/VE‐PTP^iECKO^ mice was not accompanied by changes in ICAM1 or VCAM1 in aortic endothelial cells. Nor were changes found in the plasma lipid profile of these mice. These findings point to reduced plasma leakage as a primary consequence of VE‐PTP deletion in ApoE^−/−^/VE‐PTP^iECKO^ mice. However, it cannot be excluded that tightening of endothelial junctions also reduced leukocyte entry into atheromas, as VE‐PTP inhibition can reduce leukocyte extravasation in inflammation models (Frye *et al*, 2015). Although we assume that suppression of atherogenesis by VE‐PTP deletion is not specific to ApoE^−/−^ mice, other models should be examined to develop a more complete understanding of the potential of VE‐PTP as a therapeutic target in atherosclerosis.

## Conclusions

**Figure 9:**
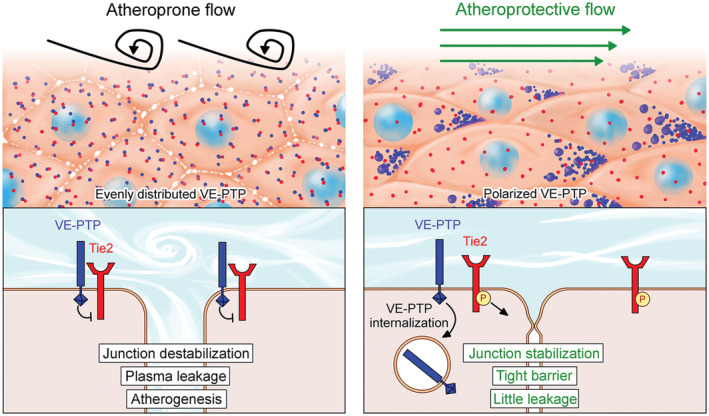
Proposed mechanism of VE‐PTP regulation of endothelial barrier function and atherogenesis in response to shear force In atheroprone regions of the aorta with disturbed flow and low average shear force (left), VE‐PTP‐Tie2 complexes in the plasma membrane promote Tie2 dephosphorylation, destabilization of intercellular junctions, increased permeability (small gaps between cells), and greater plasma leakage. Endothelial leakiness favors the development of atherosclerotic plaques under atherogenic conditions of lipid metabolism. By comparison, in atheroprotected regions, laminar flow with high average shear force (right) stimulates VE‐PTP (blue) redistribution to the downstream pole of endothelial cells, followed by internalization into endosomes. This change sequesters VE‐PTP from Tie2 (red), promotes Tie2 phosphorylation (yellow), stabilizes intercellular junctions, tightens the endothelial barrier, and reduces leakage. Inhibition of VE‐PTP drives the process from left to right, tightens the endothelial barrier, reduces leakage, and suppresses atherogenesis.

Together, the findings provide evidence that high average shear stress in atheroprotected regions of the aorta promotes downstream polarization and endocytosis of VE‐PTP from the plasma membrane of endothelial cells (Fig 9). This sequestration of VE‐PTP from Tie2 is accompanied by increased Tie2 signaling, tightening of the endothelial barrier, and less atheroma formation. By comparison, in endothelial cells exposed to disturbed blood flow, VE‐PTP remains in the plasma membrane where its phosphatase activity reduces Tie2 signaling, destabilizes the endothelial barrier, and predisposes to atherogenesis. This proatherogenic effect is reduced by deletion of VE‐PTP in endothelial cells or by blocking the phosphatase activity with AKB‐9785. Both approaches increase Tie2 activity, reduce plasma leakage, and suppress atherogenesis without changing ICAM1 or VCAM1 in endothelial cells. The findings provide proof of principle for future translational studies of VE‐PTP inhibition in atherosclerosis.

## Materials and Methods

### Mice

Wild‐type C57BL/6 mice of both genders, bred at UCSF or the Max Planck Institute for Molecular Biomedicine, were used at ages 8–16 weeks for studies of normal aorta. VE‐PTP inducible endothelial cell‐specific knockout (VE‐PTP^iECKO^) mice were generated by breeding Ptprb lox/lox mice to Pdgfb‐iCre mice (Claxton *et al*, 2008) as previously described (Frye *et al*, 2015). Tie2 inducible cell‐specific knockout (Tie2^iECKO^) mice were generated by breeding Tek^lox/lox^ mice to Pdgfb‐iCre mice as previously described (Braun *et al*, 2020). Apolipoprotein E (ApoE)^−/−^ mice (Piedrahita *et al*, 1992) were purchased from The Jackson Laboratory (#002052).

ApoE^−/−^/VE‐PTP^fl/fl^ mice were crossed with ApoE^−/−^/VE‐PTP^iECKO^ mice to produce ApoE^−/−^/VE‐PTP^fl/fl^ mice and ApoE^−/−^/VE‐PTP^iECKO^ mice that are littermates for use in the experiments. Mice were anesthetized with ketamine (87 mg/kg) and xylazine (10 mg/kg) injected intraperitoneally before invasive procedures. Experiments included approximately equal numbers of males and females.

VE‐PTP^iECKO^ mice and ApoE^−/−^/VE‐PTP^iECKO^ mice at age 6–8 weeks received 5 consecutive daily intraperitoneal injections of 100 μl tamoxifen (T5648, Sigma‐Aldrich) at a concentration of 30 mg/ml in 2% ethanol and 98% peanut oil. VE‐PTP^fl/fl^ mice and ApoE^−/−^/VE‐PTP^fl/fl^ mice also received tamoxifen. ApoE^−/−^, ApoE^−/−^/VE‐PTP^fl/fl^, and ApoE^−/−^/VE‐PTP^iECKO^ mice were fed a high‐fat (Western type) diet (21% fat, 0.25% cholesterol, and 19.5% casein, Altromin, Germany) for 7 or 10 weeks.

All mice were raised in a barrier facility at UCSF or the Max Planck Institute for Molecular Biomedicine under special pathogen‐free conditions and given food and water ad libitum.

### Study approval

All animal studies were approved by the Institutional Animal Care and Use Committee of the University of California, San Francisco, USA (Approval AN183863‐02) or the Landesamt für Natur, Umwelt und Verbraucherschutz Nordrhein‐Westfalen, Germany (Approval 81‐02.04.2020.A023).

### Treatment with AKB‐9785

The VE‐PTP inhibitor AKB‐9785 (Braun *et al*, 2019; Siragusa *et al*, 2021) is a closely related chemical congener of AKB‐9778 (Shen *et al*, 2014) with similar sub‐nanomolar potency for VE‐PTP and similar high degree of selectivity against other phosphatases. AKB‐9785 was administered by subcutaneous injection of a dose of 30 mg/kg (100 μl of 10 mM AKB‐9785 to 20 g mice), once at 1 h before or twice at 1 and 6 h before study of wild‐type mice, or daily for 11 weeks to ApoE^−/−^ mice on a high‐fat diet for 10 weeks. Corresponding controls received injections of vehicle.

### Staining of aorta by immunohistochemistry or Oil Red O

Anesthetized mice were perfused with 1% paraformaldehyde (PFA), the aorta was removed, fixed in 1% PFA for a further hour and washed with PBS, incised longitudinally to create flattenable whole mounts, and permeabilized in a mixture of 0.3% Triton X‐100 for 1 h. Specimens were incubated in primary antibodies diluted in 10% donkey serum in PBS with 0.3% TritonX‐100 at room temperature overnight, washed the next day, and stained overnight in secondary antibodies in PBS. After washing, aortas were mounted on glass slides in Vectashield (Vector Laboratories) containing 50 mg/ml DAPI (Sigma) or Dako Fluorescence Mounting Medium (Agilent Technologies).

For analyzing atheromas in mice with ApoE deletion, after perfusion fixation, the aorta was removed and atheromas were stained with Oil Red O (O1391, Sigma‐Aldrich).

### Histological analysis of aortic root

Anesthetized mice were perfused with 1% paraformaldehyde (PFA), the heart was removed, the upper half of the heart was embedded in optimum cutting temperature (OCT) compound and frozen at −80°C. Blocks were sectioned at 8 μm intervals using a cryostat (Thermo Scientific). Sections were fixed for 10 min with 4% PFA and stained with Oil Red O (O1391, Sigma‐Aldrich).

### Antibodies

Primary antibodies used for immunohistochemical staining or other experiments included: rat monoclonal anti‐mouse **VE‐PTP** (clone 109.1 (Baumer *et al*, 2006), 10 μg/ml); rabbit polyclonal anti‐human **VE‐PTP** (VE‐PTPh1‐8 (Li *et al*, 2020), 10 μg/ml for immunohistochemical staining or 0.5 μg/ml for Cell‐ELISA); mouse monoclonal anti‐human **Tie2** (clone Ab33 #05‐584, Sigma, 5 μg/ml for immunohistochemical staining, 1 μg/ml for immunoblotting or 5 μg for immunoprecipitation); goat polyclonal anti‐mouse **Tie2** (AF762, R&D Systems, 1 μg/ml); rabbit polyclonal anti‐mouse/human **phospho‐Tie2** (pY992, AF2720, R&D Systems, 5 μg/ml); mouse monoclonal anti‐mouse/human **phosphotyrosine** (4G10, #05‐321, Sigma, 0.5 μg/ml); rabbit polyclonal anti‐mouse **VE‐cadherin** (VE42 (Broermann *et al*, 2011), 5 μg/ml for immunohistochemical staining or 1 μg/ml for immunoblotting); rabbit monoclonal anti‐mouse **VE‐cadherin** (Vii37, #MABT886, Sigma, 1:1,000); goat polyclonal anti‐mouse **VE‐cadherin** (AF1002, R&D Systems, 1 μg/ml); mouse monoclonal anti‐human **VE‐cadherin** (clone F‐8, sc‐9989, Santa Cruz Biotechnology, 1:100); rabbit monoclonal anti‐**EEA1** peptide (clone C45B10, #3288, Cell Signaling, 1:500); mouse monoclonal anti‐human **EEA1** (clone 14/EEA1, #610456, BD Bioscience. 1:100); rabbit monoclonal anti‐**Rab5** peptide (clone C8B1, #3547, Cell Signaling, 1:500); rat monoclonal anti‐mouse **ICAM1** (clone YN1/1.7.4, # 12‐0541‐81, eBioscience, 5 μg/ml); rat monoclonal anti‐mouse **VCAM1** (clone 429, #550547, BioLegend, 5 μg/ml); chicken polyclonal anti‐human **lamin A/C** (NBP‐2‐25152, Novus, 1:500); rabbit monoclonal anti‐human **FoxO1** (C29H4, #2880, Cell Signaling, 1:200); rabbit monoclonal anti‐human **RhoA** (EPR18134, Abcam, 1:2,000); mouse monoclonal anti‐**CD31/PECAM1** (clone H‐11, sc‐376864, Santa Cruz Biotechnology, 1:100). Secondary antibodies conjugated to Cy3, Alexa Fluor 488, Alexa Fluor 568, Alexa Fluor 594, Alexa Fluor 647 (1:500) or Horseradish Peroxidase (1:5,000) were from Jackson ImmunoResearch or Invitrogen.

### Measurement of vascular leakage in aorta

Unanesthetized mice received an i.v. injection of 5 mg/kg anti‐fibrinogen antibody (A0080, rabbit anti‐mouse, DAKO) and 30 min later were anesthetized and perfused through the heart with 1% PFA. In validation experiments, mice received an i.v. injection of 5 mg/kg normal rabbit IgG (Dako DX090302‐8 or Jackson ImmunoResearch 011‐000‐002) in a volume of 100 μl PBS or 1 ml/kg of undiluted 20‐nm red fluorescent polystyrene microspheres (Invitrogen F8786). After perfusion fixation, the aorta was removed and stained overnight with Cy3‐labeled secondary antibody to detect extravasated antibody or rabbit IgG.

Extravasation of tracer anti‐fibrinogen antibody or IgG was assessed by measuring the intensity of immunofluorescence in flattened whole mounts of the aortic arch after immunohistochemical staining. Regions measuring 240 by 320 μm with brightest fluorescence in the inner curvature and outer curvature were photographed with an Olympus DP73 digital camera (exposure 200 ms, images 600 × 800 pixels) on a Zeiss Axiophot fluorescence microscope (objective lens ×10, NA 0.5). The amount of extravasated tracer was expressed as the distribution of fluorescence intensities over the range of 0–255 and mean fluorescence intensity of the red channel of RGB images analyzed with ImageJ Analyze/Measure and Analyze/Histogram tools.

### Measurement of VE‐PTP in aortic endothelial cells

Immunohistochemically stained whole mounts of aortas were examined with Zeiss LSM 510 or LSM 880 confocal microscopes (×40 NA 1.0, ×63 NA 1.4, or ×100 NA 1.4 oil immersion objectives). Confocal microscopic images (1,024 × 1,024 pixels) were imported into ImageJ/Fiji, and the size, abundance, and polarization of red dot‐like VE‐PTP immunoreactivity (particles) were measured with the Analyze/Analyze particles tool. Data were transferred to Excel (Microsoft) or Prism (GraphPad Software) for analysis of particle size, occupied area, and fluorescence intensity cumulative frequency curves. VE‐PTP particle size calculations excluded objects <50 pixels, which could not be distinguished from background noise.

VE‐PTP polarization in individual endothelial cells was analyzed in confocal microscopic images (×100 NA 1.4). After the cell border was outlined and cell area was measured, the amount of red channel fluorescence (VE‐PTP staining) above an intensity threshold of 150 (range 0–255) was measured in the upstream and downstream halves. The distribution of VE‐PTP was expressed as the proportion and fractional area occupied in each half (Fig 1D, Appendix Fig S1A and B).

### Measurement of Tie2‐pY992 at aortic endothelial cell junctions

Tie2 phosphorylation at endothelial cell junctions was measured in cells stained for VE‐cadherin to define the cell border and act as a mask for Tie2‐pY992 immunoreactivity. Confocal microscopic images (1,024 × 1,024 pixels, Zeiss LSM 880 inverted confocal microscope with ×63 NA 1.4 oil immersion objective) were imported into ImageJ/Fiji. The VE‐cadherin signal above a fluorescence intensity threshold of 10 (0–255) was converted into a binary image with ImageJ/Fiji (Process/Binary/Convert to Mask tool). The Process/Binary/Close tool was used for two iterations to make a continuous border, and particles smaller than 10 pixels were removed with the Analyze/Analyze Particles tool. Tie2‐pY992 at cell junctions was extracted by applying the mask to the images of Tie2‐pY992 using the ‘Image Calculator/Operation AND’ tool. Intensity values in the mask were exported to Excel and normalized to the size of the mask. Values were expressed as Tie2‐pY992 at cell junctions. Alternatively, when endothelial cells were not stained for VE‐cadherin, the Tie2‐pY992 signal was measured using ImageJ/Fiji ‘Straight Line’, ‘Plot Profile’ and ‘Measure’ tools after 25–30 straight lines 21‐pixels wide were drawn on each endothelial cell perpendicular to the cell junctions. At least 100 lines were analyzed per mouse. The fluorescence intensity of Tie2‐pY992 was measured where lines intersected the cell border using the ‘Plot Profile’ and ‘Measure’ tools. Tie2‐pY992 values at cell junctions in the inner curvature were expressed relative to values in the outer curvature.

### Measurement of Oil Red O‐stained atheromas

Atherogenesis was assessed by measuring Oil Red O staining in digital images of opened aortic whole mounts (Figs 7A and 8D). RGB images were obtained with a Zeiss Axiocam 305 color camera on a Leica M205C dissecting microscope. The aortic arch and base of branches, excluding the aortic root next to the heart, was outlined with ImageJ/Fiji for analysis (dimensions approximately 10.9 × 9.1 mm, 1,232 × 1,028 pixels). The red channel was analyzed after removing reflections and other non‐red features by subtracting the green and blue channels using ImageJ/Fiji tools ‘Image/Color/Spilt Channels > Process/Image Calculator/Subtract’. The number of red pixels with an intensity above 5 (range 0–255) was expressed as a percentage of total pixels (area density %). In the same images, staining was also measured in 200‐μm diameter circular regions of proximal thoracic aorta centered over intercostal artery ostia or away from ostia (6 of each per aorta).

### Measurement of ICAM1 and VCAM1 in aortic arch

The amount of ICAM1 and VCAM1 immunoreactivity in endothelial cells of the outer and inner curvatures of the aortic arch was measured in confocal microscopic images as the mean fluorescence intensity.

### Exposure of HUVEC to shear stress *in vitro*

HUVEC were isolated from umbilical cords (Ethics Committee of Münster University Clinic Approval 2009‐537‐f‐S) by treatment with 1 unit/ml Dispase II (Roche) for 10 min at 37°C in M199 medium containing 1% penicillin/streptomycin, 20% fetal calf serum (FBS), 100 μg/ml heparin, and 3.1 μg/ml fungizone. HUVEC were cultured in EGM‐2 medium (Lonza) in a humidified incubator (37°C, 95% air/5% CO_2_) and used for experiments between passages 3 and 6. The culture medium was replaced by EBM‐2 medium containing 2% FBS and 1% penicillin/streptomycin for 4 h before effects of flow were studied. Shear stress of 15 dyn/cm^2^ was applied for 5 min to 24 h (10902 Pump System, ibidi GmbH, Germany) at 37°C in the same humidified incubator. For immunofluorescence staining and cell‐based ELISA, HUVEC were plated on fibronectin‐coated slides (80606 VI 0.4 μ‐slides, ibidi GmbH) at 6 × 10^4^ cells/lane, treated with siRNA (66.7 nM) in some experiments, and incubated for 48 h. For biochemical assays, HUVEC were plated on fibronectin‐coated slides (80176 I Luer 0.4 μ‐slides, ibidi GmbH) at 2.5 × 10^5^/lane, treated with siRNA (160 nM) in some experiments, and incubated for 48 h.

HUVEC were fixed for immunohistochemistry with 4% PFA in PBS for 15 min. In some cases, fixed cells were permeabilized with 0.3% Triton X‐100 in PBS for 5 min, blocked in 1% BSA for 1 h at room temperature, and incubated with primary antibody at 4°C overnight, washed, and then incubated with secondary antibodies and Hoechst 33342 (Invitrogen H3570) for 1 h at room temperature.

### Small interfering RNA (siRNA)‐mediated gene silencing in HUVEC

VE‐PTP expression in HUVEC was silenced by a combination of two siRNA against VE‐PTP (Hs_PTPRB_5, 5′‐UAACUUGAUAAAGUCGACCGG‐3′ and Hs_PTPRB_10 5′‐UAUCGUUCCACAUUCCCAGAA‐3′, Qiagen); Tie2 was silenced with siRNA 5′‐TCGGTGCTACTTAACAACTTA‐3′ (SI00604919, Qiagen). VE‐cadherin was silenced siRNA 5′‐GGGUUUUUGCAUAAUAAGCTT‐3′ (10696, AMBION). RhoA was silenced with siRNA (sc‐29471, Santa Cruz Biotechnology). Allstar negative siRNA (Qiagen) was used as a negative control. siRNA was transfected using Lipofectamine RNAiMAX (Invitrogen) according to the manufacturer's protocol.

### VE‐PTP subcellular localization in the presence of inhibitors

HUVECs were pretreated with SU‐1498 (SML1193, Sigma Aldrich) at 20 μM for 4 h or Hydroxy‐Dynasore (SML0340, Sigma Aldrich) at 100 μM or Pitstop‐2 (SML1169, Sigma Aldrich) at 30 μM for 1 h. The resulting cells were exposed to 15 dyn/cm^2^ of shear stress for 5, 10 and 30 min. The cells were fixed and analyzed by immunofluorescence staining for VE‐PTP and VE‐cadherin.

### EEA1 colocalized with VE‐PTP in HUVEC

EEA1 immunofluorescence in HUVEC above an intensity threshold of 10 (range 0–255) was converted into a binary image with ImageJ/Fiji (Process/Binary/Convert to Mask tool). EEA1 colocalized with VE‐PTP immunoreactivity was extracted by applying the EEA1 mask to the VE‐PTP image with the ‘Image Calculator/Operation AND’ tool. The amount of EEA1 colocalized with VE‐PTP was expressed as percent of total EEA1 immunofluorescence.

### VE‐PTP cell‐based ELISA in HUVECs

HUVEC exposed to shear stress for 5, 10, or 30 min were fixed in 4% PFA in PBS for 15 min and blocked in 1% BSA for 1 h at room temperature followed by incubation with 0.5 μg/ml of primary antibodies or isotype control IgG at 4°C overnight. Primary antibodies were detected with peroxidase‐conjugated secondary antibody and o‐phenylenediamine dihydrochloride (OPD) substrate (Thermo Fisher Scientific). Optical density (OD) at 490 nm was measured with a plate reader (Synergy 2, LabTek). Data in each experiment were normalized to OD490 values in empty lanes.

### Measurement of Tie2‐pY992 at cellular junctions in HUVECs

Tie2 phosphorylation at cellular junctions was measured with HUVEC stained for VE‐cadherin to define the cell border and act as a mask for Tie2‐pY992 immunoreactivity. Confocal microscopic images (1,024 × 1,024 pixels, Zeiss LSM 880 inverted confocal microscope with ×40 NA 1.0 oil immersion objective) were imported into ImageJ/Fiji. The VE‐cadherin signal above a fluorescence intensity threshold of 10 (0–255) was converted into a binary image with ImageJ/Fiji (Process/Binary/Convert to Mask tool). The Process/Binary/Close tool was used for two iterations to make a continuous border, and particles smaller than 10 pixels were removed with the Analyze/Analyze Particles tool. Tie2‐pY992 at cell junctions was extracted by applying the mask to the images of Tie2‐pY992 using the ‘Image Calculator/Operation AND’ tool. Intensity values in the mask were exported to Excel and normalized to the size of the mask. Values were expressed as Tie2‐pY992 at cell junctions.

### Quantification of FoxO1 in HUVECs

The Hoechst 33342 signal was applied to the filter ‘Gaussian Blur at Sigma = 1.5’ and converted into binary image with AutoThreshold ‘Huang’. Nuclear FoxO1 signals were extracted by applying the mask to the image of FoxO1 using the ‘Image Calculator/Operation AND’ tool. Nuclear FoxO1 values were expressed relative to values in HUVECs treated with control siRNA and analyzed under static condition.

### Measurement of HUVEC permeability with or without flow

HUVEC transfected with control siRNA, VE‐PTP siRNA, Tie2 siRNA, or VE‐cadherin siRNA were plated on ibidi μ‐slides VI 0.4 coated with biotinylated gelatin as described (Dubrovskyi *et al*, 2013). Confluent HUVEC exposed to static conditions or shear stress for 30 min were incubated in medium containing streptavidin‐Alexa Fluor 647 conjugate (S2374, Invitrogen) at 2 μg/ml for 3 min. Subsequently, HUVEC were washed three times and stained for immunofluorescence as described above. The area of streptavidin‐Alexa 647 in images (1,024 × 1,024 pixels), obtained with a Zeiss LSM 780 inverted confocal microscope (×20 NA 0.80), was measured as number of pixels above a fluorescence intensity of 20 (range 0–255) with ImageJ/Fiji and expressed as percentage of total area (area density, %).

### Immunoprecipitation and immunoblotting

Target proteins were measured in HUVEC lysed in buffer containing 20 mM Tris–HCl, pH 7.4, 150 mM NaCl, 2 mM CaCl_2_, 1.5 mM MgCl_2_, 1 mM Na_3_VO_4_, 1% Triton X‐100, 0.04% NaN_3_ and 1× cOmplete EDTA‐free Proteinase Inhibitor Cocktail (Roche). The proteins were also measured in lungs homogenized with a Precellys Evolution homogenizer (Bertin Instruments) in RIPA buffer containing 1% NP‐40, 1% sodium deoxycholate, 0.1% SDS, 0.01 M NaHPO_4_, 150 mM NaCl, 2 mM EDTA, 1 mM DTT and 2× cOmplete Protease Inhibitors (Roche). Immunoblots were prepared from lysates incubated for 2 h or overnight at 4°C with Protein G Sepharose beads and mouse monoclonal Ab33 anti‐human Tie2 for HUVEC or rat monoclonal 3G1 anti‐mouse Tie2 for lung. Precipitated complexes were washed five times with lysis buffer and eluted from beads with Laemmli sample buffer. Proteins were separated by SDS–PAGE and transferred to nitrocellulose membranes (Schleicher & Schuell) by wet blotting.

Target proteins in HUVEC were detected by mouse monoclonal Ab33 anti‐human Tie2, rabbit polyclonal VE‐PTP‐c anti‐human/mouse VE‐PTP, and mouse monoclonal 4G10 anti‐phosphorylated tyrosine, and in lung lysates by rabbit polyclonal VE‐PTP‐c anti‐human/mouse VE‐PTP, rabbit polyclonal VE‐42 anti‐mouse VE‐cadherin, rat monoclonal 3G1 anti‐mouse Tie2, and mouse monoclonal 4G10 anti‐phosphorylated tyrosine. Blocking buffer contained 2% BSA and 200 μM Na_3_VO_4_ instead of 3% skimmed milk when phosphorylated proteins were detected. Phosphorylated Tie2 was standardized to the expression of total precipitated Tie2. Expression of VE‐PTP in lung lysates was standardized to VE‐cadherin.

### Statistical analysis

Mice of both genders matched for age were randomly assigned to groups. Group size was determined by power analysis of data from pilot studies to achieve statistical power of 0.8 and *P* value of 0.05. Gender was tested as a biological variable. Analysis was done in a non‐blinded fashion. Values are expressed as mean ± standard error of the mean (SEM) for each group, where the number of mice per group is shown in figure legends. Differences between two groups were assessed by two‐tailed Welch's *t*‐test or Student's *t*‐test and among more than two groups were assessed by ANOVA followed by Bonferroni correction test, Dunnett's test, or Tukey test for multiple comparisons (Prism). Distributions of fluorescence intensities were compared by the Kolmogorov–Smirnov two‐sample test. The statistical significance of differences is shown in figure legends. *P*‐values < 0.05 were considered statistically significant.

**The paper explained**

**Problem**

Laminar blood flow and high shear stress are well known to protect against transendothelial plasma leakage and thereby reduce susceptibility to atherosclerotic plaque formation in the aorta, where turbulent flow has the opposite effects, but the mechanism underlying the protective actions of laminar flow is unknown.

**Results**

We found that laminar flow and high shear stress triggered the sequestration of VE‐PTP, an endothelial‐specific receptor type tyrosine phosphatase, to the downstream pole of endothelial cells away from its substrate, the tyrosine kinase receptor Tie2. As a consequence, Tie2 and downstream targets became activated at cell junctions, which strengthened endothelial barrier function, suppressed plasma leakage, and reduced atheroma formation in ApoE^−/−^ mice on high‐fat diet. Reduction in atherosclerosis was revealed by creating a new strain of ApoE^−/−^ mice that lack VE‐PTP in endothelial cells. The findings in mice with genetic deletion of VE‐PTP were confirmed in conventional ApoE^−/−^ mice treated with the pharmacologic inhibitor AKB‐9785 that selectively blocks VE‐PTP and has a known safety profile in humans.

**Impact**

These novel findings of the contribution of VE‐PTP and Tie2 signaling to atherogenesis have translational relevance as proof of principle for future pharmacological trials and identify VE‐PTP as a potential new target for reducing atherosclerosis.

## Author contributions

**Keisuke Shirakura:** Conceptualization; data curation; formal analysis; validation; investigation; visualization; writing – original draft; writing – review and editing.§ **Peter Baluk:** Conceptualization; data curation; formal analysis; investigation; visualization; writing – original draft; writing – review and editing. **Astrid F Nottebaum:** Data curation; formal analysis; validation; investigation; writing – review and editing. **Ute Ipe:** Validation; investigation. **Kevin G Peters:** Resources; writing – review and editing. **Donald M McDonald:** Conceptualization; supervision; funding acquisition; writing – original draft; project administration; writing – review and editing. **Dietmar Vestweber:** Conceptualization; supervision; funding acquisition; writing – original draft; project administration; writing – review and editing. Open Access funding enabled and organized by Projekt DEAL.

## Disclosure and competing interests statement

The authors declare that they have noconflict of interest apart from Kevin Peters, who is a holder of stock or stock options in Aerpio Pharmaceuticals and is a patent holder of AKB‐9785.

## Supporting information



Appendix

Expanded View Figures PDF

PDF+

Source Data for Figure 1

Source Data for Figure 5

## Data Availability

This study includes no data deposited in external repositories.

## Footnotes

^§^
Correction added on 11 April 2023, after first online publicaiton: the author contributions of Peter Baluk have been updated.
