# Supplementary material for: Shear stress control of vascular leaks and atheromas through Tie2 activation by VE‐PTP sequestration
Source: EMBO Mol Med. 2023 Feb 6;15(4):e16128. doi: 10.15252/emmm.202216128 (PMC10086590; doi:10.15252/emmm.202216128)
Supplement: Supplementary file 1 — Appendix [file EMMM-15-e16128-s003.pdf]

Table of content

Appendix Figure S1. VE-PTP polarization and deletion in VE-PTP^{iECKO} mice or by siRNA.

Appendix Figure S2. Flow-induced downstream polarization of VE-PTP in HUVEC.

Appendix Figure S3. VEGFR2 and RhoA are not essential for shear stress-mediated redistribution of VE-PTP.

Appendix Figure S4. Validation of method for leakage measurement.

Appendix Figure S5. Specificity of Tie2-pY992 antibodies.

Appendix Figure S6. VE-PTP deletion in ApoE$^{-/-}$ mice without changes in body weight and plasma lipids.

Appendix Figure S7. Oil Red O staining of atheromas in the aortic root.

Appendix Figure S8. Contrasting staining of Tie2-pY992 and VE-cadherin at endothelial cells of normal region and over atheroma.

Appendix Figure S9. VE-PTP inhibitor AKB-9785 increases Tie2-pY992 at both cellular junctions and focal adhesions.

Appendix Figure S10. Increased Tie2 phosphorylation after AKB-9785 without changes in body weight or plasma lipids.

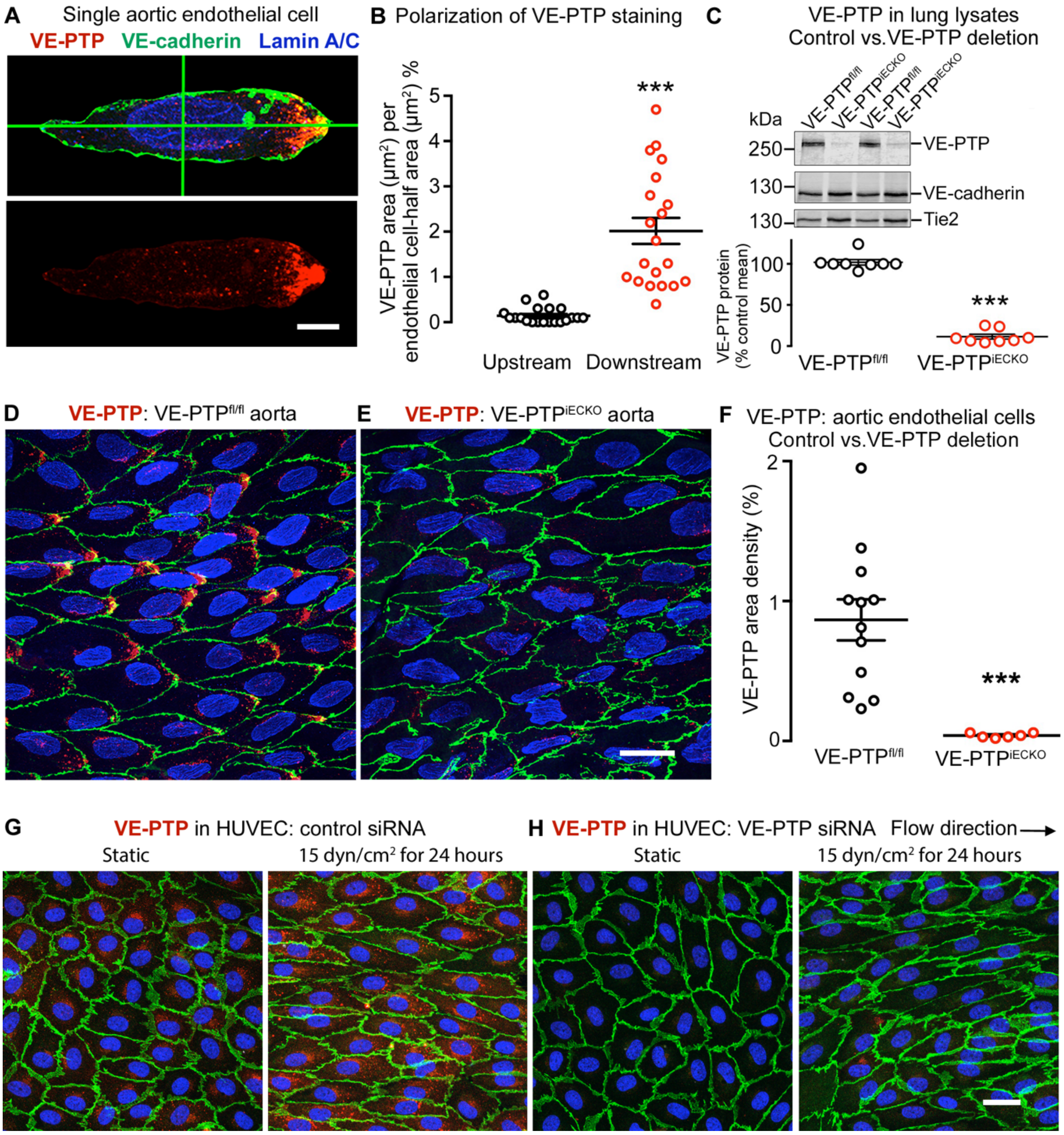

Appendix Figure S1. VE-PTP polarization and deletion in VE-PTP^{iECKO} mice or by siRNA. **(A-B)** Images and measurements documenting VE-PTP polarization (red) in individual endothelial cells in thoracic aorta of wild-type mouse. Values in B compare the amounts of VE-PTP staining in each cell half. Each dot represents half of one endothelial cell. Mean ± SEM, n = 15 cells. **(C)** Assessment of VE-PTP deletion (89%) by immunoblot of lung lysates. VE-PTP protein expressed as a ratio to VE-cadherin protein. Mean ± SEM, n = 8 mice/group. **(D-E)** Comparison of VE-PTP staining (red) of endothelial cells in thoracic aorta of VE-PTP$^{fl/fl}$ mouse and VE-PTP^{iECKO} mouse. **(F)** Assessment of VE-PTP deletion (95%) in E by immunohistochemical staining. Mean ± SEM, n = 6-12 mice/group. **(G-H)** Comparison of staining for VE-PTP (red), VE-cadherin (green), and nuclei (blue, Hoechst 33342) in HUVEC after control siRNA in G or VE-PTP siRNA in H under static conditions or laminar flow of 15 dyn/cm^2 for 24 h. ***P < 0.001, by Student's t-test in B and F and Mann-Whitney-test in C. Scale bars: 10 µm in A, 20 µm in D, E, G and H.

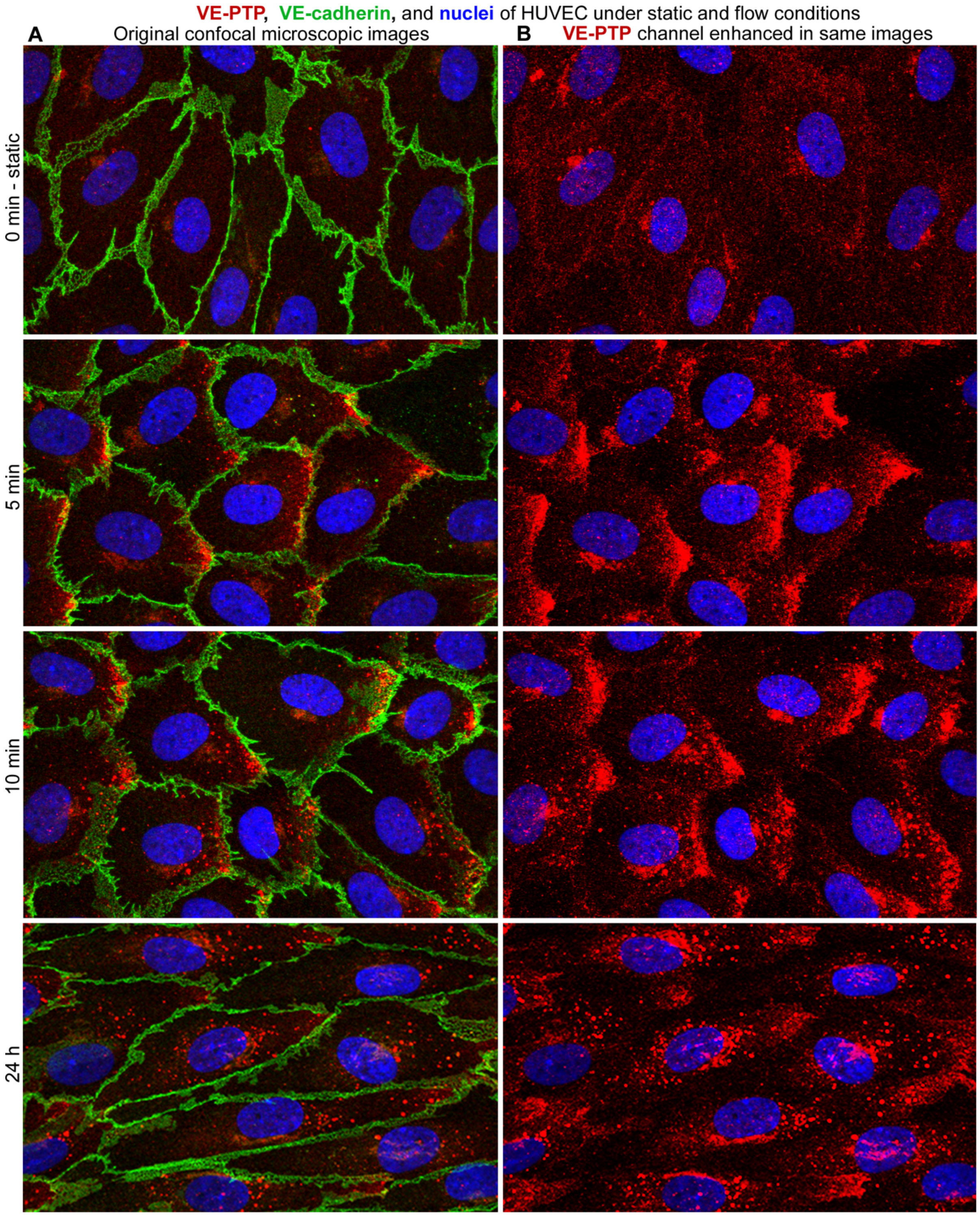

Appendix Figure S2. Flow-induced downstream polarization of VE-PTP in HUVEC. (**A-B**) Enlargements of images in Figure 1G showing VE-PTP staining (red) in HUVEC under static conditions and laminar flow (15 dyn/cm^2) for 5 min to 24 h to compare the original confocal microscopic images in A to the same images in B after the brightness and contrast of red channel (VE-PTP) were increased to highlight the flow-induced changes. VE-cadherin, green. Nuclei, blue (Hoechst 33342). All images in B were treated identically. Faint VE-PTP staining at the cell border and near the nucleus under static conditions contrasts with downstream polarization after flow and change from diffuse downstream staining at 5 min to

dot-like downstream staining at 10 min, and more widespread dot-like staining at 24 h. Scale bar: 10 μm.

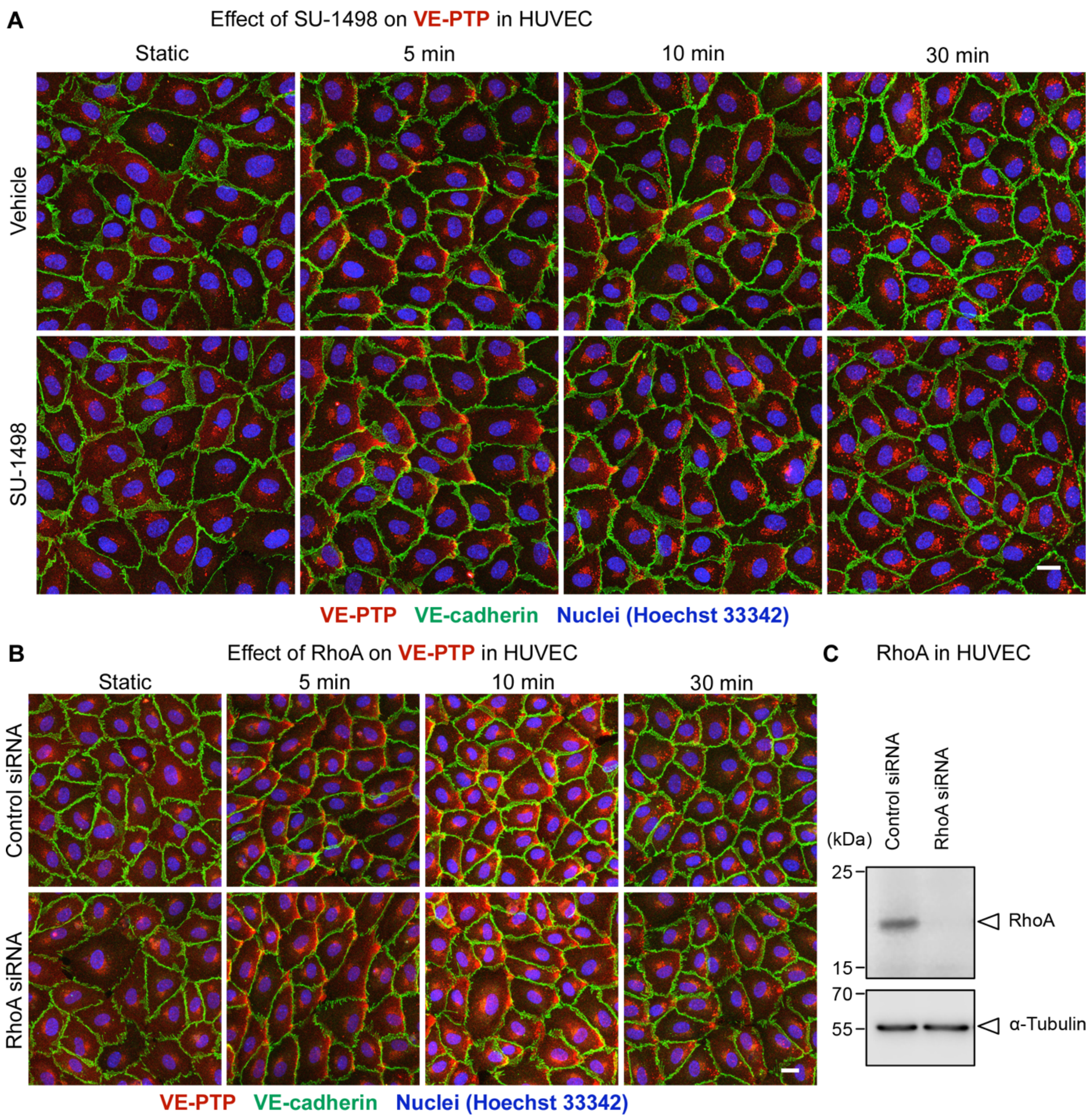

Appendix Figure S3. VEGFR2 and RhoA are not essential for shear stress-mediated redistribution of VE-PTP. (A) HUVECs, treated with DMSO or SU-1498 for four hours, were exposed to 15 dyn/cm2 of shear stress for indicated times. The resulting cells were fixed and stained for VE-PTP (red) and VE-cadherin (green) and Hoechst 33342 (blue). **(B)** HUVECs treated with control siRNA or RhoA siRNA were exposed to shear and stained similarly as under A. **(C)** Cell lysates of HUVEC treated with control siRNA or RhoA siRNA were immunoblotted for RhoA and tubulin, as indicated. Molecular weights are given on the left. Scale bar: 20 μm.

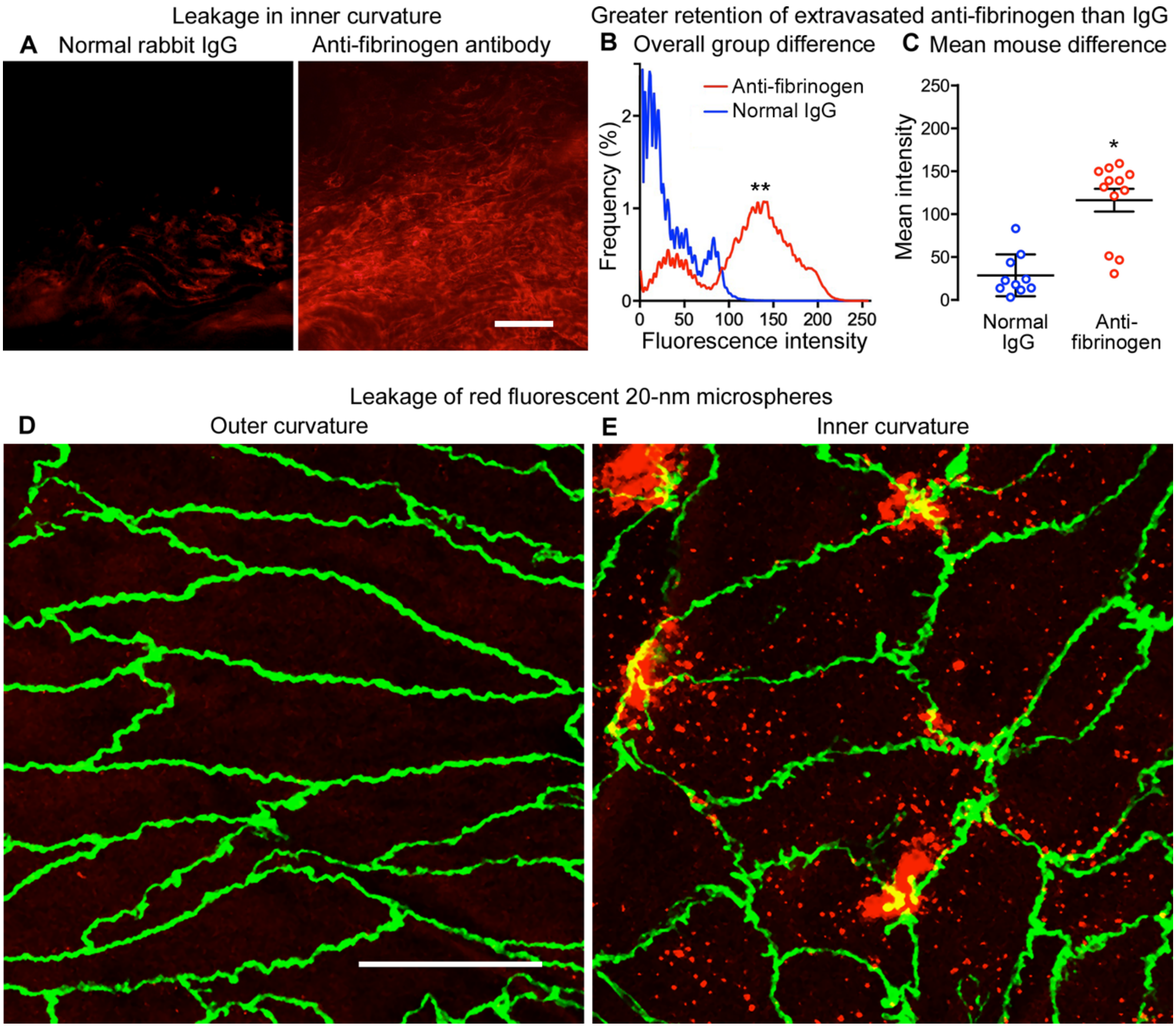

Appendix Figure S4. Validation of method for leakage measurement. (A) Fluorescence microscopic images comparing intensity of extravasated normal rabbit IgG or anti-fibrinogen antibody in inner curvature of aortic arch of wild-type mice. **(B)** Comparison of distribution of fluorescence intensities of normal rabbit IgG and anti-fibrinogen antibody in inner curvature. (**C**) Mean fluorescence intensity in each mouse for data in B. Mean ± SEM, n = 10-12 mice/group. **(D-E)** Confocal microscopic images comparing little extravasation of 20-nm red fluorescent microspheres in the outer curvature in D to focal sites of leakage in the inner curvature in E of the same wild-type mouse. The brightest patches of microspheres are near endothelial cell junctions. VE-cadherin (green). *$P < 0.05$, **$P < 0.01$, by Kolmogorov-Smirnov two-sample test in B and Student's *t*-test in C. Scale bars: 100 µm in A, 20 µm in D and E.

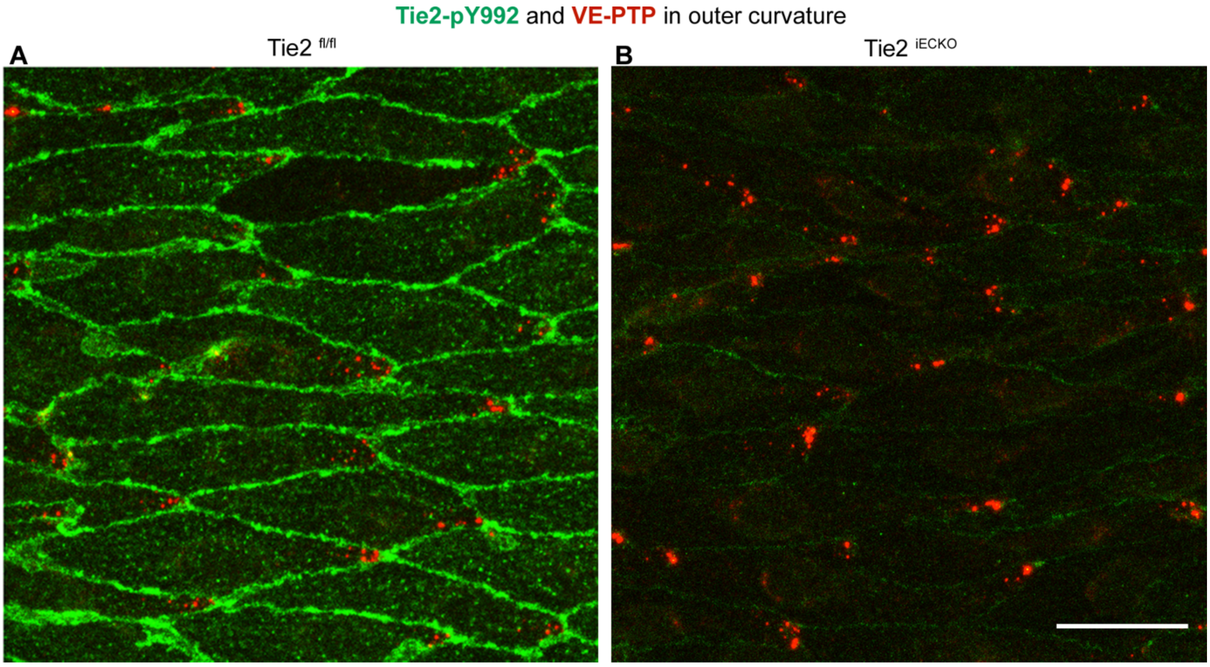

Appendix Figure S5. Specificity of Tie2-pY992 antibodies. (A-B) Comparison of staining for pY992-Tie2 of aortic endothelial cells in the outer curvature of Tie2$^{fl/fl}$ and Tie2^{iECKO} mice, as indicated. Scale bar: 20µm.

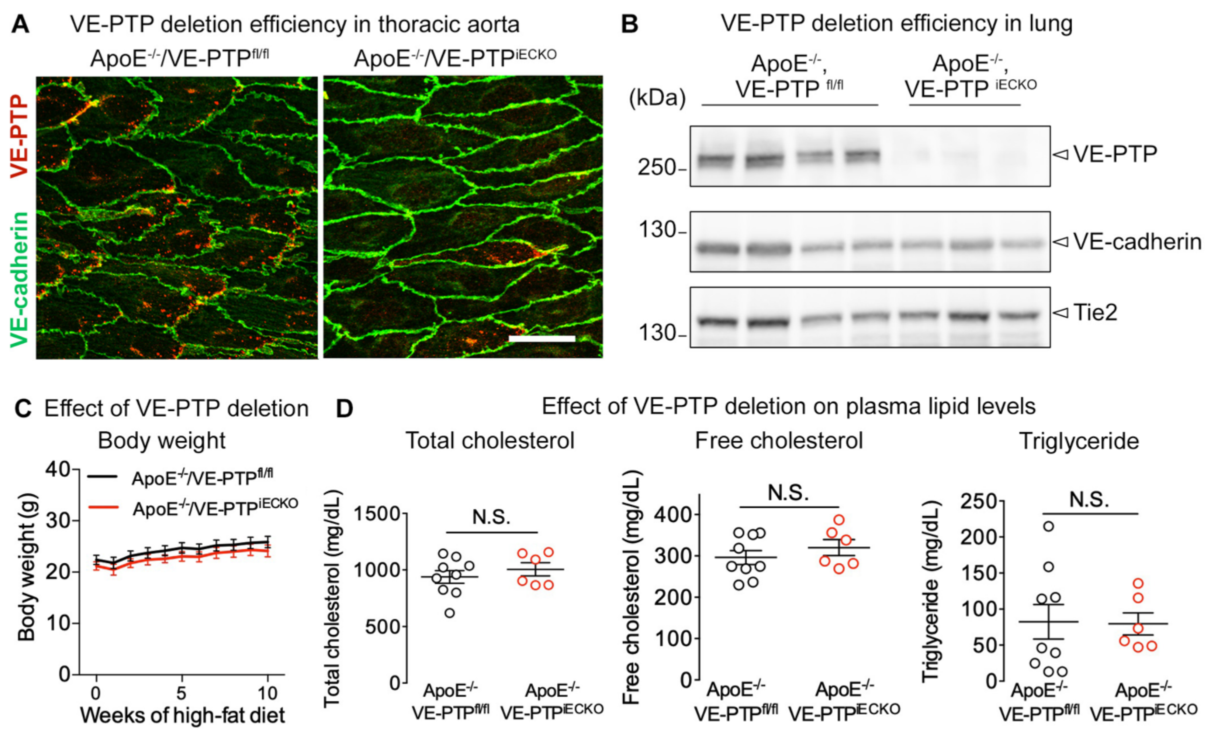

Appendix Figure S6. VE-PTP deletion in ApoE$^{-/-}$ mice without changes in body weight and plasma lipids. (A) Contrasting amounts of VE-PTP (red) staining in thoracic aorta of control ApoE$^{-/-}$/VE-PTP$^{fl/fl}$ mouse and ApoE$^{-/-}$/VE-PTP^{iECKO} mouse, in which VE-PTP was deleted in endothelial cells. VE-cadherin (green). **(B)** Confirmation of VE-PTP deletion by VE-PTP protein in lung lysates of control ApoE$^{-/-}$/VE-PTP$^{fl/fl}$ mice and ApoE$^{-/-}$/VE-PTP^{iECKO} mice. **(C-D)** No significant difference in body weight of ApoE$^{-/-}$/VE-PTP$^{fl/fl}$ mice (n = 18 mice) and ApoE$^{-/-}$/VE-PTP^{iECKO} mice (n = 13 mice) in C or in plasma lipids in ApoE$^{-/-}$/VE-PTP$^{fl/fl}$ mice (n = 9 mice) and ApoE$^{-/-}$/VE-PTP^{iECKO} mice (n = 6 mice) in D. Mean ± SEM. N.S. not significant, by Welch's *t*-test in D. Scale bar: 20 μm.

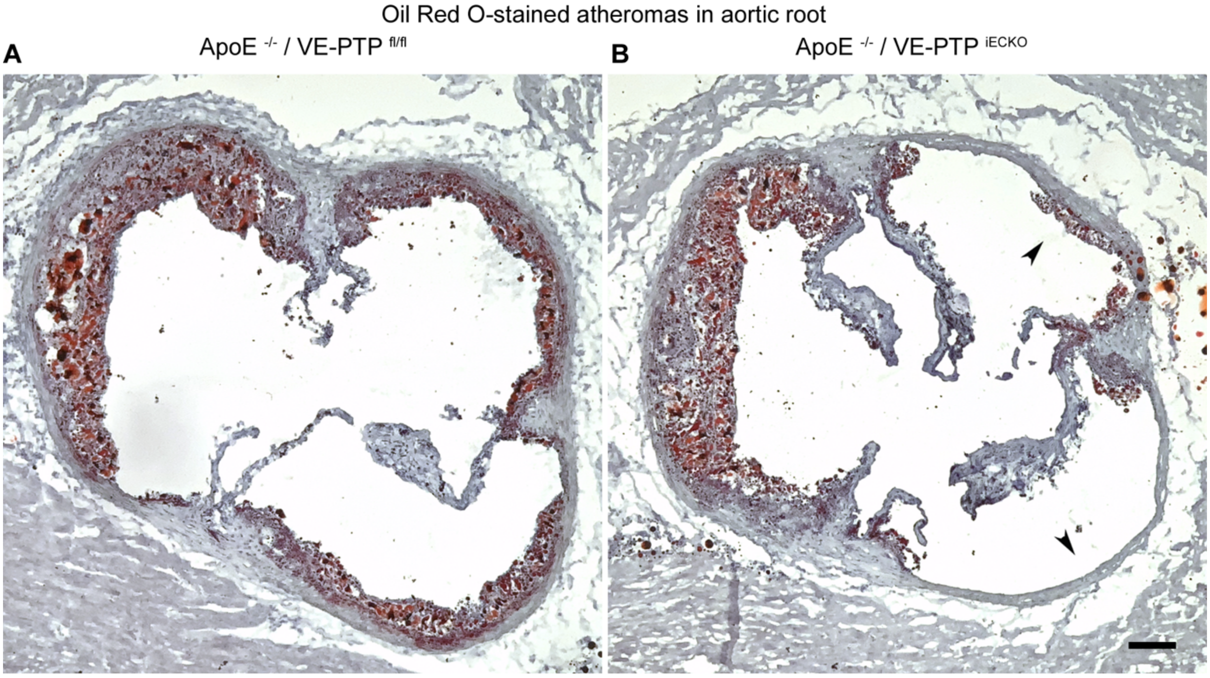

Appendix Figure S7. Oil Red O staining of atheromas in the aortic root. (A) ApoE$^{-/-}$/VE-PTP$^{fl/fl}$ mice and **(B)** ApoE$^{-/-}$/VE-PTP^{iECKO} mice were fed for 10 weeks with a high fat diet followed by preparing 8 µm cryostat sections of the aortic root in the plane of the aortic valve and staining with Oil Red O. Scale bar 100 µm.

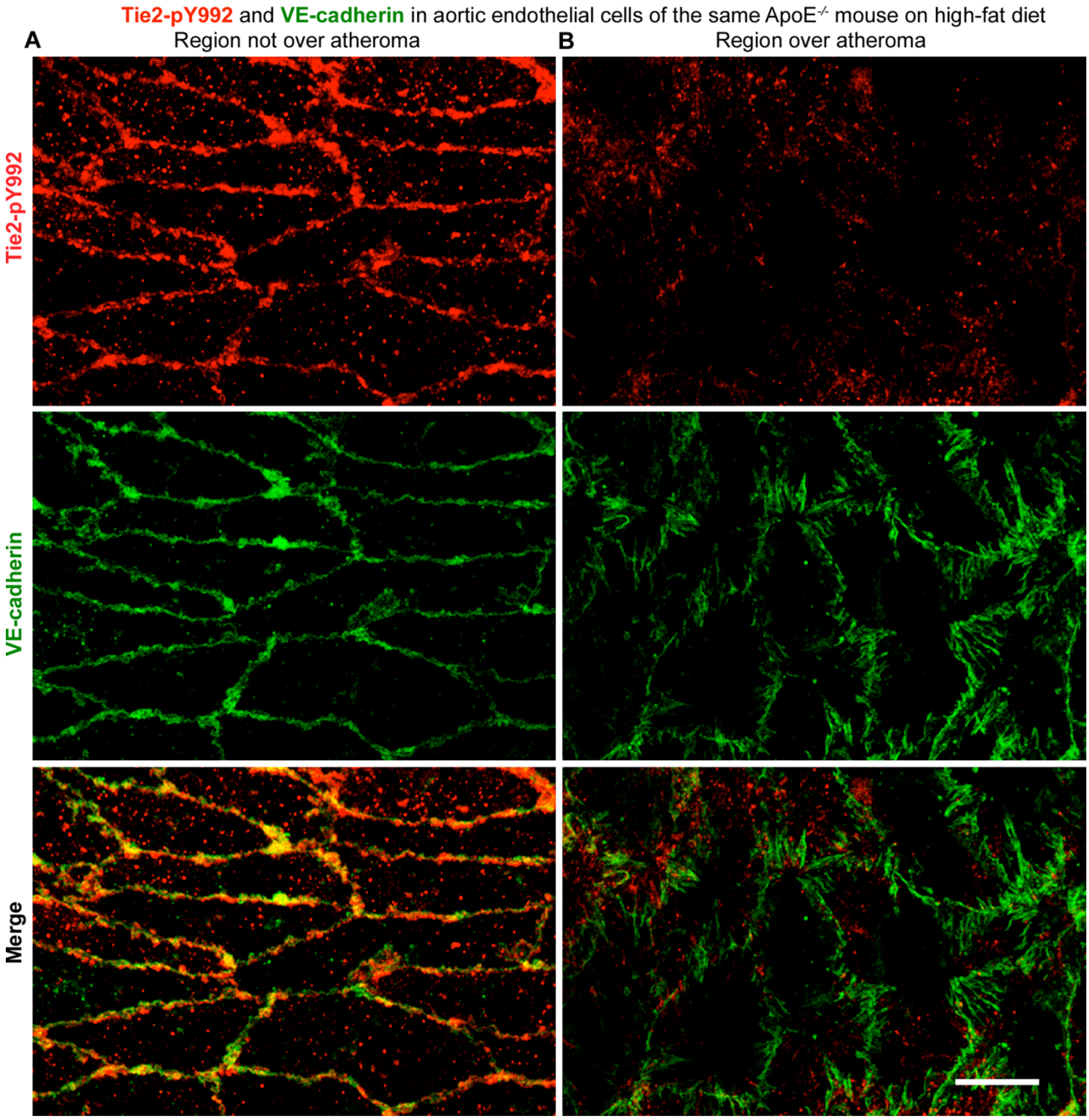

Appendix Figure S8. Contrasting staining of Tie2-pY992 and VE-cadherin at endothelial cells of normal region and over atheroma. (A-B) Strong staining for Tie2-pY992 in endothelial cells of a normal region of aortic arch in A compared to weak staining in endothelial cells over an atheroma in B in the aortic arch of the same ApoE$^{-/-}$ mouse on high-fat diet. VE-cadherin is also strikingly different, where staining delineates roughly linear cell borders in the normal region in A but has a zig-zag pattern in B that reflects the complex jagged contour of endothelial cell borders over the atheroma. Scale bar: 10 µm.

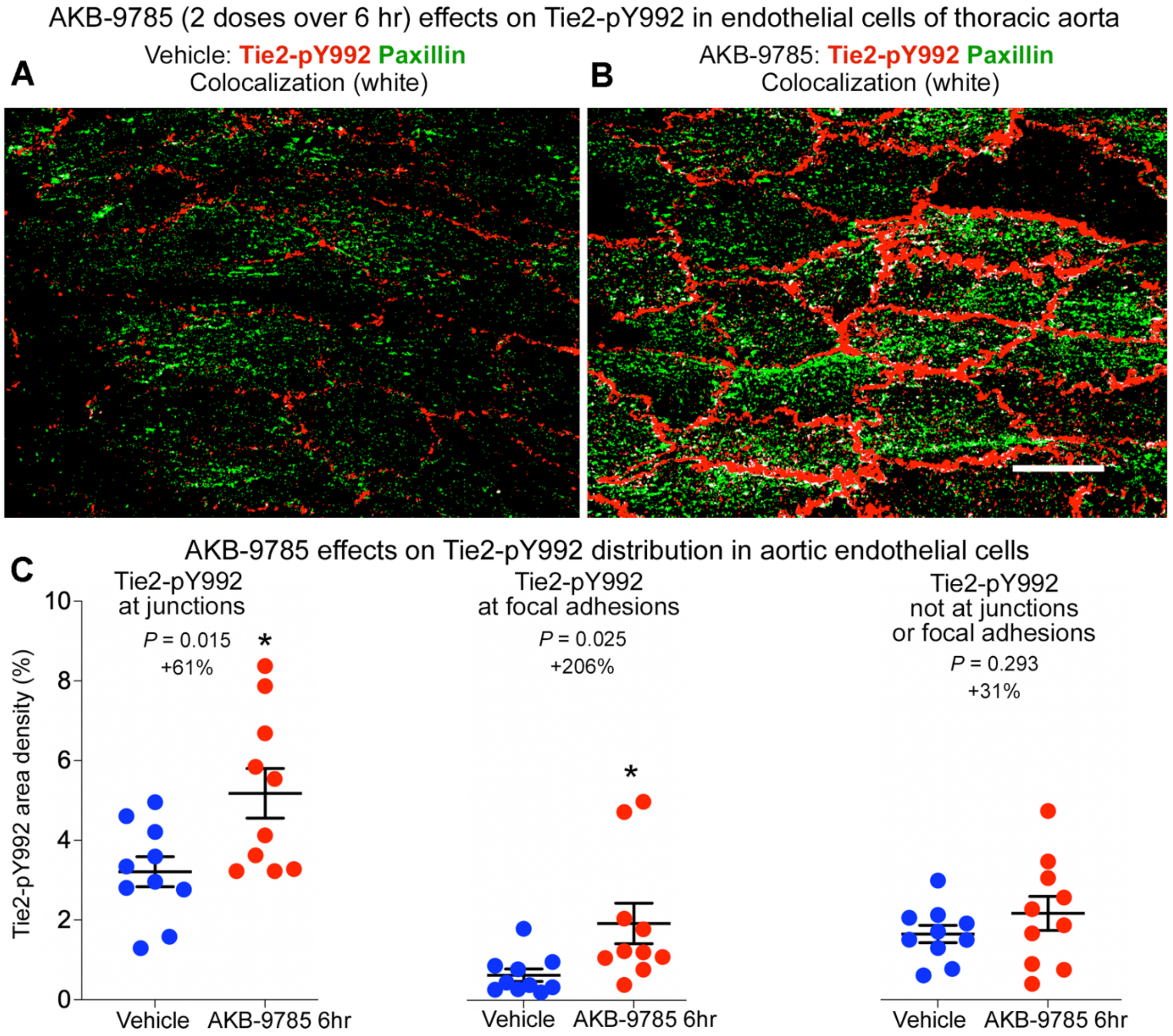

Appendix Figure S9. VE-PTP inhibitor AKB-9785 increases Tie2-pY992 at both cellular junctions and focal adhesions. (A) Mice were treated subcutaneously twice with vehicle or **(B)** with AKB-9785 over a period of 6 h, followed by staining of the thoracic aorta endothelium by antibodies against Tie2-pY992 (red) and paxillin (green). **(C)** Tie2-pY992 staining area density was quantified at endothelial junctions (left), at paxillin stained focal adhesions (middle) and at sites outside these areas (right), for vehicle treated (blue) and AKB-9785 treated (red) mice. Mean ± SEM, n = 10 /group, *P < 0.05 by unpaired Student's t-test in C

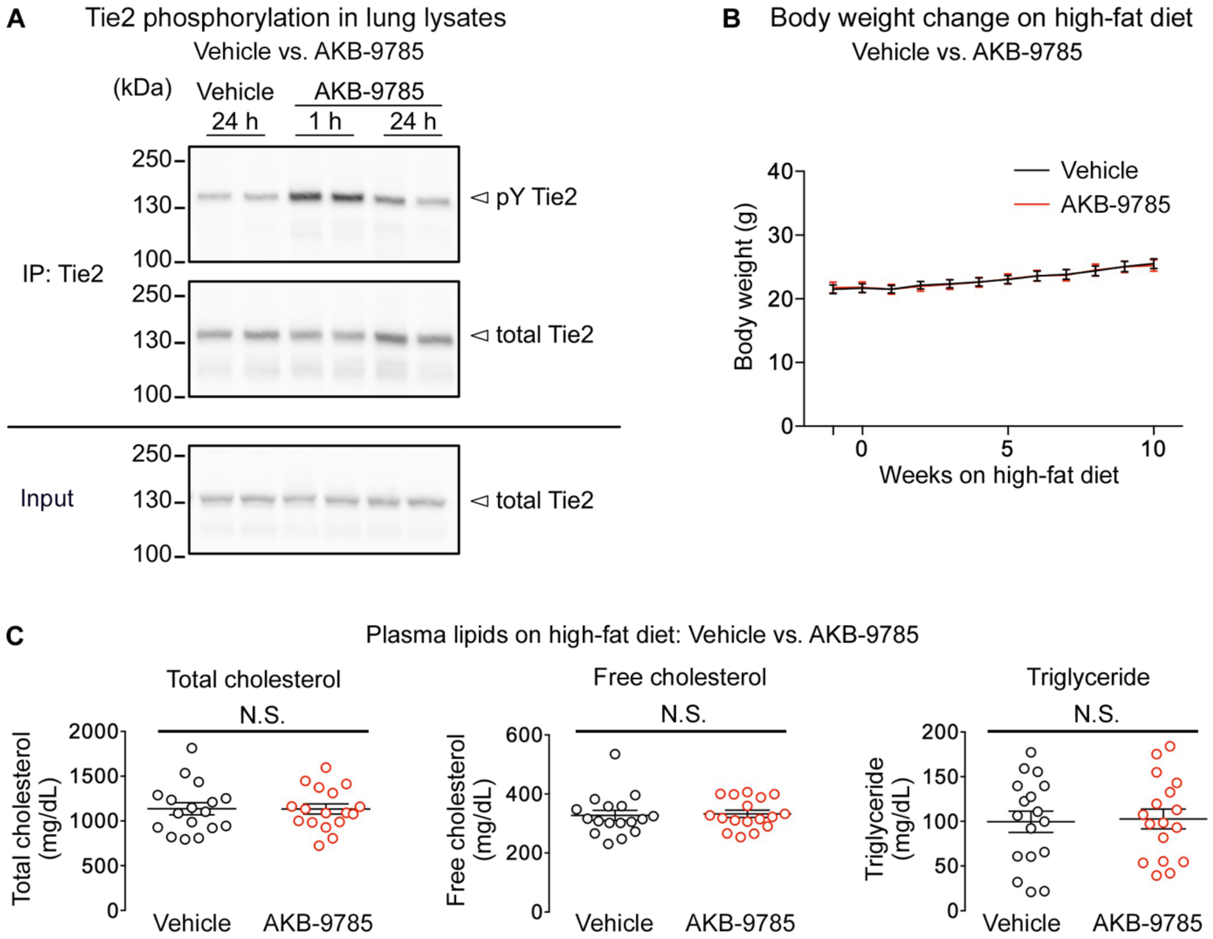

Appendix Figure S10. Increased Tie2 phosphorylation after AKB-9785 without changes in body weight or plasma lipids. (A) Phosphorylation of Tie2 in lung lysates of wild-type mice at 1 and 24 h after a single subcutaneous dose of vehicle or AKB-9785 (30 mg/kg, 100 µL of 10 mM to 20 g mice) showing a peak at 1 h after AKB-9785. Immunoprecipitates (IP) or lung lysates were immunoblotted for phosphotyrosine (antibody 4G10) or total Tie2. **(B)** Similar body weight of ApoE$^{-/-}$ mice on high-fat diet after daily treatment with vehicle or AKB-9785. **(C)** Absence of difference in plasma lipids in ApoE$^{-/-}$ mice after treatment with vehicle or AKB-9785. Mean ± SEM, n = 17 mice/group, N.S. not significant, by Welch's *t*-test in C.
