# Supplementary material for: Shear stress control of vascular leaks and atheromas through Tie2 activation by VE‐PTP sequestration
Source: EMBO Mol Med. 2023 Feb 6;15(4):e16128. doi: 10.15252/emmm.202216128 (PMC10086590; doi:10.15252/emmm.202216128)
Supplement: Supplementary file 2 — Expanded View Figures PDF [file EMMM-15-e16128-s006.pdf]

Expanded View Figures

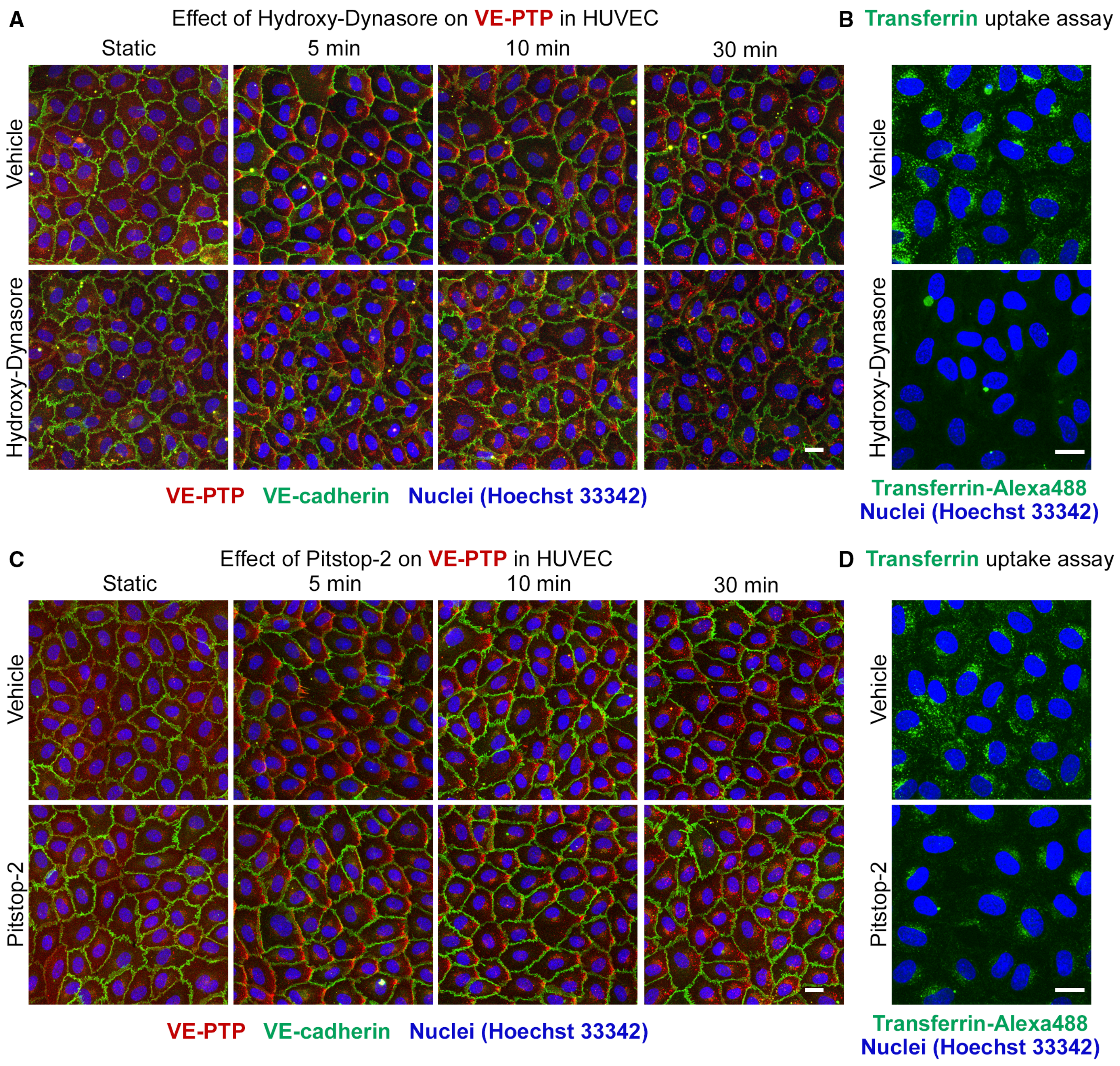

Figure EV1. Dynamin and Clathrin inhibitors do not affect internalization of VE-PTP under shear stress.

A HUVECs were treated either with DMSO (vehicle) or with Hydroxy-Dynasore at 100 μM for 1 h followed by exposure to shear stress of 15 dyn/cm^2 for 5 to 30 min. Subsequently, cells were fixed, permeabilized, and stained for VE-PTP (red), VE-cadherin (green) and for nuclei (blue).

B HUVECs were treated with vehicle or Hydroxy-Dynasore as in (A), followed by incubation under static conditions with transferrin-Alexa 488 (green) at 20 μg/ml for 15 min. Non-internalized transferrin was removed with acetic buffer pH 2.0.

C, D Similar as (A and B), except that Hydroxy-Dynasore was replaced by Pitstop-2 at 30 μM.

Data information: Scale bars: 20 μm.

 © 2023 The Authors

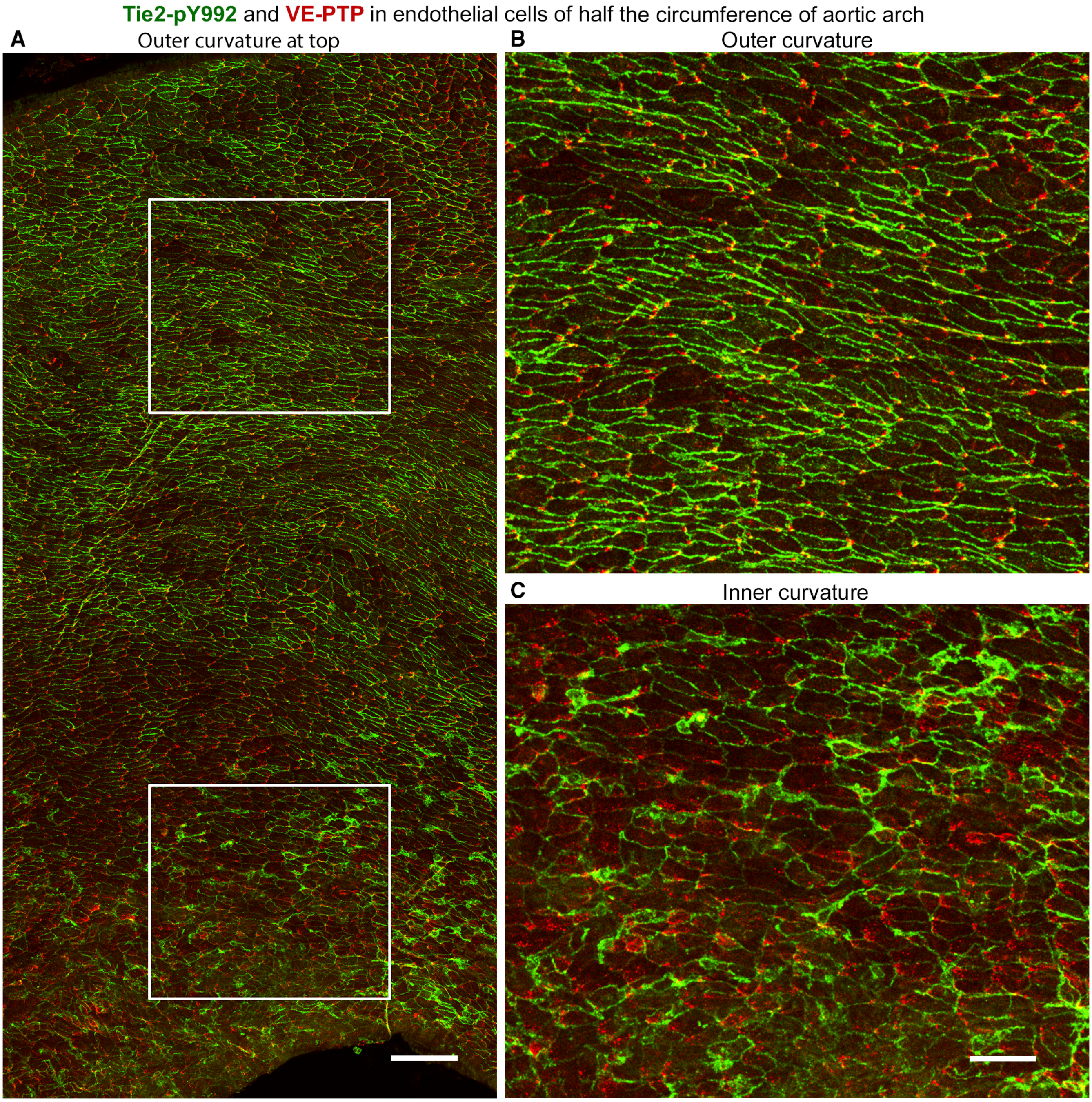

Figure EV2. Regional differences in VE-PTP and Tie2-pY992 staining in aortic arch.

A Low magnification confocal microscopic tile-scan image of half the circumference of bisected aortic arch of normal wild-type mouse. Outer curvature is at the top and inner curvature is at the bottom. VE-PTP (red) and Tie2-pY992 (green).

B, C Regions in white boxes in (A) are enlarged to show striking differences in VE-PTP and Tie2-pY992 staining in the outer curvature in (B) and inner curvature in (C). Regional heterogeneity in VE-PTP and Tie2-pY992 staining is also present within the outer curvature and even more so within the inner curvature. Net direction of blood flow is left to right.

Data information: Scale bars: 100 µm in (A), 50 µm in (B and C).

© 2023 The Authors

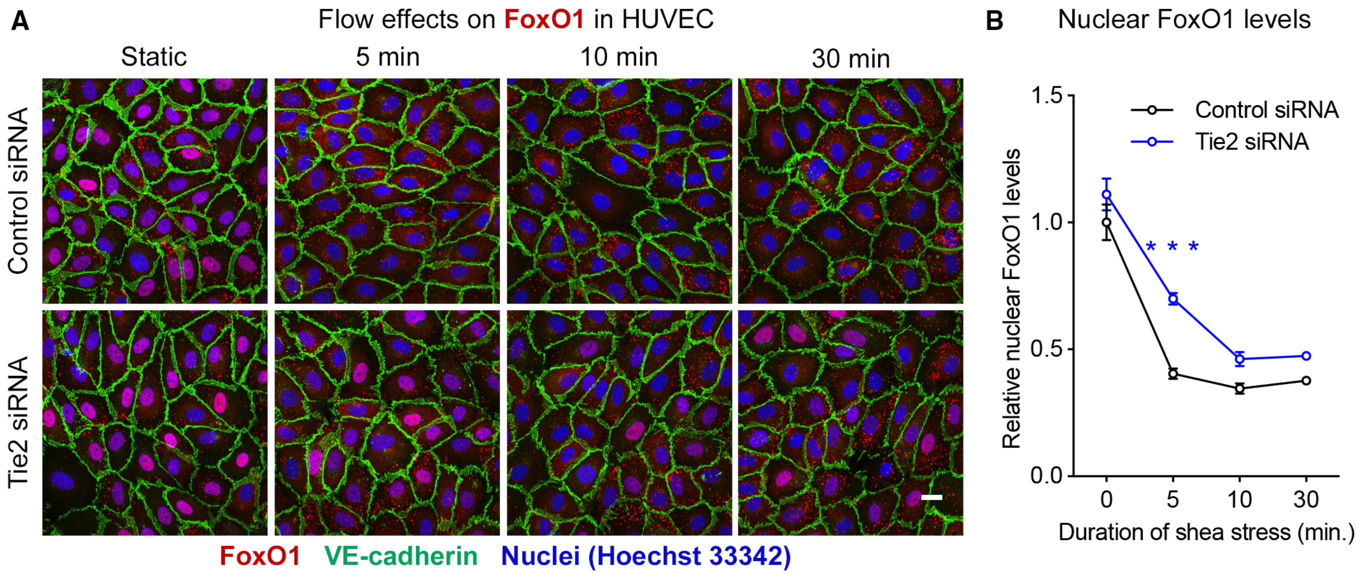

Figure EV3. Tie2 is required for shear stress-induced export of nuclear FoxO1.

A HUVECs treated either with control siRNA or Tie2 siRNA were exposed to 15 dyn/cm^2 shear stress for 5 to 30 min, followed by fixation, permeabilization, and staining for FoxO1 (red), VE-cadherin (green) and nuclei (blue). Scale bar: 20 µm.

B Amount of nuclear FoxO1 staining. Mean ± SEM, $n = 6$ cultures on independent flow chamber lanes. ***$P < 0.001$, by Bonferroni's test. $P = 0.17$ (0 min), 0.00000090 (5 min), 0.14 (10 min) and 0.31 (30 min).

 © 2023 The Authors

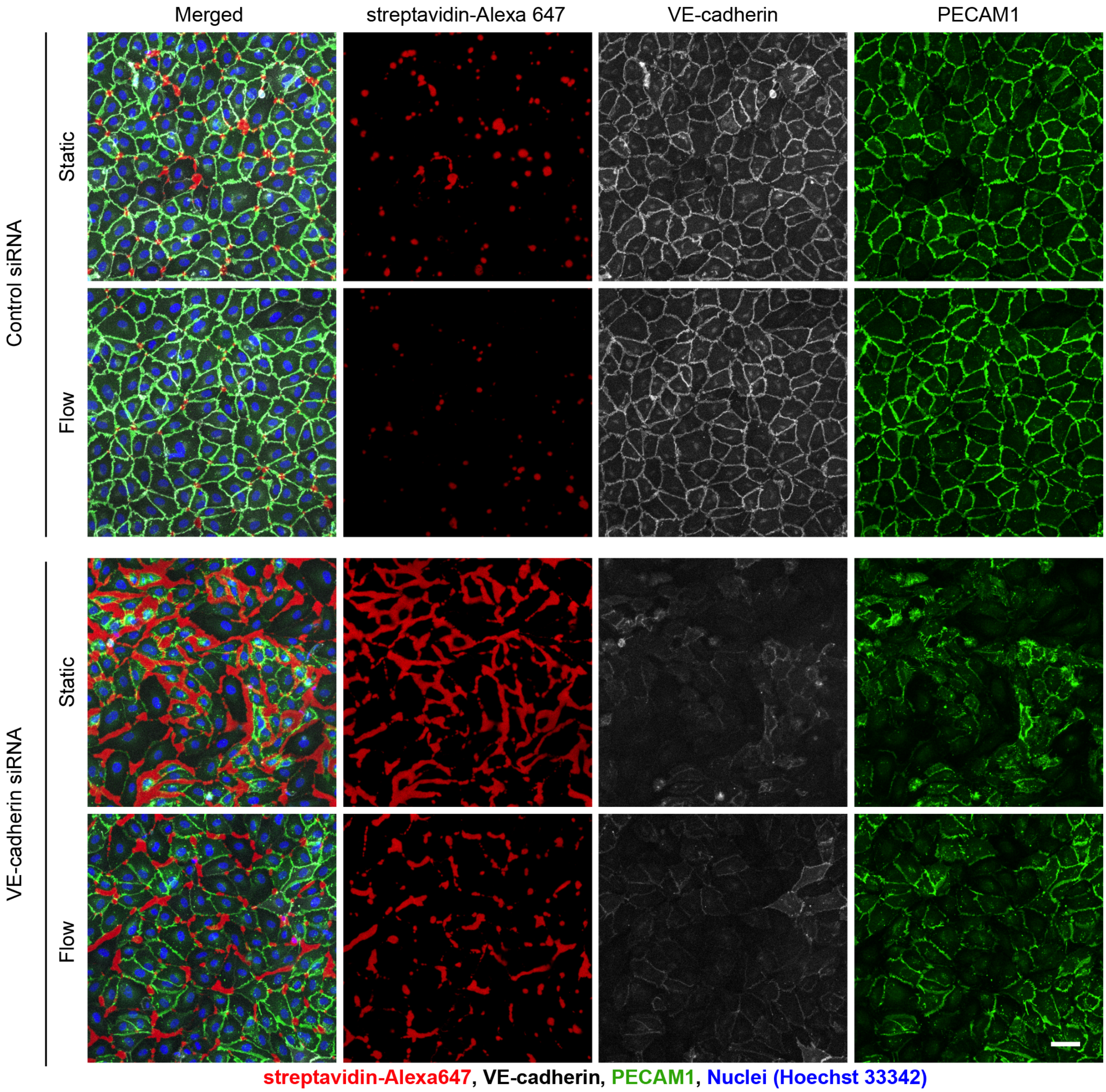

Figure EV4. Flow-inducedleakage reduction in HUVEC unchanged by VE-cadherin siRNA.

HUVECs cultured on biotinylated gelatin were treated either with control siRNA, or VE-cadherin siRNA, then exposed to 15 dyn/cm^2 shear stress for 30 min followed by 3 min incubation with streptavidin-Alexa647 (red), washing, fixation and staining for VE-cadherin (gray) and PECAM1 (green). The VE-cadherin siRNA data in Fig 6E are included in this figure. Scale bar: 50 μm.

© 2023 The Authors

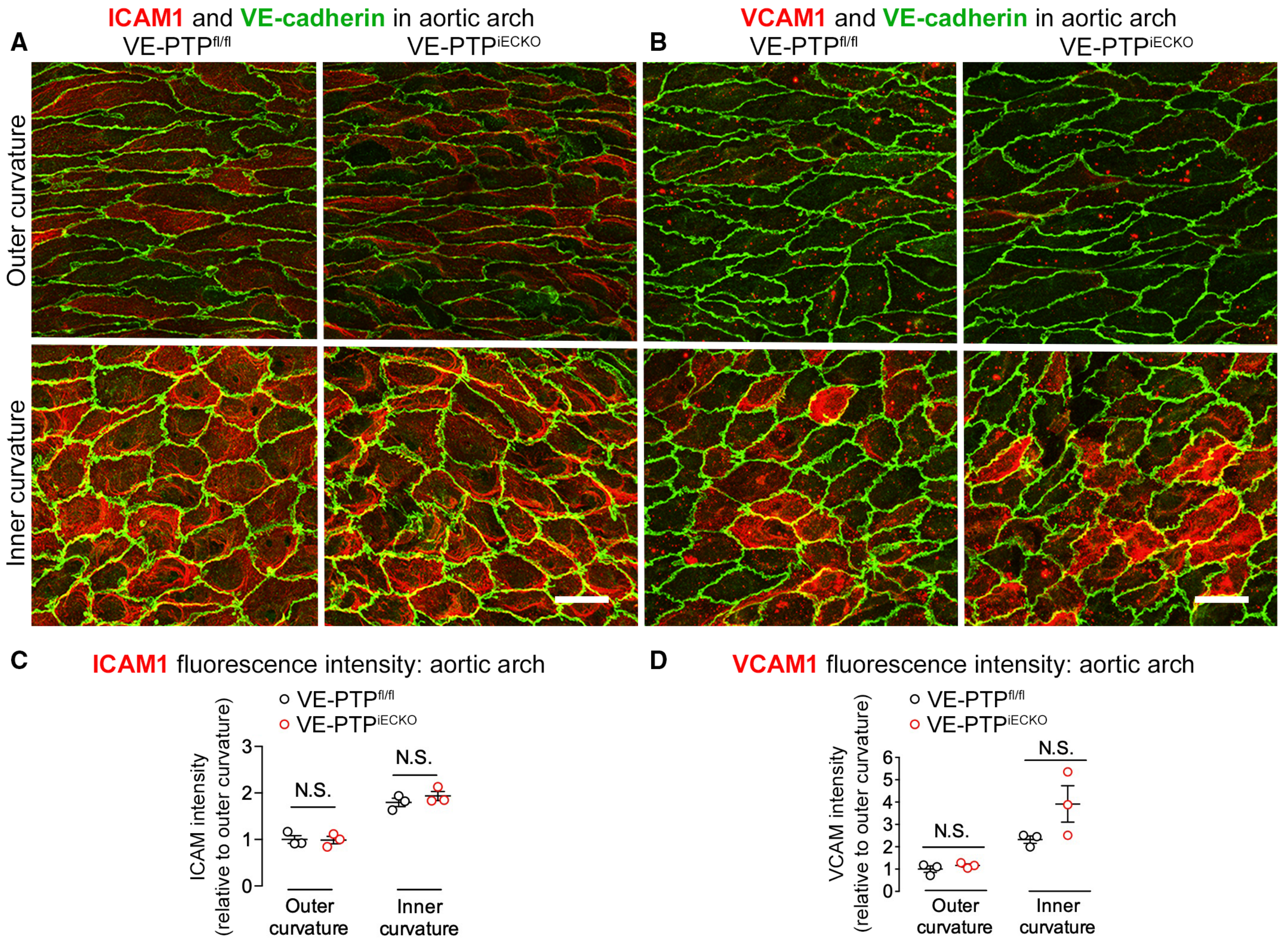

Figure EV5. No change in ICAM1 or VCAM1 expression after VE-PTP gene deletion.

A, B Weaker staining for ICAM1 in (A) and VCAM1 in (B) in outer curvature (top row) than in inner curvature (bottom row) of VE-PTP$^{fl/fl}$ mice and VE-PTP^{iECKO} mice. Deletion of VE-PTP had no apparent effect on ICAM1 or VCAM1 staining in either location. Scale bars: 20 μm.

C, D Measurements of mean fluorescence intensity confirmed stronger staining for ICAM1 and VCAM1 in the inner curvature but did not detect any significant difference between VE-PTP$^{fl/fl}$ and VE-PTP^{iECKO} mice. Mean ± SEM, $n = 3$ mice/group. P values were determined with Bonferroni test in (C and D). N.S. not significant. $P = 0.99$ (outer curvature) and 0.60 (inner curvature) in (C). $P = 0.99$ (outer curvature) and 0.057 (inner curvature) in (D).

© 2023 The Authors
