# Supplementary figures and images for: Shear stress control of vascular leaks and atheromas through Tie2 activation by VE‐PTP sequestration
Source: EMBO Mol Med. 2023 Feb 6;15(4):e16128. doi: 10.15252/emmm.202216128 (PMC10086590; doi:10.15252/emmm.202216128)

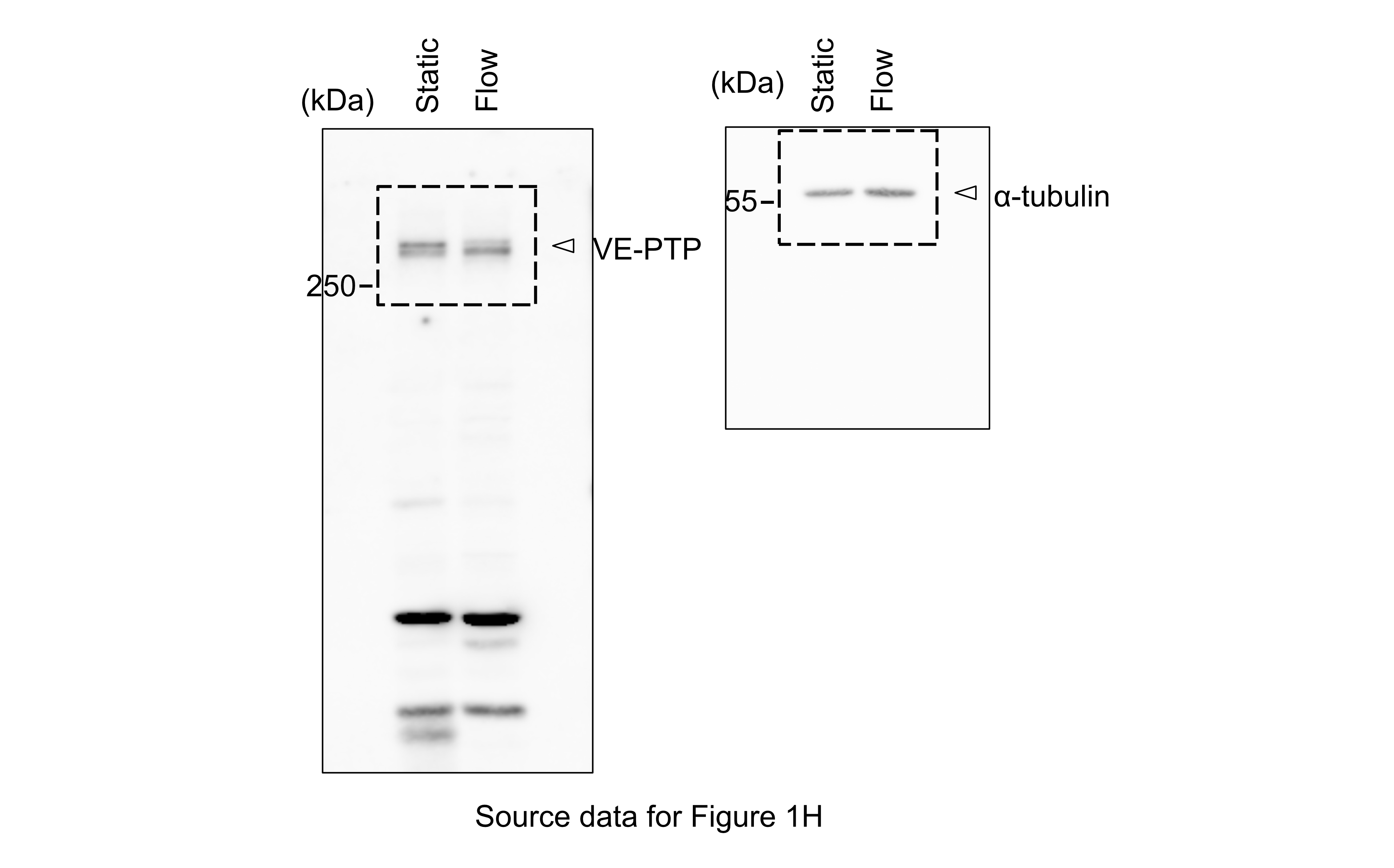

Supplement: Supplementary file 4 — Source Data for Figure 1 [file EMMM-15-e16128-s002.tif]

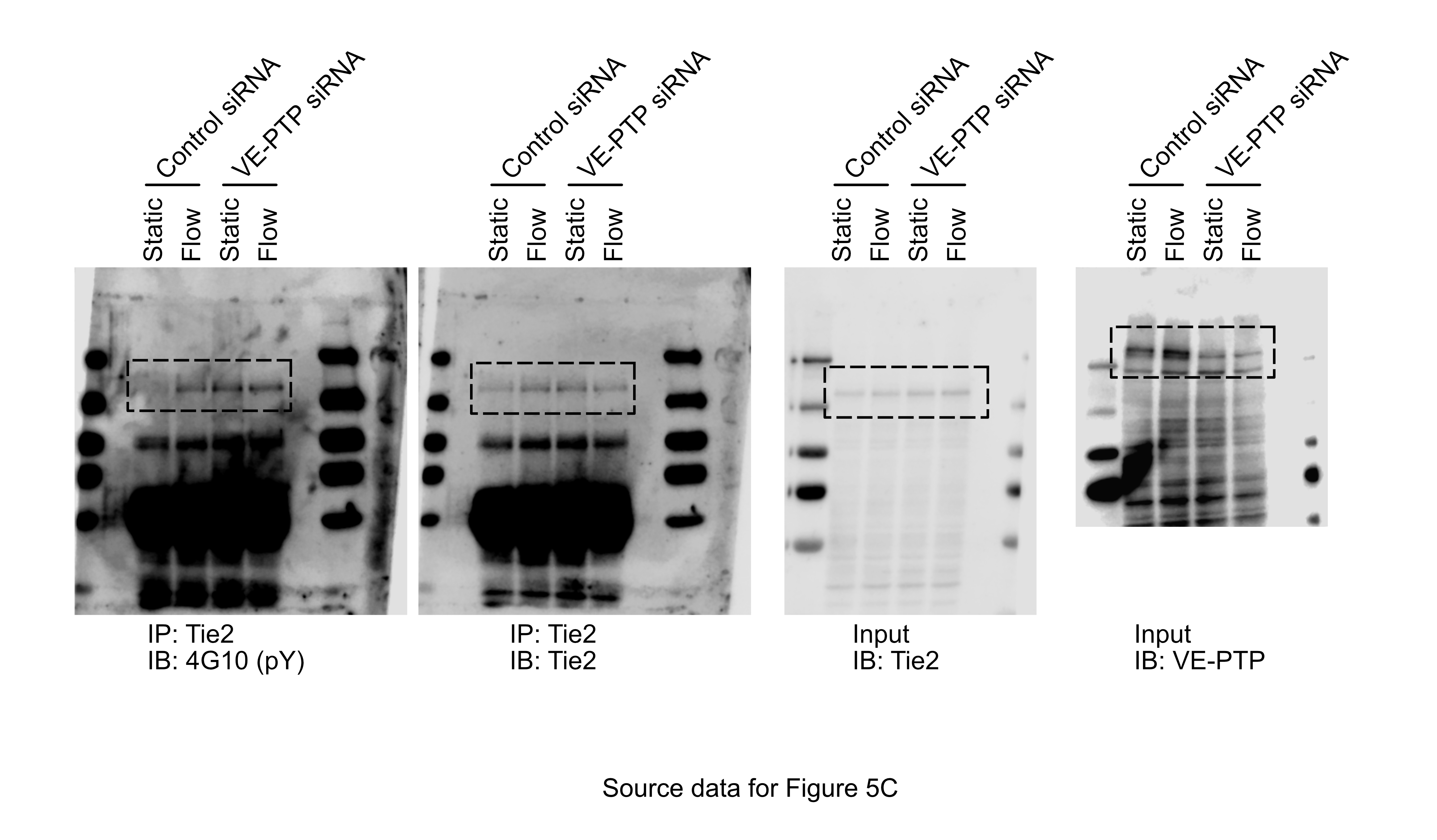

Supplement: Supplementary file 5 — Source Data for Figure 5 [file EMMM-15-e16128-s001.tif]
