# Supplementary material for: Shear stress control of vascular leaks and atheromas through Tie2 activation by VE‐PTP sequestration
Source: EMBO Mol Med. 2023 Feb 6;15(4):e16128. doi: 10.15252/emmm.202216128 (PMC10086590; doi:10.15252/emmm.202216128)
Supplement: Supplementary file 6 [file EMMM-15-e16128-s004.pdf]

EMBO Molecular Medicine

Shear stress control of vascular leaks and atheromas through Tie2 activation by VE-PTP sequestration

Keisuke Shirakura, Peter Baluk, Astrid Nottebaum, Ute Ipe, Kevin Peters, Donald McDonald, and Dietmar Vestweber
DOI: 10.15252/emmm.202216128

Corresponding authors: Dietmar Vestweber (vestweb@mpi-muenster.mpg.de) , Donald McDonald (donald.mcdonald@ucsf.edu)

Review Timeline:

Submission Date:	5th Apr 22
Editorial Decision:	4th May 22
Revision Received:	12th Nov 22
Editorial Decision:	7th Dec 22
Revision Received:	19th Dec 22
Accepted:	11th Jan 23

Editor: Lise Roth

Transaction Report:

(Note: With the exception of the correction of typographical or spelling errors that could be a source of ambiguity, letters and reports are not edited. Depending on transfer agreements, referee reports obtained elsewhere may or may not be included in this compilation. Referee reports are anonymous unless the Referee chooses to sign their reports.)

4th May 2022

Dear Prof. Vestweber,

Thank you for submitting your manuscript to EMBO Molecular Medicine, and please accept my apologies for the delay in getting back to you, which is due to the fact that one referee needed more time to provide his/her report. We have now heard back from the 4 referees who agreed to evaluate your manuscript.

As you will see from the reports below, the referees provide rather similar recommendations. They recognize the potential interest of your findings and appreciate the quality of the in vivo imaging. However, they also agree that the in vitro mechanistic results remain preliminary and unconvincing.

Given the extent of revisions required and considering that at EMBO Press we encourage one round of revisions only in a reasonable time frame, I am afraid I see little choice but to return the manuscript to you at this point with the decision that we cannot offer to publish it.

However, given the interest of your findings, we would consider a new manuscript if at some point in the near future you would obtain data that would convincingly strengthen the mechanistic aspect of your work.

I thank you again for your interest in EMBO Molecular Medicine and I am sorry that I could not bring better news. I hope that the referees' comments are helpful in your continued work in this area.

With kind regards,

Lise Roth

Lise Roth
Senior Editor
EMBO Molecular Medicine

***** Reviewer's comments *****

Referee #1 (Remarks for Author):

This is an interesting manuscript that described the dynamics of VE-PTP in vivo and its further contribution to the barrier function in physiological and pathological conditions.
Experiments are well executed and data are compelling.
There are some concerns and points that need further clarifications:
- Are VE-cadherin and VE-PTP co-internalized? Instead, are they internalized via different routes (similarly to VEGFR2)? Where does Tie2 is trafficking?
- At which step does exactly VE-cadherin contribute to the described mechanisms (ie is there a hierarchy between VE-cadherin and VE-PTP)? Is VE-cadherin the functional endpoint of VE-PTP modulation of the endothelial barrier? Alternatively, is VE-cadherin interaction with VE-PTP involved?
- Is Tie2 activation/phosphorylation corresponding to its steady-state? Can this VE-PTP-dependent pathway be modulated by angiopoietin and/or VEGF?

Referee #2 (Comments on Novelty/Model System for Author):

This M/S is providing nice in vivo observations which nevertheless are not corroborated with the few experiments performed on endothelial cells in vitro. There is thus a discrepancy between the two parts of the paper which thus overfall fails to provide a clear mechanism.

Referee #2 (Remarks for Author):

TIE2/VE-PTP review

The present study sought to determine the contribution of VE-PTP and Tie2 activation to effects of shear stress on endothelial barrier function and atherogenesis. The approach was to compare VE-PTP redistribution, Tie2 phosphorylation, and macromolecular leakage in regions of mouse aorta known to have contrasting shear stress profiles and in cultured endothelial cells exposed to defined flow conditions. Similar approaches were used to determine the contribution of VE-PTP to leakage and atheroma formation in mice having genetic ablation of VE-PTP in endothelial cells, alone or in combination with apolipoprotein E gene deletion (ApoE-/-) and a high-fat diet.

Whereas some interesting observations are provided in the second part of the present M/S, it overall fails to provide clear mechanistic insights that could link the first part and the second part of the paper. Indeed, the relationship of downstream polarization of VE-PTP staining in endothelial cells to the blood flow pattern was determined by comparing staining in the aortic arch outer curvature, which has higher average shear, to staining in the inner curvature, where flow is disturbed and average shear is lower. The distribution of VE-PTP staining had dot-like polarization toward the downstream tip of endothelial cells in the outer curvature, but was dispersed in the inner curvature. Shirakura et al. then state that they found that exposure to average high stress led to downstream polarization and endocytosis of VE-PTP accompanied by tie2 activation at cell junctions. Figure 1G indeed shows downstream polarization of VE-PTP under flow conditions and figure 2C shows endocytosis of VE-PTP. Yet, this is already well known as demonstrated by Mantilidewi et al. in J. Biol. Chem 2014. Here, internalization is rather weak in response to flow as authors mention 'some tie2 is colocalized with endosome markers'. This should be quantified properly.

Then, the authors claim that tie2 is activated at cell junctions but this is not clearly demonstrated (Figure 5C). Instead, the authors use western-blot analyses (phosphor-Tyr) after Tie2 immunoprecipitation in order to show that Tie2 is phosphorylated under flow conditions. This experiment does not demonstrate tie2 activation at cell junctions. Furthermore, heterogenetiy in cell populations makes it difficult to link these findings to the in vivo situation as individual cell behaviors are not necesseraly reflected in population-based measurements.

In addition, Mantilidewi et al. demonstrated that redistribution of VE-PTP triggered by shear stress was promoted by integrin engagement of extracellular matrix ligands. In parallel, flow-induced relocation of laminin-associated integrins on the basal endothelial surface is also well appreciated (Béguin et al. Mol Cell Proteomics 2020). Here, the potential link between basal endothelial surface and Tie2 activation is not studied whereas there might be a link as ECM-anchored tie2 promotes assembly of integrin-mediated cell adhesions. Do the authors think that there could be a transition from trans-associated Tie2 to ECM-associated Tie 2. Could this be revealed by performing co-localization experiments with vincullin or p-FAK ? Is there any effect on Cytoskeleton remodeling ?

Could the authors observe correlation with Tie2 Y992 and nuclear FOXO-1 ?

What about Tie2 Y820 phosphorylation as VE-PTP inhibition activates FGD5 in two steps: by Tie-2, Rap-1 dependent junction recruitment and by phosphorylation of Y820 (Braun et al. EMBO Rep 2019).

What about the VE-PTP internalization process ? Is it ligand dependent or independent ?

It is also well known that other key regulators of endothelial cell permeability such as VE-cadherin and VEGF-R2 are also relevant VE-PTP substrates and it is also well known that VE-cadherin functions as an adaptor that interacts with VEGFRs and promotes VEGFR activation in flow. It might thus well be that the effect of flow on VE-PTP is also mediated via these mechanisms.

In conclusion, despite nice observations in vivo, mechanistic insights are not clear at all. Authors should focus on in vitro experiments (laminar vs. disturbed/oscillatory and high vs. low shear stress) on HUVEC and better quantify any link between VE-PTP localization/Tie2 activation and endothelial permeability in order to appreciate causality more than correlations. This is not the case in the present paper.

Minor comments:

- Conspicuous and conspicuously are used 8 times in the present M/S. Authors should better quantify experimental results appropriately instead.
- Last sentence page 11 is not clear.

Referee #3 (Comments on Novelty/Model System for Author):

Interesting but flawed study. The authors failed to consider a major molecular pathway taht would counteract the system they

are studying. The anatomic phenotype data are not convincing and raisers a host of questions.

Referee #3 (Remarks for Author):

The study by Shirakura et al. reports that differential aorta regions exposed to disturbed flow shear stress have increased baseline vascular leakage which in turn promotes atherosclerotic plaque formation. The authors link this phenotype to cellular redistribution of VE-PTP by linear vs. disturbed shear that leads to activation of Tie signaling that, in turn, reduces vascular leakage. The authors further demonstrate that VEPTP deletion (or inhibition) increases Tie2 phosphorylation in atheroprone regions and reduces vascular permeability/atheroma formation. Although the study proposes a novel mechanism contributing to atherosclerotic plaque formation, there are a number of important issues that decrease my enthusiasm for the study. Overall, images in the study are beautiful, almost a work of art, but the proposed molecular mechanism are sketchy and the claimed biological effect not all that convincing
1. The link between Tie2 and VE-TP has been long established and the concept that VE-PTP inhibition activates Tie signaling and contributes to decreased vascular leakage extensively reported including by a number of this manuscript's authors. The novelty here is the purported link to atherosclerosis. In this regard, there are clear differences in VE-PTP distribution in DSS vs. linear shear stress areas. The effect o9n leakage is also quite clear. Yet, the phenotypic effect of endothelial VE-PTP deletion is not that convincing. It seems that the differences in Oil-red-O stating are largely driven by 3-4 outliers in thr VEPTP KO group: two thirds (12 out of 18) of the KO mice who complete overlap in the ORO extent with control group (Fig 7B). The same applies to the extent of leakage (Fig 7D) Atheroma size (Fig 8E shows a similar extent of overlap). It's almost if there are two mice populations after VE-TP KO : those that are much worse and those that are basically unchanged. This get to the molecular mechanisms issues (see below)N
2. The analysis of atheromas is very unconvincing. This is not hwo it si normally done ion atherosclerosis literature. One needs to show histological sections from multiple areas (aortic root, brachiocephalic, ascending aorta) where atheromas are clearly visible with proper quantitative assessment of plaque area and inner core size. I should also note that the extent ORO staining is pretty low. I am not sure why. Finally, while the authors show convincing data that VEPTP deletion (or inhibition) completely restores Tie2 phosphorylation in the inner curvature. Yet atheroma size does not appear to be reduced in the inner aorta curvature but only in aortic arc branches (particularly brachiocephalic artery) and not by that much. I think it is important that the authors provide a separate quantification for the inner curvature vs. aortic arch branches for both VEPTP iECKO (fig. 7a) and AKB-9758 experiments (fig 8a). Given this discrepancy, more experimental evidence needs to be provided to support the proposed atheroprotective mechanism. The authors should characterize the aortic branches (e.g. brachiocephalic trunk) and evaluate Tie2 phosphorylation, permeability, VCAM/ICAM staining in this region in both VEPTP iECKO and AKB-9758 experiments.
3. Redistribution of VEPTP from dispersed to a polarized intracellular localization nicely correlates with increased Tie2 phosphorylation. I think that the causational link between these two events should be strengthen in vitro by addressing the following points:
- does exposure to high shear stress induce VEPTP downregulation over time? Let's say at 24hrs after exposure to high shear. This can be easily assessed in vitro by western blot analysis. It would be interesting to know since VEPTP staining in figure 1E seems overall higher in the inner curvature. Higher VEPTP expression in areas exposed to low-shear stress could lead to higher Tie2 dephosphorylation in addition to VEPTP redistribution across all cell surface

- Additionally, a confirmation of changes in VEPTP surface level should be performed by surface biotinylation and side-by-side western blot comparison of HUVEC in static conditions vs high shear stress after 30 minutes and 24 hrs.
- Fig. 1G nicely show that VEPTP become polarized after HUVEC are exposed to high laminar shear stress. Adding Tie2 phosphorylation staining (the pY992 antibody seems very good!) to this time course would strongly argue for a causational link. Since VEPTP redistribution happens quickly, increased pTie2 should be visible already at 5 minutes.
4. Molecular mechanisms. The authors exclusively focus on VE-PTP KO effect on Tie2 activation. But this VEPTP KO will also increase VEGFR2 activity that will increase permeability. The wto signals may well offset. This is likely why that when it happens (2/3 of the mice) overall effect sin ill and when it does not, you have the phenotype. Thus, VEGFR2 activation, Src activation and VE-cadherin phosphorylation need to be assessed in vitro and in vivo in the presence and absence of VEPTP KO and normal and abnormal shear. The data are not interpretable without this information
- VE-PTP inhibitor. The authors make much of the AKB-9785 VE-PTP inhibitor used in this study and suggest it could be a useful therapeutic agent. That seems highly doubtful. The drugs seems to have failed in TIME-2B study the authors mention that has never been published. I infer that from the fact that the company behind the drug no longer exists. I am rather skeptical that a systemic inhibition of VEPTP can be a good thing especially when carried out on a chronic basis as would be required for atherosclerosis therapy.
Other comments:
In Figure 1-B, VE-PTP seems highly expressed in some of the cells of the inner curvature and not in others, while in the text the authors described an overall lower level of VE-PTP expression in the inner side compared to the outer side. How would you explain this? Can you provide a picture at lower magnification?
In Figure 2-C and D the authors show by immunofluorescence the decrease of VE-PTP expression at the cell membrane and the increase of internalized VE-PTP. It would be good to confirm these data by showing biochemically the decrease of surface VE-PTP versus whole cell VE-PTP expression with increasing exposure time to laminar shear flow.
The authors suggest that Tie2 activation depends on VE-PTP internalization. To corroborate this the authors should test the expression of Tie2-pY992 and VE-PTP after inhibition of endocytosis in HUVEC under laminar flow.

In aortas VE-PTP is expressed at the downstream pole of ECs, however HUVEC cells exposed to laminar flow for 24 hours, do not express this strong VE-PTP polarization (supplemental figure2 and figure 1 G) how do you explain this difference?
Authors show a correlation between Tie2-pY992 expression and VE-PTP internalization. What is tie2-pY992 expression in HUVECs under static flow, when VE-PTP is less expressed?

Referee #4 (Comments on Novelty/Model System for Author):

The medical impact refers to the delivery regime used in the study. The medical impact would be higher with a more realistic dosing.

Referee #4 (Remarks for Author):

This study by Shirakura and colleagues, investigates the consequence of VE-PTP loss on the development of atherosclerosis in an animal model. Depletion is either genetically or with the VE-PTP inhibitor, AKB9785. They further investigate the mechanism of redistribution and loss in endothelial cells in athero-prone regions in the aorta and in in vitro models.
The study is well performed and the in vivo images especially are very impressive. However, I have a number of issues that require addressing

1. The authors need to address the mechanism underlying the inhibition of atheroma development. They show leak is inhibited. They show atheroma is inhibited by the drug or genetically, but no change in the lipid profiles. But what is happening here? Is there less lipid accumulation in the tissue? Or inflammatory infiltrate, even though they report no change in VCAM and ICAM on the endothelium? More detailed investigations into the progression of atherosclerosis is needed.
2. Therapeutically, delivery of AKB-9785 every day for 11 weeks is a mammoth delivery regime and one that is unlikely to be accepted. The authors need to present more realistic treatment options, even for proof-of-principle studies. A change in delivery regime may also be essential to determine the mode of action to inhibit atheroma development (Point 1 above). When can you intervene in the process and still get effects?
3. Mechanistically, the authors need to address what controls the redistribution of VE-PTP. Schwartz and colleagues have shown that Rho is regulated and is important in flow mediated alignment. Is VE-PTP redistribution also Rho regulated?
4. The endothelial cells align even in the absence of VE-PTP (Fig5A). Alignment is considered a protective mechanism of EC in the atheroprotective sites. Is there a link between alignment and loss of VE-PTP? If polarization of VE-PTP is inhibited, is alignment blocked? If alignment is blocked does VE-PTP redistribute, etc?
5. The authors talk about the selectivity of AKB-9778 for VE-PTP. Some comment on how 9778 and 9785 differ and the selectivity of AKB-9785 is needed.
6. The localization of VE-PTP to endosomes needs strengthening rather than only colocalization by single images. Some biochemical studies to give support and also perhaps inhibitor studies should be shown.
7. In the aorta, there are regions where the cells are aligned, VE-PTP is polarized and cells in the next region where there are aligned, and VE-PTP mostly is lost (eg Fig3A & B). This change in the level of expression of VE-PTP, does this equate to differences in the activation status of the cell? Perhaps using p65 of the NFkB complex may help?
8. Fig2C-localisation to the plasma membrane is not easy to see. A specific marker needs to be included.
9. Fig3E. The intensity of VE-Cadherin is so much stronger in the inner curvature and this doesn't fit with the loss of Tie2-pY992 and likely a weaker junction.
10. Supplementary Fig8A in lung is not discussed.
11. Is the anti-fibrinogen measurement of leak always paracellular?

As a service to authors, EMBO provides authors with the possibility to transfer a manuscript that one journal cannot offer to publish to another EMBO publication. The full manuscript and if applicable, reviewers reports are automatically sent to the receiving journal to allow for fast handling and a prompt decision on your manuscript. For more details of this service, and to transfer your manuscript to another EMBO title please click on Link Not Available

Please do not share this URL as it will give anyone who clicks it access to your account.

Detailed reply to reviewers:

We thank the reviewers for their positive and constructive comments that we have addressed below as follows:

Referee #1 (Remarks for Author):

1. *This is an interesting manuscript that described the dynamics of VE-PTP in vivo and its further contribution to the barrier function in physiological and pathological conditions. Experiments are well executed and data are compelling.*

2. *There are some concerns and points that need further clarifications:*
a. *Are VE-cadherin and VE-PTP co-internalized? Instead, are they internalized via different routes (similarly to VEGFR2)? Where does Tie2 is trafficking?*

We did not observe evidence for internalization of VE-cadherin upon exposure of endothelial cells to shear stress (Figure 1G and 2A). This indicates that shear-induced internalization of VE-PTP is independent of VE-cadherin. We also analyzed whether the subcellular localization of Tie2 would change due to shear stress. As shown below in **figure 1of this letter (see below)**, Tie2 expression at cell contacts was not reduced and we observed no signs for shear stress-induced Tie2 endocytosis. Thus, shear stress-induced VE-PTP internalization into cells left cell contact expression of VE-cadherin and Tie2 intact. Unspecific nuclear staining was due to antibody background signals (since it was also seen upon Tie2 silencing).

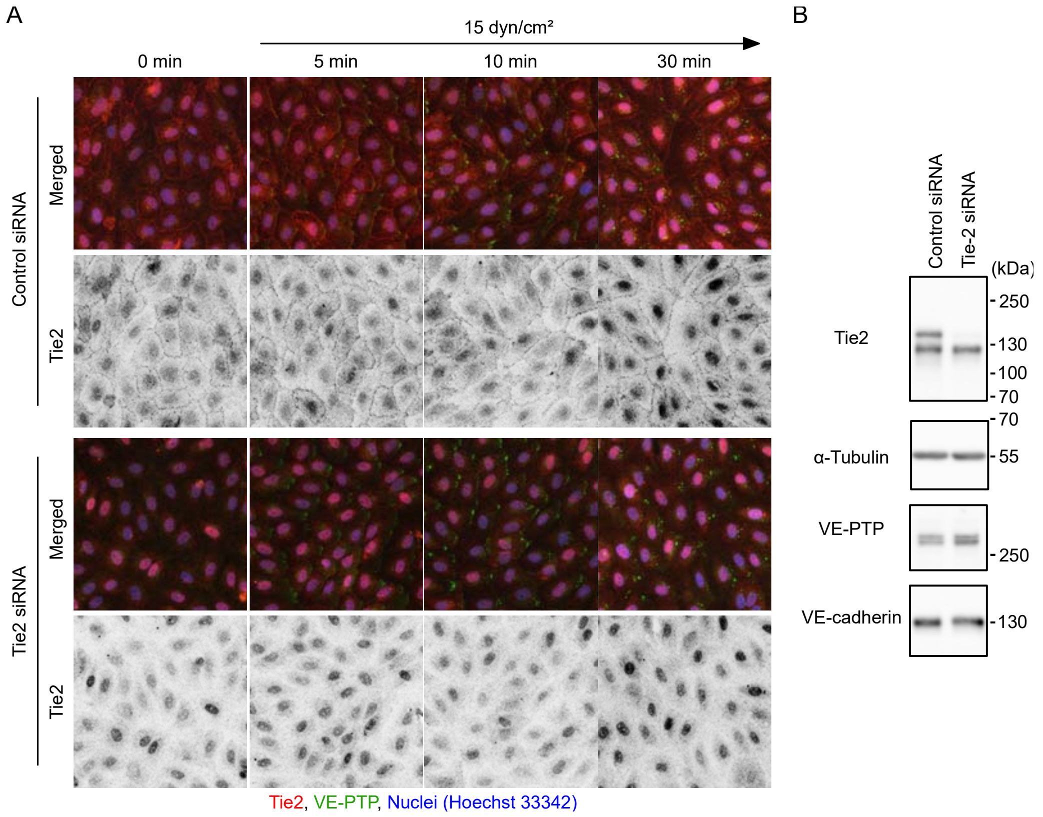

Figure 1 Shear stress does not alter Tie2 localization. HUVECs were exposed to 15 dyn/cm^2 of shear stress for the indicated times. The resulting cells were fixed and stained for Tie2 (red) and VE-PTP (green).

b. *At which step does exactly VE-cadherin contribute to the described mechanisms (ie is there a hierarchy between VE-cadherin and VE-PTP)? Is VE-cadherin the functional endpoint of VE-PTP modulation of the endothelial barrier? Alternatively, is VE-cadherin interaction with VE-PTP involved?*

We have shown previously that inhibition of VE-PTP stabilizes endothelial junctions *in vitro* and *in vivo* by triggering the activation of Tie2, an effect that was even detected in the absence of VE-cadherin (Frye et al. J Exp Med, 2015). These effects are supported by the strengthening of circumferential actin bundles and the inhibition of radial stress fibers (Braun et al., EMBO Rep. 2019).

We have now performed new experiments where we have analyzed the effects of VE-PTP and Tie2 silencing on shear stress-increased junction tightening. For this, we established a new permeability assay where we can visualize permeability for Streptavidin-Alexa647 through junctions of HUVEC monolayers. As shown in the **new parts E and F in Fig. 6 (page 14)**, both, silencing of VE-PTP and of Tie2, blocked the increase of endothelial barrier function induced by exposure of the cells to 30 min shear stress at 15 dyn/cm^2. Thus, VE-PTP and Tie2 are essential for flow-induced endothelial junction tightening. We also tested the relevance of VE-cadherin for this effect. Although we found that silencing of VE-cadherin by siRNA enhanced baseline permeability of the endothelial cell monolayer, the shear stress-induced stabilization of junctions was still observed despite the lack of VE-cadherin, as shown in **figure 2 of this letter (see below)**. These results demonstrate that shear stress-induced junction tightening depends on VE-PTP and Tie2, but not on VE-cadherin.

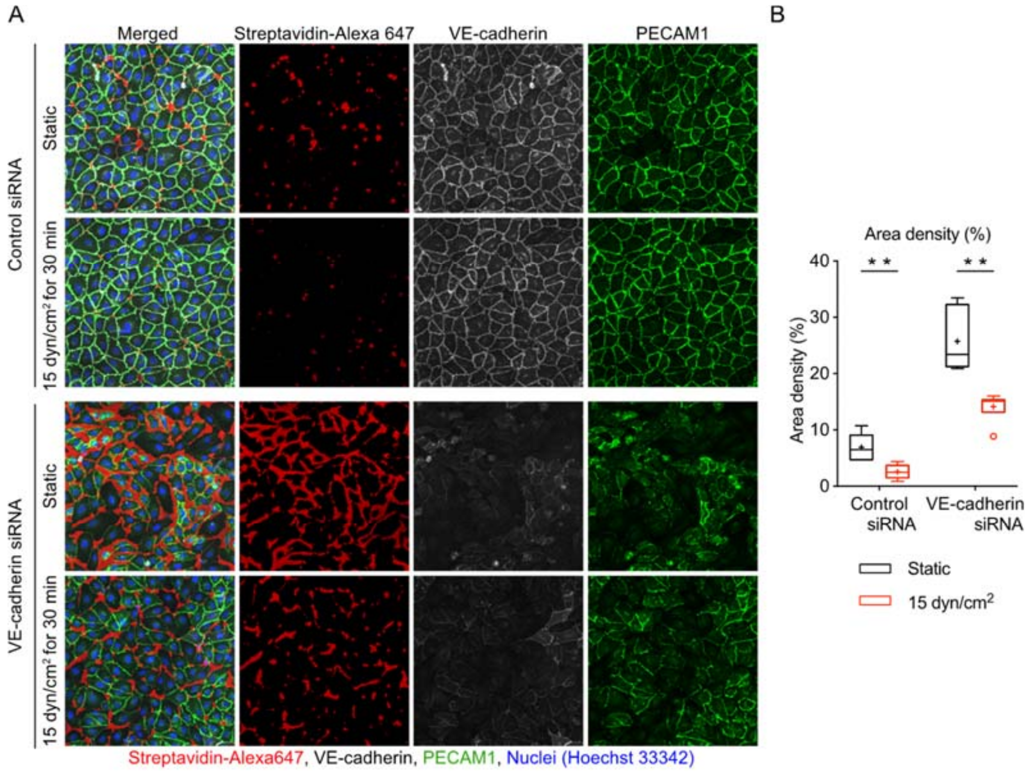

Figure 2 VE-cadherin is not essential for shear stress-mediated endothelial barrier stabilization. (A) HUVECs cultured on biotinylated gelatin were treated with control siRNA or VE-cadherin siRNA, followed by exposure to 15 dyn/cm^2 shear stress for 30 minutes. Subsequently, cells were incubated for 3 min with Streptavidin labeled with Alexa647 (Red), washed, fixed and stained for VE-cadherin (gray) and PECAM1 (green). **(B)** Quantification of streptavidin leakage area per image.

c. *Is Tie2 activation/phosphorylation corresponding to its steady-state? Can this VE-PTP-dependent pathway be modulated by angiopoietin and/or VEGF?*

It is not unlikely that Tie2 has some intrinsic kinase activity due to some level of dimerization even in the absence of agonists (personal communication Kurt Ballmer-Hofer, Paul Scherrer Institute, Switzerland). It is known that HUVEC do not produce the agonist Angpt1. In addition we know, that inhibition of VE-PTP increases Tie-2 phosphorylation of HUVEC in the absence of added serum or growth factors (Winderlich et al., JCB, 2009). Our flow experiments were performed at low serum concentration (2%) and without any added growth factors. Taken together these reasons suggest that flow-induced activation of Tie-2 is unlikely to require Angpt1 or VEGF. In addition, we have now analyzed flow-induced redistribution and endocytosis of VE-PTP in the presence of the VEGFR2 inhibitor SU-1498 and saw no interference with VE-PTP redistribution (**new expanded view figure 1A; page 8).** Therefore, we assume that angiopoietin 1 and VEGF are not required for VE-PTP redistribution and its consequences.

Referee #2 *(Comments on Novelty/Model System for Author):*

This M/S is providing nice in vivo observations which nevertheless are not corroborated with the few experiments performed on endothelial cells in vitro. There is thus a discrepancy between the two parts of the paper which thus overfall fails to provide a clear mechanism.

Referee #2 *(Remarks for Author):*

TIE2/VE-PTP review

1. *The present study sought to determine the contribution of VE-PTP and Tie2 activation to effects of shear stress on endothelial barrier function and atherogenesis. The approach was to compare VE-PTP redistribution, Tie2 phosphorylation, and macromolecular leakage in regions of mouse aorta known to have contrasting shear stress profiles and in cultured endothelial cells exposed to defined flow conditions. Similar approaches were used to determine the contribution of VE-PTP to leakage and atheroma formation in mice having genetic ablation of VE-PTP in endothelial cells, alone or in combination with apolipoprotein E gene deletion (ApoE-/-) and a high-fat diet.*

2. *Whereas some interesting observations are provided in the second part of the present M/S, it overall fails to provide clear mechanistic insights that could link the first part and the second part of the paper. Indeed, the relationship of downstream polarization of VE-PTP staining in endothelial cells to the blood flow pattern was determined by comparing staining in the aortic arch outer curvature, which has higher average shear, to staining in the inner curvature, where flow is disturbed and average shear is lower. The distribution of VE-PTP staining had dot-like polarization toward the downstream tip of endothelial cells in the outer curvature, but was dispersed in the inner curvature. Shirakura et al. then state that they found that exposure to average high stress led to downstream polarization and endocytosis of*

VE-PTP accompanied by tie2 activation at cell junctions. Figure 1G indeed shows downstream polarization of VE-PTP under flow conditions and figure 2C shows endocytosis of VE-PTP. Yet, this is already well known as demonstrated by Mantilidewi et al. in J. Biol. Chem 2014. Here, internalization is rather weak in response to flow as authors mention 'some tie2 is colocalized with endosome markers'. This should be quantified properly.

To address the request of the reviewer, we have now quantified the colocalization of VE-PTP and the early endosomal marker EEA1 by using ImageJ/Fiji. (We assume the reviewer meant VE-PTP and not Tie2). As shown in **Fig. 2, new part D (page 9)** of the manuscript, co-localization clearly increased significantly over time the longer cells were exposed to shear stress (5, 10 and 30 min).

3. *Then, the authors claim that tie2 is activated at cell junctions but this is not clearly demonstrated (Figure 5C). Instead, the authors use western-blot analyses (phosphor-Tyr) after Tie2 immunoprecipitation in order to show that Tie2 is phosphorylated under flow conditions. This experiment does not demonstrate tie2 activation at cell junctions. Furthermore, heterogenetiy in cell populations makes it difficult to link these findings to the in vivo situation as individual cell behaviors are not necesseraly reflected in population-based measurements.*

To test whether Tie2-pY992 is stimulated at endothelial junctions, we performed immunofluorescence staining for Tie2-pY992 with HUVEC exposed to shear stress or static conditions (**new parts D and E of figure 5**; **page 13).** We clearly observed an increase of the staining signal at junctions upon 5 min exposure to shear stress (left), which was completely blocked upon silencing Tie2 by siRNA (right). Quantification of these signals is shown in **figure 5E**.

In contrast to our *in vivo* staining results, we found *in vitro* background staining under static conditions, which was not due to Tie2-pY992, since Tie2 silencing did not reduce the intensity of these signals. The in vitro staining results prompted us to do additional control experiments to test the specificity of the staining signals *in vivo*. We found that the anti-Tie2-pY992 antibody (the same that we used for staining of HUVEC) showed strong staining of endothelium of the thoracic aorta in WT mice, whereas basically no staining was found in Tie2^{iECKO} mice, where Tie2 was gene inactivated by tamoxifen-inducible PDGF-CreERt (**new supplemental figure 4; page 13**). Background staining of static HUVEC cell cultures is probably partly due to the fact that the *in vivo* signals for Tie2-pY992 are much stronger than the *in vitro* signals in HUVEC. Therefore, background signals were a relatively small contributor in the *in vivo* analysis. In addition, we assume that the expression levels of other cross reactively recognized pY-residues of other proteins may be higher in HUVEC than in mouse aortic endothelium. Nevertheless, our results clearly show that shear stress increases the phosphorylation of Tie2-Y992 at cellular junctions in cultured endothelial cells.

4. *In addition, Mantilidewi et al. demonstrated that redistribution of VE-PTP triggered by shear stress was promoted by integrin engagement of extracellular matrix ligands. In parallel, flow-induced relocation of laminin-associated integrins on the basal endothelial surface is also well appreciated (Béguin et al. Mol Cell Proteomics 2020). Here, the potential link between basal endothelial surface and Tie2 activation is not studied whereas there might be a link as ECM-anchored tie2 promotes assembly of integrin-mediated cell adhesions. Do the authors think that there could be a transition from trans-associated Tie2 to ECM-associated Tie 2. Could this be revealed by performing co-localization experiments with*

vincullin or p-FAK ? Is there any effect on Cytoskeleton remodeling ?

In cultured HUVEC, we did not see obvious effects of flow on Tie2 redistribution to ECM associated sites. However, in aortic endothelium we observed that inhibition of VE-PTP by the pharmacological inhibitor AKB-9785 did not only enhance Tie2 activation at endothelial junctions in the thoracic aorta, it also caused a slight increase of Tie2-pY at focal adhesions marked by anti-paxillin antibodies. (**new supplemental figure 7; page 17**). Thus, VE-PTP can suppresses Tie2 activity *in vivo* at both sites.

Mantilidewi et al. (JBC, 2014) indeed reported on a role of VE-PTP for shear stress-mediated actin remodeling. In agreement with this, we showed recently that VE-PTP stabilizes endothelial junctions via FGD5 driven actin cytoskeleton remodeling (Frye et al. *J Exp Med*., 2015; Braun et al, EMBO Rep. 2019). Thus, we consider VE-PTP and Tie2 to be involved in actin remodeling upon shear stress.

5. *Could the authors observe correlation with Tie2 Y992 and nuclear FOXO-1?*

As requested, we investigated whether Tie2 could play a role in flow-mediated control of FOXO1 activation in shear stress exposed cultured endothelial cells **(new expanded view figure 4, page 11)**. We found that 15 dyn/cm^2 of shear stress reduced nuclear FOXO1 levels within 5 minutes. Tie2 silencing by siRNA attenuated this effect. Thus, shear stress-mediated Tie2 activation is involved in flow-induced FOXO1 inactivation.

6. *What about Tie2 Y820 phosphorylation as VE-PTP inhibition activates FGD5 in two steps: by Tie-2, Rap-1 dependent junction recruitment and by phosphorylation of Y820 (Braun et al. EMBO Rep 2019).*

We agree that it would be interesting to address the phosphorylation of FGD5-Y820. A simple way to do this would be based on staining with an anti FGD5-pY820 antibody, to detect its phosphorylation at junctions upon cell exposure to laminar flow. Unfortunately, we are not aware of any such antibodies. Another approach would be to analyze flow-induced local permeability changes in HUVECs under static and flow conditions and silence endogenous FGD5 by siRNA followed by re-expression of either wt FGD5 or an FGD5-Y820F point mutated version. The problem with such experiments could be that FGD5 would not be knocked down in all cells and the FGD5-Y820F mutant would not be re-expressed in all cells. This could potentially create a mixed cell population of cells having no FGD5, having both forms of FGD5 or only endogenous FGD5 or only the FGD5-Y820F mutant. Depending on the resulting percentages of these different "cell-types", experiments could be difficult to evaluate. Therefore, we prefer to refrain from performing such experiments.

7. *What about the VE-PTP internalization process? Is it ligand dependent or independent?*

This is indeed an interesting question. Previous studies indicated that the extracellular part of VE-PTP plays an essential role for VE-PTP redistribution under shear stress (Mantilidewi et al. J Biol Chem. 2014). However, there are no VE-PTP ligands known with which such experiments could be verified.

8. *It is also well known that other key regulators of endothelial cell permeability such as VE-cadherin and VEGF-R2 are also relevant VE-PTP substrates and it is also well known that VE-cadherin functions as an adaptor that interacts with VEGFRs and promotes VEGFR activation in flow. It might thus well be that the effect of flow on VE-PTP is also mediated via*

these mechanisms.

We tested whether the VEGFR2 inhibitor SU-1498 would interfere with flow-induced VE-PTP redistribution. As shown in the **new expanded view figure 1A (page 8)**, the inhibitor did not interfere with the polarization and endocytosis of VE-PTP.

In addition, Mantilidewi et al. (JBC, 2014) analyzed flow-induced VE-PTP redistribution mainly in VE-PTP transfected non-endothelial HEK293 cells and in endothelial cells which were grown at very sparse confluency, with most cells not being in contact to neighboring cells. In both cases, shear stress-induced VE-PTP redistribution was observed, obviously in the absence of VE-cadherin and PECAM-1in HEK293 cells and largely in the absence of cell contacts in most experiments with endothelial cells. Therefore, it is likely that the mechanosensory complex (PECAM-1/VE-cadherin/VEGF-R2) is not involved in VE-PTP redistribution.

9. *In conclusion, despite nice observations in vivo, mechanistic insights are not clear at all. Authors should focus on in vitro experiments (laminar vs. disturbed/oscillatory and high vs. low shear stress) on HUVEC and better quantify any link between VE-PTP localization/Tie2 activation and endothelial permeability in order to appreciate causality more than correlations. This is not the case in the present paper.*

As response to this comment, we provide the following new results: First, we now show that flow induces Tie2 phosphorylation at endothelial junctions, **(Fig. 5, new parts D and E; page 13)**. Second, as pointed out in our response to reviewer 1, we have performed new experiments where we have analyzed the effects of VE-PTP and Tie2 silencing on shear stress-increased junction integrity of endothelial cells. For this, we established an in vitro assay that allowed us to visualize permeability across HUVEC monolayers under flow. Cells were grown on biotinylated gelatin under flow conditions (15 dyn/cm^2 for 30 min) or static conditions followed by incubation with directly labeled Streptavidin for 3 min. We found that flow clearly reduced paracellular permeability **(new parts E and F of Fig. 6; page 14)**. This effect was lost when either VE-PTP or Tie2 were silenced by siRNA. Thus, VE-PTP and Tie2 are essential for flow-induced junction tightening.

Minor comments:
1. *Conspicuous and conspicuously are used 8 times in the present M/S. Authors should better quantify experimental results appropriately instead.*
We replaced the word at six sites within the text.
2. *Last sentence page 11 is not clear.*
We revised the text accordingly, now at the end of page 12, line 267

Referee #3 *(Comments on Novelty/Model System for Author):*

Interesting but flawed study. The authors failed to consider a major molecular pathway taht would counteract the system they are studying. The anatomic phenotype data are not convincing and raisers a host of questions.

Referee #3 *(Remarks for Author):*

The study by Shirakura et al. reports that differential aorta regions exposed to disturbed flow shear stress have increased baseline vascular leakage which in turn promotes atherosclerotic plaque formation. The authors link this phenotype to cellular redistribution of VE-PTP by

linear vs. disturbed shear that leads to activation of Tie signaling that, in turn, reduces vascular leakage. The authors further demonstrate that VEPTP deletion (or inhibition) increases Tie2 phosphorylation in atheroprone regions and reduces vascular permeability/atheroma formation. Although the study proposes a novel mechanism contributing to atherosclerotic plaque formation, there are a number of important issues that decrease my enthusiasm for the study. Overall, images in the study are beautiful, almost a work of art, but the proposed molecular mechanism are sketchy and the claimed biological effect not all that convincing

1. *The link between Tie2 and VE-TP has been long established and the concept that VE-PTP inhibition activates Tie signaling and contributes to decreased vascular leakage extensively reported including by a number of this manuscript's authors. The novelty here is the purported link to atherosclerosis. In this regard, there are clear differences in VE-PTP distribution in DSS vs. linear shear stress areas. The effect o9n leakage is also quite clear. Yet, the phenotypic effect of endothelial VE-PTP deletion is not that convincing. It seems that the differences in Oil-red-O stating are largely driven by 3-4 outliers in thr VEPTP KO group: two thirds (12 out of 18) of the KO mice who complete overlap in the ORO extent with control group (Fig 7B). The same applies to the extent of leakage (Fig 7D) Atheroma size (Fig 8E shows a similar extent of overlap). It's almost if there are two mice populations after VE-TP KO : those that are much worse and those that are basically unchanged. This get to the molecular mechanisms issues (see below)*

We agree with the reviewer that while it is well established that VE-PTP inhibition leads to activation of Tie2 in endothelial cells, it is indeed novel that the distribution of VE-PTP is differentially affected by different types of shear stress and that we establish a link between VE-PTP redistribution and leak formation in the mouse aorta. The reviewer's assumption that the evidence for an inhibitory effect of VE-PTP gene inactivation on atheroma formation would be mainly based on some outliers in our group of gene inactivated mice prompted us to re-analyze our ORO-stained tissue samples. First of all, even if we ignore the top two values of our aortic arch analysis of ApoE$^{-/-}$/VE-PTP$^{fl/fl}$ mice (Fig. 8B) the results are still highly significant ($p \leq 0.0006$). Second, as outlined also in our response to the next comment (see below), we have now separately analyzed the aortic arch and the brachiocephalic arteries for the KO mice (**new part B of Fig. 8**) and for the AKB9785 inhibitor treated mice (**new part E of Fig. 9**). This analysis clearly shows that the statistically highly significant inhibitory effects of either gene inactivation or treatment with VE-PTP inhibitor are not simply based on statistical outliers.

2. *The analysis of atheromas is very unconvincing. This is not hwo it si normally done ion atherosclerosis literature. One needs to show histological sections from multiple areas (aortic root, brachiocephalic, ascending aorta) where atheromas are clearly visible with proper quantitative assessment of plaque area and inner core size. I should also note that the extent ORO staining is pretty low. I am not sure why. Finally, while the authors show convincing data that VEPTP deletion (or inhibition) completely restores Tie2 phosphorylation in the inner curvature. Yet atheroma size does not appear to be reduced in the inner aorta curvature but only in aortic arc branches (particularly brachiocephalic artery) and not by that much. I think it is important that the authors provide a separate quantification for the inner curvature vs. aortic arch branches for both VEPTP iECKO (fig. 7a) and AKB-9758 experiments (fig 8a). Given this discrepancy, more experimental evidence needs to be provided to support the proposed atheroprotective mechanism. The authors should characterize the aortic branches (e.g. brachiocephalic trunk) and evaluate Tie2*

phosphorylation, permeability, VCAM/ICAM staining in this region in both VEPTP iECKO and AKB-9758 experiments.

In response to the request of the reviewer, we have now performed histological sections of the aortic root of ApoE$^{-/-}$/VE-PTP$^{fl/fl}$ and of ApoE$^{-/-}$/VE-PTP^{iECKO} mice after feeding them for 10 weeks with a high fat diet. As shown for representative images in the **new expanded view figure 5 (page 16)**, the plaque area in the plane of the aortic valve was reduced in VE-PTP gene inactivated mice. We have analyzed 7 to 8 mice in each group. Since it is difficult to guarantee that the section plane is always exactly positioned in the plane of the valve there is more variation in the analysis of such sections from different aortas than if one analyzes whole mounts of the complete aorta. Thus, for technical reasons, this type of analysis is less representative than whole mount staining of the complete aorta with Oil-red-O. It follows that we would need several more mice than for the whole mount analysis, which was so far based on 15 mice per group. Due to limitations in our animal facility, this would require more than at least another 6 months of breeding and animal analysis. Given that an analysis based on histological sections is less representative than the analysis of the complete aorta, we prefer to limit our analysis to this more reliable method.

In addition, we have now re-analyzed our ORO-stained aorta samples, as requested and as outlined above under point 1. We have now separately analyzed the aortic arch and the brachiocephalic arteries for the KO mice and for the AKB9785 inhibitor treated mice as well. This analysis now shows more clearly than before that the inhibitory effects of either gene inactivation or treatment with VE-PTP inhibitor leads to a statistically significant reduction of plaque area in the aortic arch as well as in brachiocephalic arteries (**see new figures 8B and 9E**).

About the reviewer’s comment on low extent of staining with ORO: It needs to be considered that our mice were only fed for 7 to 10 weeks with a high fat diet, which results in smaller atheromas than if mice are fed for longer periods, as is sometimes found in other studies. For the sake of animal protection, we have chosen these shorter time periods.

***3.** Redistribution of VEPTP from dispersed to a polarized intracellular localization nicely correlates with increased Tie2 phosphorylation. I think that the causational link between these two events should be strengthen in vitro by addressing the following points:*
***a.** does exposure to high shear stress induce VEPTP downregulation over time? Let's say at 24hrs after exposure to high shear. This can be easily assessed in vitro by western blot analysis. It would be interesting to know since VEPTP staining in figure 1E seems overall higher in the inner curvature. Higher VEPTP expression in areas exposed to low-shear stress could lead to higher Tie2 dephosphorylation in addition to VEPTP redistribution across all cell surface*

In response to the reviewer’s request, we have analyzed the overall expression levels of VE-PTP in HUVECs under static conditions or upon exposure to 15 dyn/cm^2 of shear stress for 24 hours. As shown in the **new part H of Figure 1 (page 8)**, based on immunoblots of cell lysates, we found that laminar shear stress exposure for 24 hours did not affect the total expression level of VE-PTP. Thus, it is the cell surface expression level and not the overall cellular expression level of VE-PTP which is affected by laminar shear.

b. *Additionally, a confirmation of changes in VEPTP surface level should be performed by surface biotinylation and side-by-side western blot comparison of HUVEC in static conditions vs high shear stress after 30 minutes and 24 hrs.*

We agree with the reviewer that surface biotinylation is a powerful method to analyze protein expression at the cell surface. However, for technical reasons, this assay only gives reliable results if the internalized material is not immediately re-appearing at the cell surface upon stopping shear stress. In order to test, whether internalized VE-PTP does re-appear at the cell surface once flow has stopped, we performed cell surface ELISA assays. Indeed, we found that reduced cell surface levels of VE-PTP after 30 min shear stress recovered quantitatively even as early as 15 min after flow had been stopped (**Fig. 3A, this letter, below**). Therefore, cell surface biotinylation could not be used as a method in this case. Specificity of the VE-PTP cell surface ELISA signal was controlled by 1) isotype control antibody staining, 2) by silencing VE-PTP by siRNA (**Fig. 3B, this letter, below**).

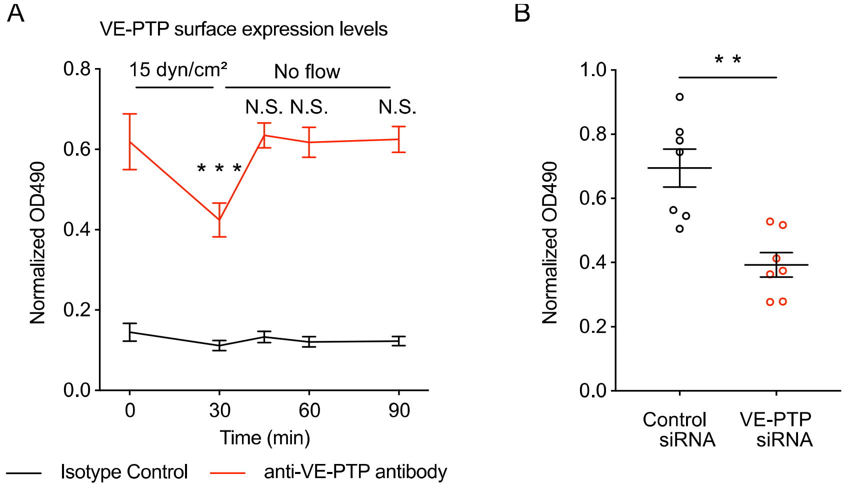

Figure 3 VE-PTP internalization is abrogated after stopping the exposure to shear stress. (A and B) Surface expression levels of VE-PTP in HUVECs detected by cell surface ELISA. **(A)** The cells were exposed to 15 dyn/cm^2 of shear stress for 30 minutes and then cultured without shear stress for 15, 30 and 60 minutes, fixed and analyzed by cell surface ELISA with either isotype control antibodies or anti-VE-PTP antibodies. **(B)** To control the specificity of the VE-PTP signal, cells were first treated with control siRNA or VE-PTP siRNA, followed by fixation 2 days later and cell surface ELISA.

c. *Fig. 1G nicely show that VEPTP become polarized after HUVEC are exposed to high laminar shear stress. Adding Tie2 phosphorylation staining (the pY992 antibody seems very good!) to this time course would strongly argue for a causational link. Since VEPTP redistribution happens quickly, increased pTie2 should be visible already at 5 minutes.*

As suggested, we have stained HUVEC for Tie2-pY992 under static conditions and after 5 min exposure to 15dyn/cm^2 shear stress. As shown in the **new parts D and E of Fig. 5 (page 13)**, we clearly observed an increase of the staining signal for Tie2-pY992 at junctions upon 5 min exposure to shear stress (**new Fig. 5D, left**), which was completely blocked upon silencing Tie2 by siRNA (**new Fig. 5D, right**). Quantification of these signals is shown in **new Figure 5E**. Thus, Tie2 becomes activated at junctions as rapidly as VE-PTP is redistributed and internalized.

In contrast to the *in vivo* staining experiments, we found background staining signals under static conditions which were not due to Tie2-pY992, since Tie2 silencing did not reduce the intensity of these signals. The in vitro staining results prompted us to do additional control experiments to test directly the specificity of the staining signals in vivo. We found that the anti-Tie2-pY992 antibody (the same that we used for staining of HUVEC) showed strong staining of endothelium of the outer aortic arch in WT mice, whereas basically no staining was found in $Tie2^{iECKO}$ mice, where Tie2 was deleted by a tamoxifen-inducible PDGF-CreERt (**new supplemental figure 4**; **page 13).** Background staining of static HUVEC cell cultures is partly due to the fact that the in vivo signals for Tie2-pY992 are much stronger than the in vitro signals in HUVEC, therefore background signals were neglectable in the *in vivo* analysis. In addition, we assume that the expression levels of other cross reactively recognized pY-residues of other proteins may be higher in HUVEC than in mouse aortic endothelium. Nevertheless, our results clearly show that shear stress increases the phosphorylation of Tie2-Y992 at cellular junctions in cultured endothelial cells.

4. *Molecular mechanisms. The authors exclusively focus on VE-PTP KO effect on Tie2 activation. But this VEPTP KO will also increase VEGFR2 activity that will increase permeability. The wto signals may well offset. This is likely why that when it happens (2/3 of the mice) overall effect sin ill and when it does not, you have the phenotype. Thus, VEGFR2 activation, Src activation and VE-cadherin phosphorylation need to be assessed in vitro and in vivo in the presence and absence of VEPTP KO and normal and abnormal shear. The data are not interpretable without this information*

We agree, that VE-PTP inhibition could affect endothelial junction stability in opposite ways via Tie2 and via VEGFR2. Despite these opposite mechanisms, we show here, and have shown before in various inflammation models (Frye et al., J. Exp. Med., 2015), that VE-PTP inhibition leads to junction stabilization *in vivo*. Even VEGF-induced leak formation was suppressed by inhibition of VE-PTP *in vivo* (Frye et al.). Thus, the Tie2 mediated effects are dominant and suppress vascular leakage regardless of potential VEGFR2 activation. Therefore, we feel that potential VEGFR2 activating effects do not affect the interpretation of our current results. We have addressed, though, the question whether VEGFR2 could be involved in shear sensing that leads to VE-PTP redistribution. We found that an inhibitor of VEGFR2 (SU-1498) does not interfere with shear stress-induced VE-PTP redistribution **(new expanded view figure 1A; page 8)**.

a. *VE-PTP inhibitor. The authors make much of the AKB-9785 VE-PTP inhibitor used in this study and suggest it could be a useful therapeutic agent. That seems highly doubtful. The drugs seems to have failed in TIME-2B study the authors mention that has never been published. I infer that from the fact that the company behind the drug no longer exists. I am rather skeptical that a systemic inhibition of VEPTP can be a good thing especially when carried out on a chronic basis as would be required for atherosclerosis therapy.*

There are many examples of drug classes initially developed for one indication, that were subsequently more successful in a different indication based on the same mechanism. In fact, in TIME2b there was a small, but statistically insignificant, benefit in reduction of progression of diabetic retinopathy and no significant safety issues after almost a year of daily subcutaneous treatment in this high risk diabetic population

(https://www.businesswire.com/news/home/20190318005228/en/Aerpio-Pharmaceuticals-Announces-Results-From-TIME-2b-Study-of-AKB-9778-in-Diabetic-Retinopathy). This is in agreement with the viability and healthy status of VE-PTP^{iECKO} mice in our lab. Although the TIME2b data were not formally published, data for the TIME2 study was published (Campochiaro et al. 2016). In this phase 2 study in patients with diabetic macular edema (DME), addition of Tie2 activation via VE-PTP inhibition with AKB-9778 to standard of care anti-VEGF treatment resulted in a statistically significant reduction of macular edema compared to anti-VEGF therapy alone without significant safety issues over the three months of treatment. Importantly, TIME2 was the first randomized, placebo-controlled study to demonstrate a statistically significant benefit of any therapy in combination with anti-VEGF therapy in patients with DME. In addition to reduced vascular leakage, Tie2 activation via VE-PTP inhibition could have other beneficial effects in the diseased vasculature including improved endothelial function via activation of eNOS downstream of Tie2 (Siragusa et al. 2021). Other preclinical studies have demonstrated both anti-inflammatory and anti-thrombotic actions of Tie2 activation via VE-PTP inhibition (papers cited in our manuscript: Carota et al., Goel et al., Li et al., Shen et al., and Higgins et al., JCI, 2018, 128: 1471-1484), both of which could be beneficial in patients with atherosclerosis. Thus, based on these published clinical and preclinical data along with the current data, Tie2 activation via VE-PTP inhibition could provide significant benefits for patients with atherosclerotic cardiovascular disease.

5. *Other comments:*

a. *In Figure 1-B, VE-PTP seems highly expressed in some of the cells of the inner curvature and not in others, while in the text the authors described an overall lower level of VE-PTP expression in the inner side compared to the outer side. How would you explain this? Can you provide a picture at lower magnification?*

We are afraid there might be some misunderstanding. Fig. 1B does not show VE-PTP staining in the inner curvature, instead this figure depicts the thoracic aorta. Nevertheless, we agree with the reviewer that there is some variation in the staining pattern for VE-PTP, which is also highlighted in Fig. 1C (again depicting thoracic aorta). We assume that this variance may reflect the complicated flow pattern due to different positions of endothelial cells with respect to branching of intercoastal arteries and possibly other spatial differences. In general, we do find clear differences of VE-PTP localization between the inner and outer curvature at high as well as at low magnification (Original Figure 1E and Expanded View Figure 3 (the latter is identical to old supplemental Figure 4)). In contrast to the subcellular distribution of VE-PTP, shear stress did not alter the total cellular expression level of VE-PTP, as we found in HUVEC **(new part H of Fig. 1, page 8)**.

b. *In Figure 2-C and D the authors show by immunofluorescence the decrease of VE-PTP expression at the cell membrane and the increase of internalized VE-PTP. It would be good to confirm these data by showing biochemically the decrease of surface VE-PTP versus whole cell VE-PTP expression with increasing exposure time to laminar shear flow.*

As we mentioned in our responses to Reviewer 3 (comment 3b), we were unable to determine cell surface expression levels of VE-PTP by biochemical means such as cell surface biotinylation, since as soon as flow is stopped, VE-PTP becomes re-expressed on the cell

surface within 15 min **(see figure 3, this letter, above)**. We do, though, clearly detect changes in the cell surface expression by cell surface ELISA assays. In addition, we have shown, that shear stress does not alter the total cellular expression level of VE-PTP **(new part H of Fig. 1, page 8)**.

c. *The authors suggest that Tie2 activation depends on VE-PTP internalization. To corroborate this the authors should test the expression of Tie2-pY992 and VE-PTP after inhibition of endocytosis in HUVEC under laminar flow.*

We have tested whether classical endocytosis inhibitors such as Dynasore and Pitstop-2 suppress the internalization of VE-PTP **(new expanded view figure 2; page 10)**. Unexpectedly, neither Dynasore nor Pitstop-2 treatment resulted in a clear inhibitory effect of VE-PTP internalization while they did inhibit endocytosis of Transferrin labeled with Alexa488. These results indicate that VE-PTP internalization is independent of Dynamin and Clathrin. Therefore, we assume that VE-PTP is endocytosed by non-classical mechanisms (reviewed in references Maldonadi-Báez et al., 2013; and Mayor et al. 2014), which will need to be analyzed in the future.

d. *In aortas VE-PTP is expressed at the downstream pole of ECs, however HUVEC cells exposed to laminar flow for 24 hours, do not express this strong VE-PTP polarization (supplemental figure2 and figure 1 G) how do you explain this difference?*

One possible explanation is that only *in vivo* endothelial cells were exposed to pulsatile laminar flow whereas in vitro the cells were exposed to sustained laminar flow. To test whether this would explain the different results, we exposed HUVEC to 15 dyn/cm^2 of laminar shear stress with or without a pulse of 1 Hz. To this end we used flow chamber that had been established by the Schnittler lab (University of Münster) (Buschmann et al., Biotechnol. Bioeng. 2005, 89:493-502) which is based on a cone-and-plate apparatus. As shown in **Figure 4 of this letter (see below)**, we did not observe that pulsatile flow had different effects than non-pulsatile flow. Thus, pulsatility at a frequence of 1 Hz (1 pulse per second) was not able to copy the *in vivo* situation in our *in vitro* experiments.

Mice hearts beat at a rate of 400 to 500 beats/min. For technical reasons, it was not possible to test such high frequencies in our in vitro flow chambers. Thus, we cannot rule out that pulsatility is the reason for the differences between our *in vitro* and *in vivo* results. Alternatively, many other reasons are conceivable that could explain differences between aortic endothelial cells and in vitro cultured HUVEC. We addressed this in the discussion on page 20, lines 438-441.

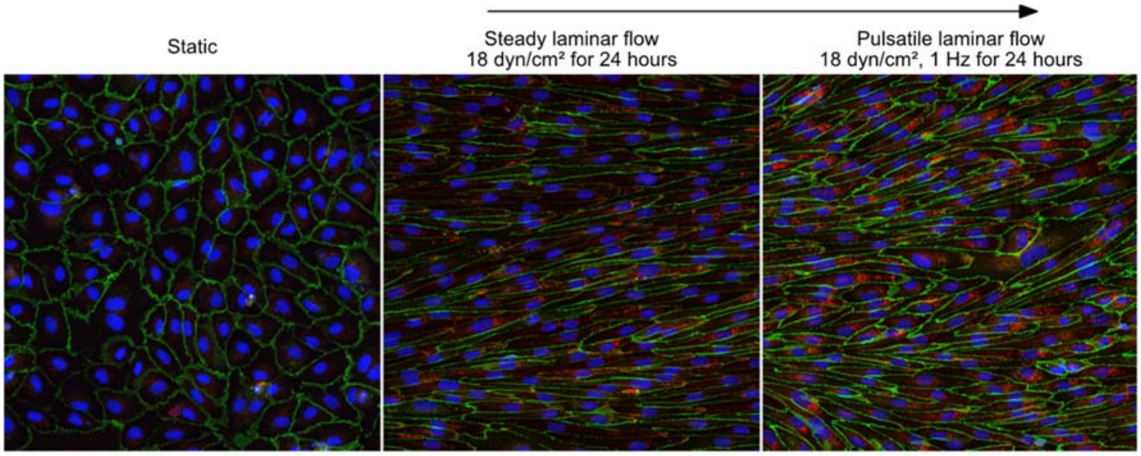

Figure 4 Analysis of the effect of pulsatility of laminar shear stress on VE-PTP subcellular distribution. HUVECs were exposed to steady laminar flow (18 dyn/cm^2) and pulsatile laminar flow (18 dyn/cm^2, 1 Hz) for 24 hours (as indicated). Subsequently, cells were fixed and stained for VE-PTP (red) and VE-cadherin (green).

e. *Authors show a correlation between Tie2-pY992 expression and VE-PTP internalization. What is tie2-pY992 expression in HUVECs under static flow, when VE-PTP is less expressed?*

We have previously reported that silencing of VE-PTP by siRNA in HUVEC strongly increases Tie2-pY992 levels in the absence of flow (Winderlich et al. *J Cell Biol.* 2009 185: 657–671). Thus, less VE-PTP expression leads to the phosphorylation of Tie2 Y992. VE-PTP is indeed a major regulator of Tie2 activity.

Referee #4 *(Comments on Novelty/Model System for Author):*

The medical impact refers to the delivery regime used in the study. The medical impact would be higher with a more realistic dosing.

Referee #4 *(Remarks for Author):*

This study by Shirakura and colleagues, investigates the consequence of VE-PTP loss on the development of atherosclerosis in an animal model. Depletion is either genetically or with the VE-PTP inhibitor, AKB9785. They further investigate the mechanism of redistribution and loss in endothelial cells in athero-prone regions in the aorta and in in vitro models.
The study is well performed and the in vivo images especially are very impressive. However, I have a number of issues that require addressing

1. *The authors need to address the mechanism underlying the inhibition of atheroma development. They show leak is inhibited. They show atheroma is inhibited by the drug or genetically, but no change in the lipid profiles. But what is happening here?*
Is there less lipid accumulation in the tissue? Or inflammatory infiltrate, even though they

report no change in VCAM and ICAM on the endothelium? More detailed investigations into the progression of atherosclerosis is needed.

It is well established that different types of flow patterns at different sites in the aorta determine the atheroprone and atheroprotective fate of these sites with high laminar flow being protective and low turbulent flow being supportive for atheroma formation. Two main reasons are known why flow determines atheroma formation. High laminar flow is known to, first, enhance the barrier function of endothelium and, second, to prevent an inflammation type transcription profile, while low turbulent flow supports leak formation and an inflammation related transcriptional response.

As the reviewer correctly points out, we found that inhibition of VE-PTP protects against endothelial leaks and against atheroma formation. On the other hand, we found that the expression of typical inflammatory antigens, such as ICAM-1 and VCAM-1 was not prevented in atheroprone areas by inhibiting VE-PTP. Thus, VE-PTP is unlikely to be relevant for the transcriptional inflammatory response of the endothelium, but is an essential player for flow-dependent protection against leak formation. In addition, we could exclude that lipid profiles in the circulation were affected by the expression of VE-PTP. Furthermore, we show that VE-PTP is segregated away from Tie2 by high laminar flow which leads to Tie2 activation, which prevents leaks at atheroprotected sites. Collectively, our results suggest that VE-PTP inhibition protects against atheroma formation due to its protective effect against leaks. We believe, that this effect reduces the chance for lipoprotein particles to enter the arterial wall. Indeed, we found that Oil-red-O staining was reduced which stains triglycerides and cholesterol.

Despite all this, we agree with the reviewer that we cannot exclude that, in addition, leukocyte entry into the aortic wall may also be affected, since VE-PTP inhibition can reduce leukocyte extravasation in inflammation models via activation of Tie2 (Frye et al., J. Exp. Med., 2015). Therefore, we have now stated this **in the discussion on page 24, lines 531-533** Nevertheless, both these effects would be based on VE-PTP dependent endothelial barrier enhancement via Tie2.

2. *Therapeutically, delivery of AKB-9785 every day for 11 weeks is a mammoth delivery regime and one that is unlikely to be accepted. The authors need to present more realistic treatment options, even for proof-of-principle studies. A change in delivery regime may also be essential to determine the mode of action to inhibit atheroma development (Point 1 above). When can you intervene in the process and still get effects?*

In the clinical study TIME-2b, subcutaneous administration of AKB-9778 15mg once or twice daily for 48 weeks was given to diabetic retinopathy patients. Thus, this administration schedule was approved for a clinical study.

Continued clinical development of these small molecule VE-PTP inhibitors could include reformulation to a long-acting subcutaneous depot. Alternatively, therapeutic antibodies that bind the VE-PTP extracellular domain could be developed for once monthly delivery. Developing an orally bioavailable VE-PTP inhibitor may be ideal for chronic dosing but is a challenge for the future. The goal of this study was to explore the potential of Tie2 activation via VE-PTP inhibition in a preclinical model of atherosclerosis.

3. *Mechanistically, the authors need to address what controls the redistribution of VE-PTP. Schwartz and colleagues have shown that Rho is regulated and is important in flow mediated alignment. Is VE-PTP redistribution also Rho regulated?*

In response to the reviewer's request, we have now analyzed a potential role of RhoA for the mechanism that triggers VE-PTP redistribution. To this end, we have silenced RhoA in HUVEC by siRNA, followed 48 hrs later by exposure of the cells to 15 dyn/cm^2 shear stress for 5, 10 and 30 min and stained subsequently for VE-PTP. As shown in **(new expanded view figure 1B; page 8)**, silencing of RhoA did not interfere with shear stress-induced redistribution of VE-PTP. This is in line with results from Mantilidewi et al (JBC, 2014, 289:6451-6461) who showed that inhibition of ROCK did not interfere with VE-PTP redistribution. We conclude that the redistribution of VE-PTP is independent of RhoA.

***4.** The endothelial cells align even in the absence of VE-PTP (Fig5A). Alignment is considered a protective mechanism of EC in the atheroprotective sites. Is there a link between alignment and loss of VE-PTP? If polarization of VE-PTP is inhibited, is alignment blocked? If alignment is blocked does VE-PTP redistribute, etc?*

Matozaki's group demonstrated that VE-PTP silencing impaired flow-mediated cell elongation under shear stress, although these experiments were performed under rather sparse culture conditions (Mantilidewi et al. *J Biol Chem*. 2014, 289:6451-6461). In line with these results, we observed that VE-PTP siRNA slightly suppresses cell alignment in cultured endothelial cells (Supplemental Figure 1G and H).

In vivo, induced gene inactivation of VE-PTP in adult mice did not affect endothelial cell morphology in the thoracic aorta (Supplemental Figure 1D and E) and did not affect cell alignment in the outer curvature (figure 5A). However, this does not necessarily rule out a possible role for VE-PTP in cell alignment *in vivo*, since it is likely that cell morphology and alignment of endothelial cells in the aorta were already determined at birth before VE-PTP gene inactivation was induced. Gene inactivation of VE-PTP in the embryo causes vascular malformations and is lethal, whereas gene inactivation in adults does not impair viability.

Since we do not know a specific way how to block shear stress-induced VE-PTP redistribution, we cannot test whether this redistribution is necessary for cell alignment.

The second question, whether cell alignment is required for VE-PTP redistribution can be clearly answered, since shear stress-induced cell alignment is a process that requires several hours whereas shear stress-induced VE-PTP redistribution already occurs within 5 min after exposure to flow. Thus, cell alignment is not required for VE-PTP redistribution.

***5.** The authors talk about the selectivity of AKB-9778 for VE-PTP. Some comment on how 9778 and 9785 differ and the selectivity of AKB-9785 is needed.*

These two inhibitors are very closely related chemical congeners with similar sub-nanomolar potency for VE-PTP and similar high degree of selectivity against other phosphatases. This is now staed in M & M on page 26. It is common to use such close chemical congeners once the lead compound, in this case AKB-9778, reaches clinical development. AKB-9785 was used in multiple published preclinical studies, such as Shen et al., 2014; Braun et al., 2019.

***6.** The localization of VE-PTP to endosomes needs strengthening rather than only colocalization by single images. Some biochemical studies to give support and also perhaps inhibitor studies should be shown.*

We have now quantified the colocalization of VE-PTP and the early endosomal antigen EEA1. As shown in the **new part D of figure 2 (page 9)** of the manuscript, co-localization clearly increased significantly over time (5, 10 and 30 min after onset of flow).

We agree that it would be desirable to have some biochemical evidence such as cell surface biotinylation to verify internalization of VE-PTP. However, for technical reasons, this assay only gives reliable results if the internalized material is not immediately re-appearing at the cell surface upon stopping shear stress. In order to test, whether internalized VE-PTP does re-appear at the cell surface once flow has stopped, we performed cell surface ELISA assays. Indeed, we found that reduced cell surface levels of VE-PTP after 30 min shear stress recovered quantitatively even as early as 15 min after flow had been stopped **(Fig. 3A, this letter, see above)**. Therefore, cell surface biotinylation could not be used as a reliable method in this case. Specificity of the VE-PTP cell surface ELISA signal was controlled by 1) isotype control antibody staining, 2) by silencing VE-PTP by siRNA **(Fig. 3B, this letter, see above)**.

We tested also whether classical endocytosis inhibitors such as Dynasore and Pitstop-2 would block shear stress-induced VE-PTP uptake into cells. As pointed out in our comments for reviewer 3, (point 5c), neither Dynasore nor Pitstop-2 treatment resulted in a strong inhibitory effect of VE-PTP internalization, while they did inhibit endocytosis of Transferrin labeled with Alexa488. These results are now reported on page 10, lines 208-211 and shown in the **new expanded view figure 2 (page 10)**. These results indicate that VE-PTP internalization is independent of dynamin and clathrin. Therefore, we assume that VE-PTP is endocytosed by non-classical mechanisms (reviewed in references Maldonadi-Báez et al., 2013; and Mayor et al. 2014), which will need to be analyzed in the future. These results are now mentioned in the **discussion (page 20)**.

7. *In the aorta, there are regions where the cells are aligned, VE-PTP is polarized and cells in the next region where there are aligned, and VE-PTP mostly is lost (eg Fig3A & B). This change in the level of expression of VE-PTP, does this equate to differences in the activation status of the cell? Perhaps using p65 of the NFkB complex may help?*

We agree with the reviewer’s observation. We attempted to stain for NFκB, but due to specificity and sensitivity problems were not able to clarify the reason for endothelial heterogeneity. It is well known that antigens such as ICAM-1 and VCAM-1 show heterogenous expression, under baseline as well as under inflammatory conditions (see for example figure 7) and we know that this occurs even *in vitro* in cultured HUVECs. The basis for this is unclear, as well as whether this is indeed physiologically relevant.

8. *Fig2C-localisation to the plasma membrane is not easy to see. A specific marker needs to be included.*

Fig. 2C is shown in order to document that VE-PTP becomes endocytosed and is found in vesicles that partially co-localize with the early endosomal marker EEA1. We have now improved the analysis by quantifying the colocalization of VE-PTP and EEA1, which we show in the **new panel D of Fig. 2 (page 9)**. These results show that co-localization clearly increases over time. The longer cells are exposed to shear stress, the more EEA1 positive vesicles become positive for VE-PTP. We agree with the reviewer that our statement: “large patches of VE-PTP at the downstream tip of HUVEC at 5 or 10 min that lacked EEA1 staining appeared to be in the plasma membrane (Figure 2C)” is not directly backed by the evidence in Fig. 2C. Evidence that the VE-PTP patches at the downstream pole of the cells represent VE-PTP on the plasma membrane is actually based on Fig. 2D. We have now revised our statements about Fig. 2C accordingly **(page 9, lines 191-194)**.

9. *Fig3E. The intensity of VE-Cadherin is so much stronger in the inner curvature and this doesn't fit with the loss of Tie2-pY992 and likely a weaker junction.*

We agree that staining for VE-cadherin results in stronger signals in the inner curvature than in the outer curvature. This is in agreement with results by others (Heo, KS et al. *Mol Cells.* 014 Jun;37(6):435-40). It was also recently reported that low oscillatory shear stress increases VE-cadherin transcription in contrast to high laminar shear stress (Mahmoud, MM et al. Sci Rep. 2017 Jun 13;7(1):3375). This may indicate that athero-prone flow might upregulate VE-cadherin expression in the inner curvature, maybe as an attempt to compensate the existing loss of endothelial junction integrity. Nevertheless, despite this attempt, the endothelium in the inner curvature is more leaky than in the outer curvature. This highlights the fact that VE-cadherin is only one component of endothelial junction integrity. As we have shown before, Tie2-mediated barrier stabilization can even be demonstrated *in vivo* in the absence of VE-cadherin (Frye et al., J. Exp. Med. 2015). Likewise, we just demonstrated that VE-cadherin expression is not essential for shear stress-mediated barrier enhancement (Figure 2 in this letter, see above). Taken together, the VE-PTP/Tie2 system can control endothelial junction integrity in a way that is only partially influenced by VE-cadherin.

10. *Supplementary Fig8A in lung is not discussed.*

We have mentioned this supplemental figure on page 17, lines 371-372.

11. *Is the anti-fibrinogen measurement of leak always paracellular?*

As we show in supplemental figure 3E (old Suppl Fig. 5E), 20 nm microspheres leaked in the endothelium of the inner curvature mainly at tricellular junctions, thus passed the endothelium on a paracellular route. Since the size of IgG is with approximately 10 nm in the same range as the microspheres, we assume that anti fibrinogen antibodies leaked through the same route.

7th Dec 2022

Dear Prof. Vestweber,

Thank you for the submission of your revised study to EMBO Molecular Medicine. We have now received feedback from the three referees who re-reviewed your manuscript (and had reviewed your initial manuscript). These referees also evaluated your answers to referee #2's concerns. As you will see below, they are now supportive of publication, and I am therefore pleased to inform you that we will be able to accept your manuscript once the following editorial points will be addressed:

1/ Manuscript text:
- Please address the queries from the data editors in the data edited file. This file will be sent to you in the next couple of days. Please use this file for further modifications, remove the blue text and only keep in track changes mode any new changes.
- We can accept a maximum of 5 keywords, please adjust accordingly.
- It is mandatory to include a 'Data Availability' section after the Materials and Methods. Primary datasets produced in this study need to be deposited in an appropriate public database, and the accession numbers and database listed under 'Data Availability'. In case you have no data that requires deposition in a public database, please state so in this section (This study includes no data deposited in external repositories). Note that the Data Availability Section is restricted to new primary data that are part of this study.
- Author contributions: CRediT has replaced the traditional author contributions section because it offers a systematic machine readable author contributions format that allows for more effective research assessment. Please remove the Authors Contributions from the manuscript and use the free text boxes beneath each contributing author's name in our system to add specific details on the author's contribution. More information is available in our guide to authors.
- Please rename the "Conflict of interest" section: "Disclosure statement and competing interests": We updated our journal's competing interests policy in January 2022 and request authors to consider both actual and perceived competing interests. Please review the policy https://www.embopress.org/competing-interests and update your competing interests if necessary.

2/ Figures.
- Please indicate for all figures or in their legends the exact p= values, not a range. Some people found that to keep the figures clear, providing a supplemental table in an Appendix with all exact p-values was preferable. You are welcome to do this if you want to.
- Please upload your figures as individual .eps, .tif, .jpg (one file per figure). For guidance, download the 'Figure Guide PDF' (https://www.embopress.org/page/journal/17574684/authorguide#figureformat).
- You currently have 10 figures. We usually accommodate up to 8 figures. We suggest that you make some figures/figure panels Expanded View figures: we replaced Supplementary Information with Expanded View (EV) Figures and Tables that are collapsible/expandable online. A maximum of 5 EV Figures can be typeset. EV Figures should be cited as 'Figure EV1, Figure EV2" etc... in the text and their respective legends should be included in the main text after the legends of regular figures.
- For the figures that you do NOT wish to display as Expanded View figures, they should be bundled together with their legends in a single PDF file called *Appendix*, which should start with a short Table of Content. Appendix figures should be referred to in the main text as: "Appendix Figure S1, Appendix Figure S2" etc. Appendix figure legends should part of the appendix file and removed from the main manuscript text.

3/ At EMBO Press we encourage authors to provide source data for the main figures. Numerical data should be provided as individual .xls or .csv files (including a tab describing the data). For blots or microscopy, uncropped images should be submitted (using a zip archive if multiple images need to be supplied for one panel). Additional information on source data and instruction on how to label the files are available at
.

4/ Please provide The paper explained: EMBO Molecular Medicine articles are accompanied by a summary of the articles to emphasize the major findings in the paper and their medical implications for the non-specialist reader. Please provide a draft summary of your article highlighting
- the medical issue you are addressing,
- the results obtained and
- their clinical impact.
This may be edited to ensure that readers understand the significance and context of the research. Please refer to any of our published articles for an example.

5/ Every published paper now includes a 'Synopsis' to further enhance discoverability. Synopses are displayed on the journal webpage and are freely accessible to all readers. They include a short stand first (maximum of 300 characters, including space) as well as 2-5 one-sentences bullet points that summarizes the paper. Please write the bullet points to summarize the key NEW findings. They should be designed to be complementary to the abstract - i.e. not repeat the same text. We encourage inclusion of key acronyms and quantitative information (maximum of 30 words / bullet point). Please use the passive voice. Please upload

them as a separate file.

Please also suggest a striking image or visual abstract to illustrate your article as a PNG file 550 px wide x 300-600 px high.

6/ As part of the EMBO Publications transparent editorial process initiative (see our Editorial at http://embomolmed.embopress.org/content/2/9/329), EMBO Molecular Medicine will publish online a Review Process File (RPF) to accompany accepted manuscripts.
This file will be published in conjunction with your paper and will include the anonymous referee reports, your point-by-point response and all pertinent correspondence relating to the manuscript. Let us know whether you agree with the publication of the RPF and as here, if you want to remove or not any figures from it prior to publication.
Please note that the Authors checklist will be published at the end of the RPF.

I look forward to receiving your revised manuscript.

With kind regards,

Lise Roth

Lise Roth, PhD
Senior Editor
EMBO Molecular Medicine

***** Reviewer's comments *****

Referee #1 (Remarks for Author):

The authors had made substantial additional work to delineate the underlying mechanisms. The new data are robust and well executed.
Overall, they have found interesting pathways regulating permeability under dynamic flow that indeed involve known mediators (VE-PTP, Tie2, VE-cad) but with somehow underestimated/unexpected modus operandi.

Referee #3 (Remarks for Author):

Very much appreciate detailed replies and lots of extra work
No further questions

Referee #4 (Remarks for Author):

The authors have addressed the concerns raised in my review.
The study will add to our understanding of vascular leak, and its complex mechanisms of control and its impact in atherosclerosis.It is a very detailed and interesting study.

The authors addressed the minor editorial issues.

11th Jan 2023

Dear Prof. Vestweber,

Thank you for sending your revised file. I am pleased to inform you that your manuscript is now accepted for publication and will be sent to our publisher to be included in the next available issue of EMBO Molecular Medicine!

Please read below for additional IMPORTANT information regarding your article, its publication and the production process.

Congratulations on your interesting work,

With kind regards,

Lise Roth

Lise Roth, Ph.D
Senior Editor
EMBO Molecular Medicine

Follow us on Twitter @EmboMolMed
Sign up for eTOCs at embopress.org/alertsfeeds

*** *** *** IMPORTANT INFORMATION *** *** ***

SPEED OF PUBLICATION
The journal aims for rapid publication of papers, using using the advance online publication "Early View" to expedite the process: A properly copy-edited and formatted version will be published as "Early View" after the proofs have been corrected. Please help the Editors and publisher avoid delays by providing e-mail address(es), telephone and fax numbers at which author(s) can be contacted.

Should you be planning a Press Release on your article, please get in contact with embomolmed@wiley.com as early as possible, in order to coordinate publication and release dates.

LICENSE AND PAYMENT:

All articles published in EMBO Molecular Medicine are fully open access: immediately and freely available to read, download and share.

EMBO Molecular Medicine charges an article processing charge (APC) to cover the publication costs. You, as the corresponding author for this manuscript, should have already received a quote with the article processing fee separately. Please let us know in case this quote has not been received.

Once your article is at Wiley for editorial production you will receive an email from Wiley's Author Services system, which will ask you to log in and will present you with the publication license form for completion. Within the same system the publication fee can be paid by credit card, an invoice, pro forma invoice or purchase order can be requested.

Payment of the publication charge and the signed Open Access Agreement form must be received before the article can be published online.

PROOFS

You will receive the proofs by e-mail approximately 2 weeks after all relevant files have been sent o our Production Office. Please return them within 48 hours and if there should be any problems, please contact the production office at embopressproduction@wiley.com.

Please inform us if there is likely to be any difficulty in reaching you at the above address at that time. Failure to meet our deadlines may result in a delay of publication.

All further communications concerning your paper proofs should quote reference number EMM-2022-16128-V3 and be directed to the production office at embopressproduction@wiley.com.

EMBO Press Author Checklist

Corresponding Author Name: Luca Tamagnone - Enrico Giraudo	
Journal Submitted to: EMBO Molecular Medicine	
Manuscript Number: EMM-2022-16104	

USEFUL LINKS FOR COMPLETING THIS FORM
The EMBO Journal - Author Guidelines
EMBO Reports - Author Guidelines
Molecular Systems Biology - Author Guidelines
EMBO Molecular Medicine - Author Guidelines

Reporting Checklist for Life Science Articles (updated January 2022)
This checklist is adapted from Materials Design Analysis Reporting (MDAR) Checklist for Authors. MDAR establishes a minimum set of requirements in transparent reporting in the life sciences (see Statement of Task: 10.31222/osf.io/9sm4x). Please follow the journal's guidelines in preparing your manuscript.
Please note that a copy of this checklist will be published alongside your article.

Abridged guidelines for figures
1. Data
The data shown in figures should satisfy the following conditions:
- the data were obtained and processed according to the field's best practice and are presented to reflect the results of the experiments in an accurate and unbiased manner.
- ideally, figure panels should include only measurements that are directly comparable to each other and obtained with the same assay.
- plots include clearly labeled error bars for independent experiments and sample sizes. Unless justified, error bars should not be shown for technical replicates.
- if n<5, the individual data points from each experiment should be plotted. Any statistical test employed should be justified.
- Source Data should be included to report the data underlying figures according to the guidelines set out in the authorship guidelines on Data Presentation.

2. Captions
Each figure caption should contain the following information, for each panel where they are relevant:
- a specification of the experimental system investigated (eg cell line, species name).
- the assay(s) and method(s) used to carry out the reported observations and measurements.
- an explicit mention of the biological and chemical entity(ies) that are being measured.
- an explicit mention of the biological and chemical entity(ies) that are altered/varied/perturbed in a controlled manner.
- the exact sample size (n) for each experimental group/condition, given as a number, not a range;
- a description of the sample collection allowing the reader to understand whether the samples represent technical or biological replicates (including how many animals, litters, cultures, etc.).
- a statement of how many times the experiment shown was independently replicated in the laboratory.
- definitions of statistical methods and measures:
  - common tests, such as t-test (please specify whether paired vs. unpaired), simple χ2 tests, Wilcoxon and Mann-Whitney tests, can be unambiguously identified by name only, but more complex techniques should be described in the methods section;
  - are tests one-sided or two-sided?
  - are there adjustments for multiple comparisons?
  - exact statistical test results, e.g., P values = x but not P values < x;
  - definition of 'center values' as median or average;
  - definition of error bars as s.d. or s.e.m.

Please complete ALL of the questions below.
Select "Not Applicable" only when the requested information is not relevant for your study.

Materials

Newly Created Materials	Information included in the manuscript?	In which section is the information available? (Reagents and Tools Table, Materials and Methods, Figures, Data Availability Section)
New materials and reagents need to be available; do any restrictions apply?	Not Applicable	

Antibodies	Information included in the manuscript?	In which section is the information available? (Reagents and Tools Table, Materials and Methods, Figures, Data Availability Section)
For **antibodies** provide the following information:<br>- Commercial antibodies: RRID (if possible) or supplier name, catalogue number and or/clone number<br>- Non-commercial: RRID or citation	Yes	Materials and Methods

DNA and RNA sequences	Information included in the manuscript?	In which section is the information available? (Reagents and Tools Table, Materials and Methods, Figures, Data Availability Section)
**Short novel DNA or RNA including primers, probes**: provide the sequences.	Yes	Materials and Methods

Cell materials	Information included in the manuscript?	In which section is the information available? (Reagents and Tools Table, Materials and Methods, Figures, Data Availability Section)
**Cell lines:** Provide species information, strain. Provide accession number in repository **OR** supplier name, catalog number, clone number, and/**OR** RRID.	Yes	Materials and Methods
**Primary cultures:** Provide species, strain, sex of origin, genetic modification status.	Yes	Materials and Methods (details in referenced prior publication)
Report if the cell lines were recently **authenticated** (e.g., by STR profiling) and tested for mycoplasma contamination.	Yes	Materials and Methods

Experimental animals	Information included in the manuscript?	In which section is the information available? (Reagents and Tools Table, Materials and Methods, Figures, Data Availability Section)
**Laboratory animals or Model organisms:** Provide species, strain, sex, age, genetic modification status. Provide accession number in repository **OR** supplier name, catalog number, clone number, **OR** RRID.	Yes	Materials and Methods
**Animal observed in or captured from the field:** Provide species, sex, and age where possible.	Not Applicable	
Please detail **housing and husbandry conditions**.	Yes	Materials and Methods

Plants and microbes	Information included in the manuscript?	In which section is the information available? (Reagents and Tools Table, Materials and Methods, Figures, Data Availability Section)
**Plants:** provide species and strain, ecotype and cultivar where relevant, unique accession number if available, and source (including location for collected wild specimens).	Not Applicable	
**Microbes:** provide species and strain, unique accession number if available, and source.	Not Applicable	

Human research participants	Information included in the manuscript?	In which section is the information available? (Reagents and Tools Table, Materials and Methods, Figures, Data Availability Section)
If collected and within the bounds of privacy constraints report on age, sex and gender or ethnicity for all study participants.	Yes	Table EV1

Core facilities	Information included in the manuscript?	In which section is the information available? (Reagents and Tools Table, Materials and Methods, Figures, Data Availability Section)
If your work benefited from core facilities, was their service mentioned in the acknowledgments section?	Not Applicable	

Design

Study protocol	Information included in the manuscript?	In which section is the information available? (Reagents and Tools Table, Materials and Methods, Figures, Data Availability Section)
If study protocol has been **pre-registered, provide DOI in the manuscript**. For clinical trials, provide the trial registration number **OR** cite DOI.	Not Applicable	
Report the **clinical trial registration number** (at ClinicalTrials.gov or equivalent), where applicable.	Not Applicable	

Laboratory protocol	Information included in the manuscript?	In which section is the information available? (Reagents and Tools Table, Materials and Methods, Figures, Data Availability Section)
Provide DOI OR other citation details if **external detailed step-by-step protocols** are available.	Not Applicable	

Experimental study design and statistics	Information included in the manuscript?	In which section is the information available? (Reagents and Tools Table, Materials and Methods, Figures, Data Availability Section)
Include a statement about **sample size** estimate even if no statistical methods were used.	Yes	Sample size was estimated according to experimental experience and prior scientific literature, taking in account sample/parameter intrinsic variablity.
Were any steps taken to minimize the effects of subjective bias when allocating animals/samples to treatment (e.g. **randomization procedure**)? If yes, have they been described?	Yes	No randomization is required (or applicable) for the described experiments
Include a statement about **blinding** even if no blinding was done.	Yes	In general, blinding was not applied, but data analysis was validated by at least two scientists.
Describe **inclusion/exclusion criteria** if samples or animals were excluded from the analysis. Were the criteria pre-established?<br><br>If sample or data points were omitted from analysis, report if this was due to attrition or intentional exclusion and provide justification.	Yes	Results of experiments in which appropriate negative/positive controls were not validated were excluded from the analysis.
For every figure, are **statistical tests** justified as appropriate? Do the data meet the assumptions of the tests (e.g., normal distribution)? Describe any methods used to assess it. Is there an estimate of variation within each group of data? Is the variance similar between the groups that are being statistically compared?	Yes	Figure legends

Sample definition and in-laboratory replication	Information included in the manuscript?	In which section is the information available? (Reagents and Tools Table, Materials and Methods, Figures, Data Availability Section)
In the figure legends: state number of times the experiment was **replicated** in laboratory.	Yes	Figure Legends
In the figure legends: define whether data describe **technical or biological replicates**.	Yes	Figure Legends

Ethics

Ethics	Information included in the manuscript?	In which section is the information available? (Reagents and Tools Table, Materials and Methods, Figures, Data Availability Section)
Studies involving **human participants**: State details of **authority granting ethics approval** (IRB or equivalent committee(s), provide reference number for approval.	Yes	Materials and Methods
Studies involving **human participants**: Include a statement confirming that **informed consent** was obtained from all subjects and that the experiments conformed to the principles set out in the WMA Declaration of Helsinki and the Department of Health and Human Services Belmont Report.	Yes	Materials and Methods
Studies involving **human participants:** For publication of **patient photos**, include a statement confirming that consent to publish was obtained.	Not Applicable	
Studies involving experimental **animals**: State details of **authority granting ethics approval** (IRB or equivalent committee(s), provide reference number for approval. Include a statement of compliance with ethical regulations.	Yes	Materials and Methods
Studies involving **specimen and field samples:** State if relevant **permits** obtained, provide details of authority approving study; if none were required, explain why.	Not Applicable	

Dual Use Research of Concern (DURC)	Information included in the manuscript?	In which section is the information available? (Reagents and Tools Table, Materials and Methods, Figures, Data Availability Section)
Could your study fall under dual use research restrictions? Please check biosecurity documents and list of **select agents and toxins** (CDC): https://www.selectagents.gov/sat/list.htm	Not Applicable	
If you used a select agent, is the security level of the lab appropriate and reported in the manuscript?	Not Applicable	
If a study is subject to dual use research of concern regulations, is the name of the **authority granting approval and reference number** for the regulatory approval provided in the manuscript?	Not Applicable	

Reporting

The MDAR framework recommends adoption of discipline-specific guidelines, established and endorsed through community initiatives. Journals have their own policy about requiring specific guidelines and recommendations to complement MDAR.

Adherence to community standards	Information included in the manuscript?	In which section is the information available? (Reagents and Tools Table, Materials and Methods, Figures, Data Availability Section)
State if relevant guidelines or checklists (e.g., **ICMJE, MIBBI, ARRIVE, PRISMA**) have been followed or provided.	Not Applicable	
For **tumor marker prognostic studies,** we recommend that you follow the **REMARK** reporting guidelines (see link list at top right). See author guidelines, under 'Reporting Guidelines'. Please confirm you have followed these guidelines.	Not Applicable	
For **phase II and III randomized controlled trials**, please refer to the **CONSORT** flow diagram (see link list at top right) and submit the CONSORT checklist (see link list at top right) with your submission. See author guidelines, under 'Reporting Guidelines'. Please confirm you have submitted this list.	Not Applicable	

Data Availability

Data availability	Information included in the manuscript?	In which section is the information available? (Reagents and Tools Table, Materials and Methods, Figures, Data Availability Section)
Have **primary datasets** been deposited according to the journal's guidelines (see 'Data Deposition' section) and the respective accession numbers provided in the Data Availability Section?	Yes	Data Availability section
Were **human clinical and genomic datasets** deposited in a public access-controlled repository in accordance to ethical obligations to the patients and to the applicable consent agreement?	Yes	Data Availability section
Are **computational models** that are central and integral to a study available without restrictions in a machine-readable form? Were the relevant accession numbers or links provided?	Yes	Weblinks to publicly available datasets have been indicated in Materials and Methods section
If publicly available data were reused, provide the respective **data citations in the reference list**.	Yes	Weblinks to publicly available datasets have been indicated in Materials and Methods section
